# Amphiphilic Sialic Acid Derivatives as Potential Dual-Specific Inhibitors of Influenza Hemagglutinin and Neuraminidase

**DOI:** 10.3390/ijms242417268

**Published:** 2023-12-08

**Authors:** Eszter Boglárka Lőrincz, Mihály Herczeg, Josef Houser, Martina Rievajová, Ákos Kuki, Lenka Malinovská, Lieve Naesens, Michaela Wimmerová, Anikó Borbás, Pál Herczegh, Ilona Bereczki

**Affiliations:** 1 Department of Pharmaceutical Chemistry, University of Debrecen, H-4032 Debrecen, Hungary; elorincz01@gmail.com (E.B.L.); herczeg.mihaly@pharm.unideb.hu (M.H.); borbas.aniko@pharm.unideb.hu (A.B.); herczegh.pal@pharm.unideb.hu (P.H.); 2 Doctoral School of Pharmaceutical Sciences, University of Debrecen, H-4032 Debrecen, Hungary; 3 National Centre for Biomolecular Research, Masaryk University, 611 37 Brno, Czech Republic; josef.houser@ceitec.cz (J.H.); lenka.malinovska@ceitec.muni.cz (L.M.); michaw@chemi.muni.cz (M.W.); 4 Central European Institute of Technology, Masaryk University, 625 00 Brno, Czech Republic; 5 Department of Biochemistry, Faculty of Science, Masaryk University, 611 37 Brno, Czech Republic; rievajova.martina@mail.muni.cz; 6 Department of Applied Chemistry, University of Debrecen, H-4032 Debrecen, Hungary; kuki.akos@science.unideb.hu; 7 Rega Institute for Medical Research, KU Leuven, B-3000 Leuven, Belgium; lieve.naesens@kuleuven.be; 8 National Laboratory of Virology, University of Pécs, H-7624 Pécs, Hungary; 9 HUN-REN–UD Molecular Recognition and Interaction Research Group, University of Debrecen, H-4032 Debrecen, Hungary

**Keywords:** influenza, sialic acid, neuraminidase, hemagglutinin, aggregates

## Abstract

In the shadow of SARS-CoV-2, influenza seems to be an innocent virus, although new zoonotic influenza viruses evolved by mutations may lead to severe pandemics. According to WHO, there is an urgent need for better antiviral drugs. Blocking viral hemagglutinin with multivalent *N*-acetylneuraminic acid derivatives is a promising approach to prevent influenza infection. Moreover, dual inhibition of both hemagglutinin and neuraminidase may result in a more powerful effect. Since both viral glycoproteins can bind to neuraminic acid, we have prepared three series of amphiphilic self-assembling 2-thio-neuraminic acid derivatives constituting aggregates in aqueous medium to take advantage of their multivalent effect. One of the series was prepared by the azide-alkyne click reaction, and the other two by the thio-click reaction to yield neuraminic acid derivatives containing lipophilic tails of different sizes and an enzymatically stable thioglycosidic bond. Two of the three bis-octyl derivatives produced proved to be active against influenza viruses, while all three octyl derivatives bound to hemagglutinin and neuraminidase from H1N1 and H3N2 influenza types.

## 1. Introduction

In a typical influenza season, according to estimates, 250,000–650,000 deaths occur [1]. Among influenza viruses, only A types are known to cause pandemics. The most devastating influenza virus pandemic was the Spanish flu, which started in 1918. Estimations put the number of fatalities between 25 and 100 million [2], while the population of the world was under 2 billion at that time. Influenza viruses have a segmented negative-sense single-strand RNA genome. This segmented nature provides high variability for influenza. When two different types of influenza A strains (e.g., of human and animal origin) are present in the same host cell, an interchange of genomic RNA segments can occur, and new, sometimes more infective strains are evolved, encoding new antigenic proteins to which the human population has no preexisting immunity [3]. In this way, severe pandemics may occur with faster spread and higher death rates. In 2019, WHO published a list of the 10 most important health threats to humans; among these, they mentioned a new influenza pandemic that would happen sometime in the near future [4]. Moreover, WHO developed the Global Influenza Strategy for 2019–2030 [5] to increase global and national pandemic preparedness, to be ready for a threatening zoonotic influenza, and to improve seasonal influenza prevention and control all over the world. Although nowadays antiviral drugs are available to treat or prevent influenza, they are not highly effective [6]. Also, the virus can develop antiviral drug resistance quite easily, as happened in the period 2007–2009, when the oseltamivir-resistant H1N1 virus was spread all over the globe [7].

**Figure 1:**
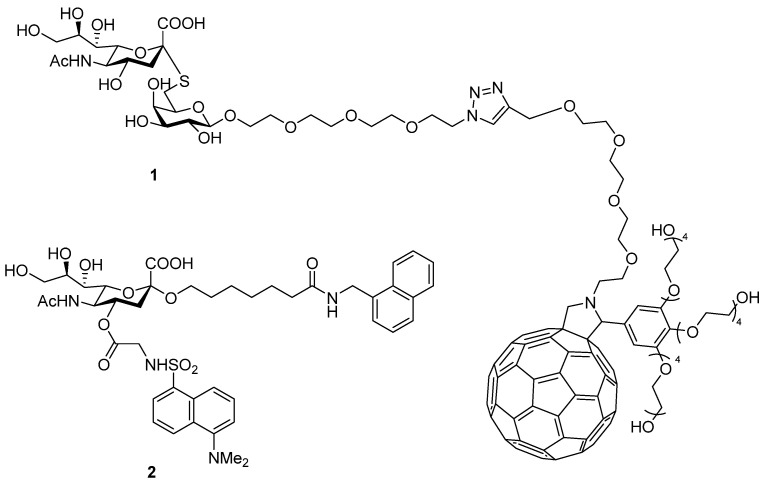
The literature results. Compound **1**: a fullerene conjugate containing a thiosialylated galactose disaccharide with aggregate-forming ability and neuraminidase inhibitory property [25]. Compound **2**: a fluorescent α-sialoside derivative with a lipophilic aglycone that shows high affinity for hemagglutinin but is not a substrate for viral neuraminidase [26].

Influenza A viruses can be characterized by their surface glycoproteins, hemagglutinin (HA) and neuraminidase (NA). Eighteen hemagglutinin and eleven neuraminidase subtypes are known in nature [8]. Replication of the influenza virus starts with the attachment of the virus to the host cell by its surface glycoprotein, hemagglutinin. HA recognizes and binds to the *N*-acetylneuraminic acid (sialic acid) terminals of host cell receptors [3]. After attachment, the virus enters the cell by endocytosis, and after the replication process, assembly of the new virus particles occurs at the cell membrane, where the new virions are still tied to the cells by their hemagglutinins attached to the sialic acid moieties of the cell surface receptors. Viral neuraminidase cleaves the terminal neuraminic acid moieties from the host cell surface to release new virions that can infect other cells [3]. The most important approved drugs against influenza are neuraminidase inhibitors, like oseltamivir and zanamivir. However, there is no hemagglutinin inhibitor in use, although the inhibition of the viral attachment would be a very effective strategy against influenza infection and mutation because the amino acid sequence of the sialic acid binding site of HA is highly conserved. Methyl α-glycoside of sialic acid is shown to bind weakly to hemagglutinin, while methyl β-glycoside does not bind at all [9]. To increase the strength of the binding between HA units and potential inhibitors, multivalency is a possible route [10]. Sialic acid units were conjugated to polymeric scaffolds such as polyacrylamide [11,12] or polyacrylate [13] to obtain multivalent ligands for hemagglutinin. Later, more biocompatible polysaccharides [14], chitosan [15], or polyglycerols [16] were used as polymeric scaffolds. A recently emerged strategy is to prepare sialic acid trimers with a special triangular arrangement inspired by the trimeric structure of the viral HA with three active sites [17,18,19]. In order to mimic the cell surface, liposomes functionalized with neuraminic acid were also prepared [20,21,22,23,24]. Some of the sialylated multivalent derivatives contain sialic acid conjugated to a galactose or an oligosaccharide unit similar to the receptor endings on the respiratory epithelial cells [14,15,21,24], but this oligosaccharide structure is not absolutely necessary [11,16,19]. Whitesides et al. showed that liposomes containing sialic acid monosaccharides were good inhibitors of hemagglutination [23]. Similarly to liposomes, multivalency can be achieved by simple amphiphilic derivatives, as sialic acid molecules equipped with lipophilic groups, including fullerene, can compose aggregates in aqueous medium (Figure 1 Compound **1**) [25]. The surface of the aggregates could mimic the host cell to trap the virus by binding its hemagglutinines. Moreover, it has been shown that large hydrophobic groups in the glycosidic position increased the binding affinity of sialic acid derivatives to hemagglutinin (Figure 1 Compound **2**) [26].

Based on the above results, we designed sialic acid derivatives equipped with lipophilic aglycones to obtain amphiphilic molecules capable of forming aggregates with multiple binding units. We attached double lipophilic chains, similar to phospholipids, of different lengths to sugar carrier molecules and connected neuraminic acid by thioglycosidic bond to this carrier through different linkers. The use of thiosialic acid is advantageous, as the thioglycosidic bond is known to be resistant to influenza virus neuraminidase [27]. Here, we report the synthesis, aggregation properties, antiviral activity, and binding affinity to influenza glycoproteins of the designed compounds.

## 2. Results and Discussion

### 2.1. Synthesis

**Scheme 1:**
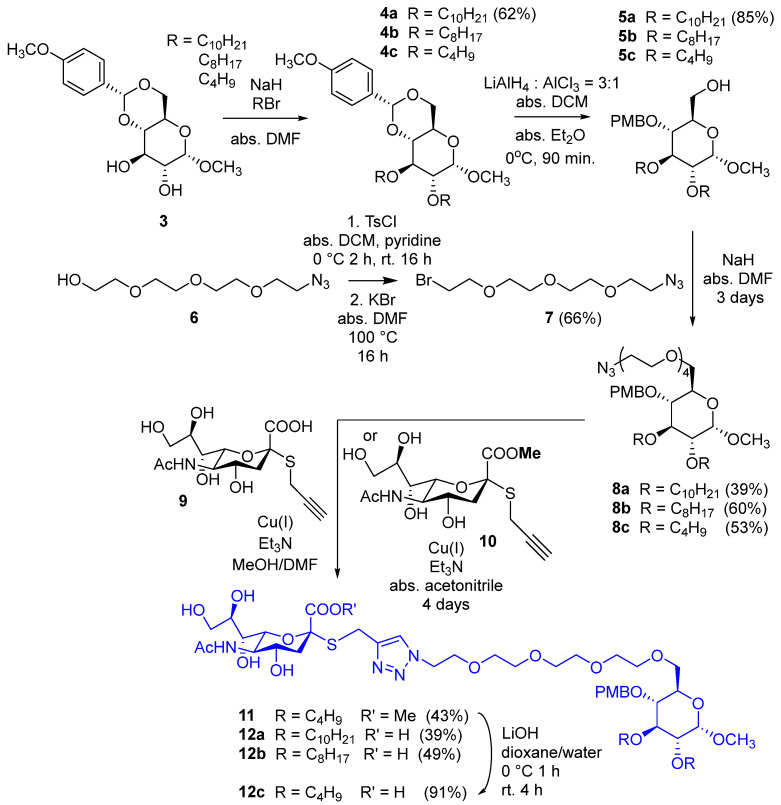
Synthesis of the amphiphilic sialic acid derivatives **12a**, **12b,** and **12c**. (**4b**, **5b** [29], **4c** and **5c** [30] were prepared according to the literature).

In order to synthesize amphiphilic neuraminic acid derivatives, first, a sugar carrier molecule was prepared, equipped with lipophilic side chains of different lengths (*n*-butyl, *n*-octyl, and *n*-decyl) (Scheme 1). Methyl 4,6-*O*-*p*-methoxybenzylidene-α-D-glucopyranoside (**3**) [28] was alkylated with decyl, octyl, and butyl bromide, obtaining **4a**, **4b** [29], and **4c** [30], respectively. Regioselective reductive cleavage of the 4,6-*O*-acetal ring afforded compounds **5a**–**c** [29,30], whose liberated 6-OH group was etherified with bromo-tetraethyleneglycol azide **7** (to yield the azido derivatives **8a**, **8b,** and **8c,** ready for 1,3-dipolar cycloaddition click reaction [31,32] with the appropriate alkyne derivative of sialic acid). The bromo derivative **7** was prepared from the monoazido tetraethylene glycol **6** [33] in two steps. Compounds **8a** and **8b** were reacted with the *S*-propargyl glycoside of *N*-acetylneuraminic acid **9** [34] in the presence of Cu(I)-iodide and triethylamine, to produce the amphiphilic derivatives **12a** and **12b,** respectively. The reaction of **8c** with the methyl ester form of the *S*-propargyl derivative of *N*-acetylneuraminic acid **10** [34], followed by ester hydrolysis of the resulting **11** with LiOH, yielded the amphiphilic derivative **12c**.

**Scheme 2:**
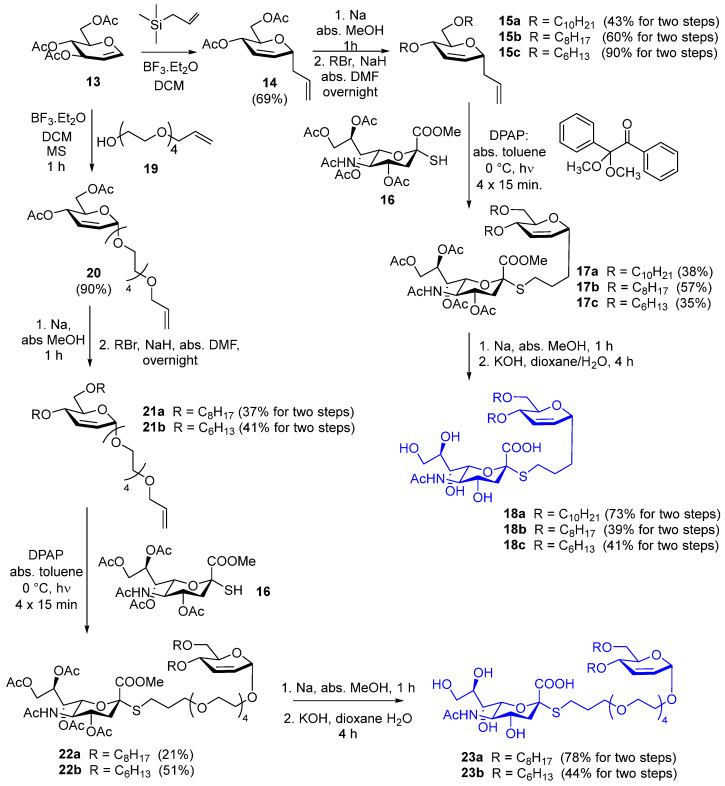
Synthesis of amphiphilic sialic acid derivatives from tri-*O*-acetyl-D-glucal.

A second set of amphiphilic neuraminic acid derivatives with different linker lengths was prepared via another method, the thio-click approach (Scheme 2). In this way, we were able to implement the synthesis in a much simpler and shorter route. Starting from tri-*O*-acetyl-D-glucal (**13**), a Ferrier reaction [35] with allyltrimethysilane was performed to obtain *C*-allyl glycoside (**14**) [36]; then, the acetyl protecting groups were removed by sodium methylate, and the free OH groups were etherified by decyl, octyl, and hexyl bromides to produce **15a**, **15b,** and **15c**, respectively. The 4,7,8,9-Tetra-*O*-acetyl-5-*N*-acetyl-2-thioneuraminic acid methyl ester **16** [37] was added to the double bond of the allyl groups of **15a**, **15b,** and **15c**, respectively, by photoinitiated thiol-ene click reaction using UV irradiation in the presence of the cleavable photoinitator 2,2-dimethoxy-2-phenylacetophenone (DPAP) [38]. Deacetylation followed by KOH-mediated hydrolysis of the methyl ester group provided the amphiphilic final products **18a**, **18b,** and **18c**.

Moreover, octyl and hexyl derivatives (**23a** and **23b**) with a tetraethylene glycol linker between the sugar carrier and sialic acid moieties were also synthesized by this shorter reaction route starting from **13**. Ferrier reaction of tri-*O*-acetyl-D-glucal (**13**) was carried out with monoallyl tetraethylene glycol (**19**) [39]; then, deacetylation was performed by sodium methylate, and the liberated OH groups were etherified by octyl or hexyl bromide to produce **21a** and **21b**, respectively. A 2-Thioneuraminic acid methyl ester **16** [37] was added to the double bond of the allyl groups of **21a** and **21b** by UV-light irradiation. After the removal of the acetyl-protecting groups and cleavage of the methyl ester, the desired amphiphilic derivatives **23a** and **23b** were obtained with good/acceptable yields.

### 2.2. Antiviral Evaluation

The anti-influenza activities of the synthesized derivatives were evaluated for H1N1 and H3N2 types influenza A and influenza B strains in Madin-Darby canine kidney (MDCK) cells. Two derivatives (**12b** and **18b**), equipped with octyl side chains, were effective against influenza A strains, while all other derivatives with shorter and longer side chains and the third bis-octyl derivative (**23b**) were ineffective (Table 1). None of the derivatives has activity against influenza B. The selectivity (i.e., the window between activity and cytotoxicity) of two active derivatives, **12b** and **18b,** was quite modest. Specifically, the ratio of CC_50_ to EC_50_, both determined by MTS assay, was, at best, a factor 4 for **12b** and a factor 5 for **18b**. This narrow selectivity could explain why we detected no activity against influenza B since the highest concentration tested (100 µM) was already associated with some cytotoxicity. Given that the receptor-binding properties of influenza A and B differ [40], the differences in anti-influenza activities of tested compounds are not surprising and were observed previously [29,34]. In order to obtain a deeper insight into the possible mechanism of action, the three bis-octyl derivatives were selected for further studies. Their binding affinity to hemagglutinin and neuraminidase was determined.

### 2.3. Interactions of Amphiphilic Sialic acid Derivatives with Hemagglutinin (HA) and Neuraminidase (NA)

**Figure 2:**
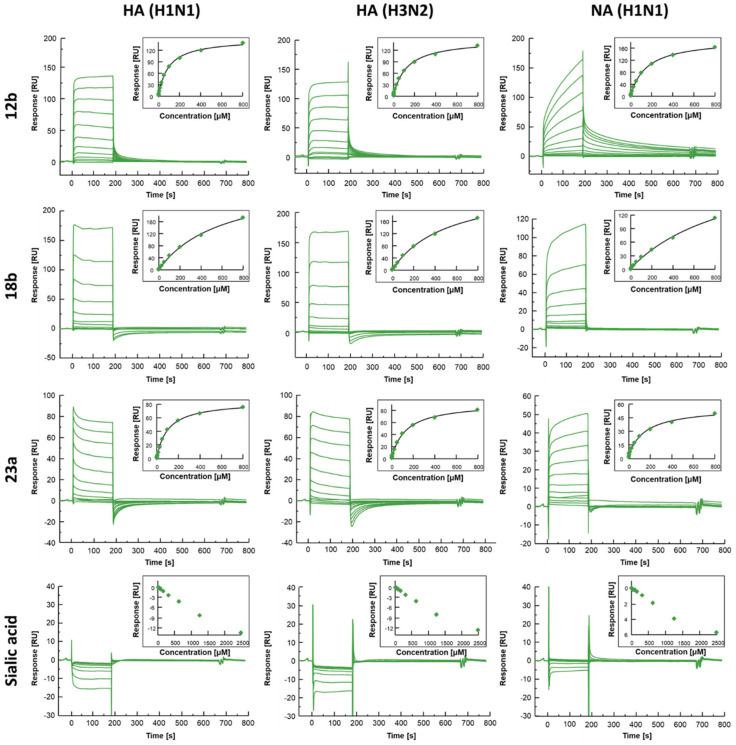
SPR sensorgrams. Single replicate of two-fold dilution row of each compound binding to immobilized proteins. Inset: Steady-state responses as green squares; one-site binding model fit in black (fit not shown for sialic acid).

The surface plasmon resonance (SPR) technique was used to determine the affinity of three octyl-containing thiosialic acid derivatives (**12b**, **18b**, and **23a**) toward two commercially available hemagglutinins from H1N1 and H3N2 influenza types and toward one neuraminidase of an H1N1 influenza type (Figure 2). All tested compounds are bound to all immobilized proteins, making them potential dual-specific inhibitors, similar to some compounds synthesized previously by others [41]. Compound **12b** was the strongest binding partner with an apparent affinity in the range of 10^−4^ M (Table 2), binding to HAs approximately twice as strongly as neuraminidase. Compounds **18b** and **23a** preferred neuraminidase over HAs. The interaction with **23a** was in the low mM range. The interaction with compound **18b** was the weakest, and for HAs, the K_D_(app) values could not be reliably calculated due to an increased response at higher compound concentrations. This could be caused by non-specific interactions with the biosensor. The compounds displayed a similarly strong interaction with H1N1 and H3N2 type HAs, with a slight preference for the latter. Sialic acid displayed increased binding to the blank channel (negative response on the differential curve), and therefore, the K_D_(app) toward sialic acid could not be determined. However, the K_D_(app) for compound **12b** toward HAs was 10 times lower than the previously reported value for sialic acid derivatives (such as α-methyl sialic acid or 4-*O*-acetylsialic acid) [9]. This can be explained either by additional contacts between HA and the compounds or by the formation of micelles or small aggregates. To investigate the system further, we performed the SPR experiment with immobilized HAs in the absence of DMSO in the running buffer (Supplementary Materials Figure S1). Under these conditions, the binding of **12b** did not show a significant difference compared to the experiment with DMSO present, while the observed affinity of **18b** and **23a** increased more than 10-fold (Supplementary Materials Table S1) compared to the experiment with 5% DMSO present in the running buffer. Since DMSO can affect micelle formation [42,43], we speculate that the different behavior observed in the absence of DMSO may be linked to the micelle-based multivalent effect.

### 2.4. Dynamic Light Scattering

Dynamic light scattering (DLS) measurements were performed for three neuraminic acid derivatives (**12a**, **12b,** and **12c**) with similar structures equipped with side chains of different lengths at two concentrations (1.0 mg/mL and 0.1 mg/mL) to study their aggregation properties. The average diameters of the particles are shown in Table 3. There is no substantial difference between the sizes of the clusters; however, the octyl derivative forms the smallest aggregates.

Size distributions can be found in the Supplementary Materials (Figures S2–S7).

## 3. Materials and Methods

### 3.1. Synthesis

#### 3.1.1. General Information

Methyl 4,6-*O*-*p*-methoxybenzylidene-α-D-glucopyranoside (**3**) [28], methyl 2,3-di-*O*-octyl- and methyl 2,3-di-*O*-butyl-4,6-*O*-*p*-methoxybenzylidene-α-D-glucopyranoside (**4b** [29] and **4c** [30]), methyl 2,3-di-*O*-octyl- and methyl 2,3-di-*O*-butyl-4-*O*-*p*-methoxybenzylidene-α-D-glucopyranoside (**5b** [29] and **5c** [30]), the mono-bromo-mono-*p*-toluenesulfonic acid derivative of ethylene glycol (**6**) [33], *N*-acetyl-2-*S*-propargyl-neuraminic acid (**9**) [34], methyl *N*-acetyl-2-*S*-propargyl-neuraminic acid (**10**) [34], 3-(4,6-di-*O*-acetyl-2,3-dideoxy-α-D-*erythro*-hex-2-enopyranosyl)-1-propene (**14**) [36], monoallyl-tetraethylene glycol (**19**), and methyl *N*-acetyl-4,7,8,9-tetra-*O*-acetyl-2-dezoxy-2-thio-neuraminic acid (**16**) [37] were synthesized according to the literature. Tri-*O*-acetyl-D-glucal (**13**) was purchased from Merck KGaA (Darmstadt, Germany).

TLC was performed on Kieselgel 60 F254 (Merck) with detection by immersion into ammonium molybdate-sulfuric acid solution followed by heating. Flash column chromatography was performed using Silica gel 60 (Merck 0.040–0.063 mm). The photoinitiated reactions were carried out in a borosilicate vessel by irradiation with a low-pressure Hg-lamp (Osram Supratec UV, HTC 150–211, 150 W, 230 V, R7s), giving maximum emission at 365 nm, without any caution to exclude air or moisture [38].

Conventional 1D and 2D ^1^H and ^13^C NMR spectra (^1^H-COSY, ^1^H-^13^C-HSQC, ^1^H-^13^C-HMBC) were recorded using a Bruker DRX-400 spectrometer (at 298 K or 300 K) and a 500 MHz (Bruker, Billerica, MA, USA) Avance II. spectrometer (at 310 K) equipped with a TXI probe head. Chemical shifts are referenced to Me_4_Si (0.00 ppm for ^1^H) and to the solvent residual signals (CDCl_3_, DMSO-d_6_ or pyridine-d_5_). NMR spectra of the synthesized compounds can be found in Supplementary Information.

MALDI-TOF MS (BIFLEX) analyses of the compounds were carried out in the positive reflectron mode using a BIFLEX III mass spectrometer (Bruker, Bremen, Germany) equipped with delayed-ion extraction. In all cases, 19 kV acceleration voltage was used with pulsed ion extraction (PIE^TM^). The positive ions were detected in the reflectron mode (20 kV). A nitrogen laser (337 nm, 3 ns pulse width, 10^6^–10^7^ W/cm^2^), operating at 4 Hz, was applied to produce laser desorption. Bruker Autoflex Speed mass spectrometer equipped with a time-of-flight (TOF) mass analyzer (Bruker, Bremen, Germany) was also used for MALDI-TOF MS (Autoflex) measurements. In all cases, 19 kV (ion source voltage 1) and 16.65 kV (ion source voltage 2) were used. For reflectron mode, 21 kV and 9.55 kV were applied as reflector voltage 1 and reflector voltage 2, respectively. A solid phase laser (355 nm, ≥100 μJ/pulse), operating at 500 Hz, was applied to produce laser desorption. A 2,5-Dihydroxybenzoic acid (DHB) was used as a matrix, and F_3_CCOONa as a cationising agent in DMF. ESI-QTOF MS measurements were carried out on a maXis II UHR ESI-QTOF MS instrument (Bruker, Bremen, Germany). The following parameters were applied for the electrospray ion source: capillary voltage: 3.5 kV; end plate offset: 500 V; nebulizer pressure: 0.4 bar; dry gas temperature: 200 °C; and dry gas flow rate: 4 L/min. Constant background correction was applied to each spectrum; the background was recorded before each sample by injecting the blank sample matrix (solvent). Na-formate calibrant was injected after each sample, which enabled external calibration during data evaluation. Mass spectra were recorded by micrOTOFcontrol version 2.2 (Bruker, Bremen, Germany) and processed by Compass DataAnalysis version 3.4 (Bruker Daltonik GmbH).

#### 3.1.2. Methyl 2,3-di-*O*-decyl-4,6-*O*-*p*-methoxybenzylidene-α-D-glucopyranoside (**4a**) 

Sodium hydride (180 mg, 4.48 mmol, 2 equiv./OH, 60% in mineral oil) was washed with hexane twice and then dried under argon. *N*,*N*-Dimethylformamide (10 mL) and methyl 4,6-*O*-*p*-methoxybenzylidene-α-D-glucopyranoside (**3**, 350 mg, 1.12 mmol) were added, and the mixture was stirred under argon atmosphere for 30 min. Then, *n*-decyl bromide (0.645 mL, 2.69 mmol, 1.2 equiv./OH) was added, and the mixture was stirred at room temperature overnight. Then, another portion of sodium hydride (50 mg) and *n*-decyl bromide (100 µL) were added, and the mixture was further stirred at room temperature for 2 days. Methanol (2 mL) and water (1 mL) were added successively to the reaction mixture, and it was stirred for 2 × 15 min. Then, the solvent was evaporated in a vacuum; the residue was dissolved in dichloromethane (100 mL) and was washed with water (2 × 10 mL). The organic phase was dried over Na_2_SO_4_ and filtered; then, the solvent was evaporated in a vacuum. The crude product was purified by flash column chromatography (hexane/ethyl acetate 95:5) to yield **4a** (414 mg, 62%) as a white powder. *R*_f_ = 0.73 (hexane/acetone 7:3); Mp: 80–83 °C; ^1^H NMR (400 MHz, CDCl_3_) δ (ppm) 7.41 (d, *J* = 8.7 Hz, 2H, arom.), 6.88 (d, *J* = 8.7 Hz, 2H, arom.), 5.49 (s, 1H, H*ac*), 4.79 (d, *J* = 3.6 Hz, 1H, H-1), 4.25 (dd, *J* = 9.8 Hz, *J* = 4.5 Hz, 1H, H-6a), 3.80 (s, 3H, PMP-OC*H*_3_), 3.78–3.61 (m, 7H, H-3, H-5, H-6b, 2 × decyl OC*H*_2_), 3.47 (t, *J* = 9.3 Hz, 1H, H-4), 3.43 (s, 3H, C-1-OC*H*_3_), 3.34 (dd, *J* = 9.3 Hz, *J* = 3.7 Hz, 1H, H-2), 1.62–1.52 (m, 4H, 2 × decyl C*H_2_*), 1.32–1.23 (m, 28H, 14 × decyl C*H_2_*), 0.89–0.86 (m, 6H, 2 × decyl C*H_3_*); ^13^C NMR (100 MHz, CDCl_3_) *δ* (ppm) 160.1, 130.2 (2C, 2 × C_q_ arom.), 127.5, 113.6 (4C, arom.), 101.3 (1C, C_ac_), 99.3 (1C, C-1), 82.1, 80.6, 78.4 (3C, skeleton carbons), 73.6, 72.4 (2C, 2 × decyl O*C*H_2_), 69.2 (1C, C-6), 62.4 (1C, C-5), 55.4 (2C, 2 × O*C*H_3_), 32.1, 30.5, 30.2, 29.8, 29.7, 29.6, 29.5, 26.3, 26.1, 22.8 (16C, 16 × decyl *C*H_2_), 14.3 (2C, 2 × decyl *C*H_3_); MALDI-TOF MS (BIFLEX): *m*/*z* calcd for C_35_H_60_O_7_Na^+^: 615.42 [M+Na]^+^; found: 615.35.

#### 3.1.3. Methyl 2,3-di-*O*-decyl-4-*O*-*p*-methoxybenzyl-α-D-glucopyranoside (**5a**) 

The solution of **4a** (400 mg, 0.6747 mmol) in the mixture of anhydrous dichloromethane (10 mL) and anhydrous diethyl ether (3.5 mL) was cooled to 0 °C under an argon atmosphere. Lithium aluminum hydride (115 mg, 3.036 mmol, 4.5 equiv.) was added in three portions. In another flask, anhydrous diethyl ether (3.5 mL) was cooled to 0 °C under an argon atmosphere, and aluminum chloride (135 mg, 1.01 mmol, 1.5 equiv.) was added. It was stirred for 10 min and then added to the solution of **4a**. The reaction mixture was stirred at 0 °C under an argon atmosphere for 90 min. Ethyl acetate (15 mL) was added, and the mixture was stirred for 10 min; then, water (4 mL) was added, and it was stirred for another 10 min. The reaction mixture was filtered through Celite^®^ (Sigma-Aldrich, Burlington, MA, USA) and washed with ethyl acetate (2 × 7 mL). The filtrate was diluted with ethyl acetate (50 mL) and washed with water (2 × 10 mL). The organic layer was dried over Na_2_SO_4_, filtered, and the solvent was evaporated. The residue was purified by flash column chromatography (hexane/acetone 9:1) to yield **5a** (340 mg, 85%) as a colorless syrup. *R*_f_ = 0.26 (hexane/acetone 8:2); ^1^H NMR (400 MHz, CDCl_3_) *δ* (ppm) 7.28–7.26 (m, 2H, arom.), 6.89–6.87 (m, 2H, arom.), 4.82 (d, *J* = 10.7 Hz, 1H, PMB-C*H*_2_a), 4.76 (d, *J* = 3.5 Hz, 1H, H-1), 4.57 (d, *J* = 10.7 Hz, 1H, PMB-C*H*_2_b), 3.88–3.84 (m, 1H, decyl C*H*_2_a), 3.80 (s, 3H, PMB-OC*H*_3_), 3.76–3.57 (m, 7H, decyl C*H*_2_b, decyl C*H*_2_, H-3, H-5, H-6a,b), 3.41 (t, *J* = 9.3 Hz, 1H, H-4), 3.38 (s, 3H, C-1-OC*H*_3_), 3.27 (dd, *J* = 9.6 Hz, *J* = 3.5 Hz, 1H, H-2), 1.70 (t, *J* = 6.3 Hz, 1H, C-6-OH), 1.64–1.59 (m, 4H, 2 × decyl C*H*_2_,), 1.37–1.25 (m, 28H, 14 × decyl C*H*_2_), 0.90–0.86 (m, 6H, 2 × decyl C*H*_3_); ^13^C NMR (100 MHz, CDCl_3_) *δ* (ppm) 159.5, 130.6 (2C, 2 × C_q_ arom.), 129.9, 114.0 (4C, arom.), 98.2 (1C, C-1), 81.8, 81.0, 77.3 (3C, skeleton carbons), 74.7, 73.9, 71.9 (3C, 2 × decyl O*C*H_2_, PMB-*C*H_2_), 70.7 (1C, C-5), 62.2 (1C, C-6), 55.4, 55.2 (2C, 2 × O*C*H_3_), 32.1, 30.8, 30.2, 29.8, 29.6, 29.5, 26.4, 26.2, 22.8 (16C, 16 × decyl *C*H_2_), 14.2 (2C, 2 × decyl *C*H_3_); MALDI-TOF MS (BIFLEX): *m*/*z* calcd for C_35_H_62_O_7_Na^+^: 617.44 [M+Na]^+^; found: 617.49.

#### 3.1.4. Compound **7**

Tetratehylene glycol monoazide (**6**, 5 g, 22.8 mmol) was dissolved in anhydrous dichloromethane (50 mL); then, anhydrous pyridine (12 mL) was added, and the mixture was cooled to 0 °C. Tosyl chloride (13.04 g, 68.4 mmol. 3 equiv.) was dissolved in anhydrous dichloromethane (100 mL) and was added dropwise to the solution of **6** in 2 h. The mixture was stirred overnight. After 16 h, dichloromethane was evaporated in a vacuum; distilled water (20 mL) was added to the residue, and the mixture was stirred for 2 h. The solvent was evaporated in a vacuum, and the residue was dissolved in dichloromethane (500 mL); then, it was washed with 10% aqueous NaHSO_4_ solution (2 × 50 mL) and saturated aqueous NaHCO_3_ solution (2 × 50 mL). The organic phase was dried over Na_2_SO_4_, then filtered, and the solvent was evaporated (*R*_f_ = 0.51; hexane/acetone 6:4). The residue was dissolved in anhydrous dimethylformamide (60 mL); powdered KBr (8.14 g, 68.4 mmol, 3 equiv.) was added, and the mixture was stirred overnight. The solvent was evaporated in a vacuum, and toluene was added and evaporated. The residue was dissolved in ethyl acetate (600 mL); it was washed with brine (2 × 50 mL); the organic phase was dried over Na_2_SO_4_, then filtered, and the solvent was evaporated in a vacuum. The residue was purified by flash column chromatography to yield **7** (4.25 g, 66% for two steps) as a yellowish syrup. *R*_f_ = 0.63 (hexane/acetone 6:4); MALDI-TOF MS (Autoflex): *m*/*z* calcd for C_8_H_16_BrN_3_O_3_Na^+^: 304.027 [M+Na]^+^; found: 303.976.

#### 3.1.5. Compound **8a**

Sodium hydride (40 mg, 1.0 mmol, 2 equiv., 60% in mineral oil) was washed with hexane and dried under argon. Anhydrous *N*,*N*-dimethylformamide (10 mL) and **5a** (297.5 mg, 0.5 mmol, 1 equiv.) were added, and the mixture was stirred for 30 min under an argon atmosphere. Then, compound **7** (170 mg, 0.6 mmol, 1.2 equiv.) was added, and the reaction mixture was stirred at 40 °C under an argon atmosphere. After one day, other portions of sodium hydride (20 mg, 0.5 mmol, 1 equiv., 60% in mineral oil) and **7** (85 mg, 0.3 mmol, 0.6 equiv.) were added. After two more days of stirring, methanol (1 mL) and water (1 mL) were added successively, and the reaction mixture was stirred for 30 min. Then, the solvent was evaporated in a vacuum, and the residue was purified by flash column chromatography (hexane/acetone 9:1) to yield **8a** (156 mg, 39%) as a yellowish syrup. *R*_f_ = 0.38 (hexane/acetone 8:2); ^1^H NMR (400 MHz, CDCl_3_) *δ* (ppm) 7.27 (d, *J* = 8.6 Hz, 2H, arom.), 6.87 (d, *J* = 8.6 Hz, 2H, arom.), 4.80 (d, *J* = 10.5 Hz, 1H, PMB-C*H*_2_), 4.78 (d, *J* = 3.6 Hz, 1H, H-1), 4.56 (d, *J* = 10.4 Hz, 1H, PMB-C*H*_2_), 3.86–3.81 (m, 1H, decyl C*H*_2_a), 3.80 (s, 3H, PMB-OC*H*_3_), 3.74–3.56 (m, 21H, 7 × OC*H*_2_ TEG, decyl C*H*_2_b, decyl C*H*_2_, H-3, H-5, H-6a,b), 3.49 (t, *J* = 9.2 Hz, 1H, H-4), 3.37 (s, 3H, C-1-OC*H*_3_), 3.36–3.35 (m, 2H, C*H*_2_-N_3_), 3.30 (dd, *J* = 9.7 Hz, *J* = 3.5 Hz, 1H, H-2), 1.63–1.58 (m, 4H, 2 × decyl C*H*_2_), 1.34–1.25 (m, 28H, 14 × decyl C*H*_2_), 0.89–0.86 (m, 6H, 2 × decyl C*H*_3_); ^13^C NMR (100 MHz, CDCl_3_) *δ* (ppm) 159.3, 130.9 (2C, 2 × C_q_ arom.), 129.7, 113.9 (4C, arom.), 98.2 (1C, C-1), 81.8, 80.9, 77.5, 70.2 (4C, skeleton carbons), 74.8, 73.8, 71.9, 71.0, 70.8, 70.7, 70.6, 70.1, 70.0 (11C, 7 × O*C*H_2_ TEG, 2 × O*C*H_2_ decyl, PMB-O*C*H_2_, C-6), 55.4, 55.1 (2C, 2 × O*C*H_3_), 50.8 (1C, *C*H_2_-N_3_) 32.0, 30.7, 30.2, 29.9, 29.8, 29.7, 29.6, 29.5, 26.4, 26.1, 22.8 (14C, 7 × decyl *C*H_2_), 14.2 (2C, 2 × decyl *C*H_3_); MALDI-TOF MS (Autoflex): *m*/*z* calcd for C_43_H_77_N_3_O_10_Na^+^: 818.5501 [M+Na]^+^; found: 818.5509.

#### 3.1.6. Compound **8b**

Compound **5b** (269 mg, 0.5 mmol, 1 equiv.) was alkylated with the bromide derivative **7** (170 mg, 0.6 mmol, 1.2 equiv.) according to the synthesis of **8a**. The crude product was purified by flash column chromatography (hexane/acetone 8:2) to yield **8b** (314 mg, 60%) as a yellowish syrup. *R*_f_ = 0.46 (hexane/acetone 7:3); ^1^H NMR (400 MHz, CDCl_3_) *δ* (ppm) 7.26 (d, *J* = 8.1 Hz, 2H, arom.), 6.87 (d, *J* = 8.4 Hz, 2H, arom.), 4.80 (d, *J* = 10.8 Hz, 1H, PMB-C*H*_2_), 4.77 (d, *J* = 3.4 Hz, 1H, H-1), 4.57 (d, *J* = 10.4 Hz, 1H, PMB-C*H*_2_), 3.88–3.80 (m, 1H, otyl OC*H*_2a_), 3.80 (s, 3H, PMB-OC*H*_3_), 3.74–3.56 (m, 21H, 7 × OC*H*_2_ TEG, octyl C*H*_2_b, octyl C*H*_2_, H-3, H-5, H-6a,b), 3.49 (t, *J* = 9.2 Hz, 1H, H-4), 3.37 (s, 3H, C-1-OC*H*_3_), 3.36–3.35 (m, 2H, C*H*_2_-N_3_), 3.30 (dd, *J* = 9.6 Hz, *J* = 3.2 Hz, 1H, H-2), 1.61–1.58 (m, 4H, 2 × octyl C*H*_2_), 1.33–1.26 (m, 20H, 10 × octyl C*H*_2_), 0.88–0.84 (m, 6H, 2 × octyl C*H*_3_); ^13^C NMR (100 MHz, CDCl_3_) *δ* (ppm) 159.4, 130.9 (2C, 2 × C_q_ arom.), 129.7, 113.9 (4C, arom.), 98.2 (1C, C-1), 81.9, 80.9, 77.5, 70.2 (4C, skeleton carbons), 74.8, 73.9, 71.9, 71.1, 70.8, 70.7, 70.6, 70.1, 70.0 (11C, 7 × O*C*H_2_ TEG, 2 × O*C*H_2_ octyl, PMB-O*C*H_2_, C-6), 55.4, 55.2 (2C, 2 × O*C*H_3_), 50.8 (1C, *C*H_2_-N_3_), 32.0, 30.8, 30.2, 29.9, 29.8, 29.7, 29.6, 29.5, 29.4, 26.4, 26.1, 22.8 (10C, 5 × octyl *C*H_2_), 14.2 (2C, 2 × octyl *C*H_3_); MALDI-TOF MS (BRUKER): *m*/*z* calcd for C_39_H_69_N_3_O_10_Na^+^: 762.49 [M+Na]^+^; found: 762.32.

#### 3.1.7. Compound **8c**

Compound **5c** (213 mg, 0.5 mmol, 1 equiv.) was alkylated with the bromide derivative **7** (170 mg, 0.6 mmol, 1.2 equiv.) according to the synthesis of **8a**. The crude product was purified by flash column chromatography (hexane/acetone 7:3) to yield **8c** (167 mg, 53%) as a yellowish syrup. *R*_f_ = 0.28 (hexane/acetone 8:2); ^1^H NMR (400 MHz, CDCl_3_) *δ* (ppm) 7.27 (d, *J* = 8.5 Hz, 2H, arom.), 6.88 (d, *J* = 8.5 Hz, 2H, arom.), 4.80 (d, *J* = 10.4 Hz, 1H, PMB-C*H*_2_), 4.78 (d, *J* = 3.4 Hz, 1H, H-1), 4.58 (d, *J* = 10.4 Hz, 1H, PMB-C*H*_2_), 3.88–3.84 (m, 1H, butyl OC*H*_2_a), 3.79 (s, 3H, PMB-OC*H*_3_), 3.78–3.58 (m, 21H, 7 × OC*H*_2_ TEG, butyl OC*H*_2_b, butyl OC*H*_2_, H-3, H-5, H-6a,b), 3.51 (t, *J* = 9.2 Hz, 1H, H-4), 3.38 (s, 3H, C-1-OC*H*_3_), 3.36–3.34 (m, 2H, -C*H*_2_-N_3_), 3.31 (dd, *J* = 9.7 Hz, *J* = 3.5 Hz, 1H, H-2), 1.64–1.55 (m, 4H, 2 × butyl C*H*_2_), 1.43–1.36 (m, 4H, 2 × butyl C*H*_2_), 0.92 (t, *J* = 7.4 Hz, 6H, 2 × butyl C*H*_3_); ^13^C NMR (100 MHz, CDCl_3_) *δ* (ppm) 159.2, 130.7 (2C, 2 × C_q_ arom.), 129.6, 113.7 (4C, arom.), 98.0 (1C, C-1), 81.6 (1C, C-3), 80.7 (1C, C-2), 77.3 (1C, C-4), 74.6 (1C, PMB-*C*H_2_), 73.3, 71.2, 70.8, 70.6, 70.4, 69.9, 69.8 (10C, 7 × O*C*H_2_ TEG, 2 × O*C*H_2_ butyl, C-6), 70.0 (1C, C-5), 55.2 (1C, PMB-O*C*H_3_), 54.9 (1C, C-1-O*C*H_3_), 50.5 (1C, *C*H_2_-N_3_), 32.6, 32.0, 19.3, 19.1 (4C, 4 × butyl *C*H_2_), 14.0, 13.8 (2C, 2 × butyl *C*H_3_); MALDI-TOF MS (Autoflex): *m*/*z* calcd for C_31_H_53_N_3_O_10_Na^+^: 650.3623 [M+Na]^+^; found: 650.3634.

#### 3.1.8. Compound **12a**

Compound **9** (50 mg, 0.138 mmol, 1 equiv.) was dissolved in anhydrous methanol (3 mL), and **8a** (131 mg, 0.165 mmol, 1.2 equiv.) in anhydrous *N*,*N*-dimethylformamide (3 mL) was added. Then, triethylamine (40 µL, 0.275 mmol, 2 equiv.) and copper(I) iodide (3 mg, 0.0138 mmol, 0.1 equiv.) were added. The reaction mixture was stirred at room temperature overnight, then other portions of triethylamine (40 µL, 0.275 mmol, 2 equiv.) and copper(I) iodide (3 mg, 0.0138 mmol, 0.1 equiv.) were added, and the mixture was stirred at 40 °C for 2 days and at 50 °C for 1 day. Thereafter, the solvent was evaporated in a vacuum, and the residue was purified by flash column chromatography (toluene/methanol 8:2 containing 0.1% acetic acid) to yield **12a** (62 mg, 39%) as a yellowish syrup. *R*_f_ = 0.18 (toluene/methanol 7:3 containing 0.1% acetic acid); ^1^H NMR (400 MHz, DMSO-d_6_) *δ* (ppm) 8.82 (d, *J* = 7.3 Hz, 1H, N*H*), 7.96 (s, 1H, triazole C=C*H*), 7.22 (d, *J* = 8.6 Hz, 2H, arom.), 6.89 (d, *J* = 8.6 Hz, 2H, arom.), 4.74 (d, *J* = 3.3 Hz, 1H, H-1), 4.64 (d, *J* = 10.7 Hz, 1H, PMB-C*H*_2_a), 4.49 (d, *J* = 10.8 Hz, 1H, PMB-C*H*_2_b), 4.44 (t, *J* = 5.3 Hz, 2H, NC*H*_2_), 4.00–3.92 (m, 2H, SC*H*_2_,), 3.89–3.20 (m, 33H, 7 × OC*H*_2_ TEG, 2 × decyl OC*H*_2_, H-3, H-4, H-5, H-6a,b, H-4′, H-5′, H-6′, H-7′, H-8′, H-9′a,b, PMB-OC*H*_3_), 3.17 (s, 3H, C-1-OC*H*_3_), 3.16–3.13 (m, 1H, H-2), 2.72 (dd, *J* = 11.2 Hz, *J* = 3.8 Hz, 1H, H-3′a), 1.89 (s, 3H, NAc C*H*_3_), 1.50–1.43 (m, 4H, 2 × decyl C*H*_2_), 1.38 (t, *J* = 11.4 Hz, 1H, H-3′b), 1.24–1.21 (m, 32H, 4 × O*H*, 14 × decyl C*H*_2_), 0.87–0.83 (m, 6H, 2 × decyl C*H*_3_); ^13^C NMR (100 MHz, DMSO-d_6_) *δ* (ppm) 177.0 (1C, Ac C=O), 173.0 (1C, C-1′), 159.2 (1C, C_q_ arom.), 144.6 (1C, triazole *C*=CH), 131.1 (1C, C_q_ arom.), 129.7 (2C, arom.), 124.0 (1C, triazole C=*C*H), 114.0 (2C, arom.), 98.4 (1C, C-1), 85.7 (1C, C-2′), 81.7 (1C, C-3), 80.4 (1C, C-2), 77.5 (1C, C-4), 75.6 (1C, C-6′), 74.0 (1C, PMB-*C*H_2_), 73.0 (1C, O*C*H_2_ decyl), 71.7 (1C, C-8′), 70.6 (1C, O*C*H_2_ decyl), 70.3, 70.2, 70.1, 70.0, 69.9, 69.1 (8C, C-6, 7 × O*C*H_2_ TEG), 69.7 (2C, C-5, C-7′), 67.6 (1C, C-4′), 63.8 (1C, C-9′), 55.5 (1C, PMB-O*C*H_3_), 54.9 (1C, C-1-O*C*H_3_), 53.7 (1C, C-5′), 49.7 (1C, N*C*H_2_), 42.5 (1C, C-3′), 31.8, 30.5, 30.1, 29.6, 29.5, 29.4, 29.2, 26.2, 26.1 (14C, 14 × decyl *C*H_2_), 23.9 (1C, S*C*H_2_), 22.3 (1C, NAc C*H*_3_), 22.6 (2C, 2 × decyl *C*H_2_), 14.4 (2C, 2 × decyl *C*H_3_); MALDI-TOF MS (Autoflex): *m*/*z* calcd for C_57_H_97_N_4_O_18_SNa_2_^+^: 1203.631 [M-H+2Na]^+^; found: 1203.638.

#### 3.1.9. Compound **12b**

Compound **9** (50 mg, 0.138 mmol) was reacted with compound **8b** (122 mg, 0.165 mmol, 1.2 equiv.) in the presence of triethylamine (40 µL, 0.2752 mmol, 2 equiv.) and copper(I) iodide (3 mg, 0.0138 mmol, 0.1 equiv.) according to the procedure applied for the synthesis of **12a**. The crude product was purified by column chromatography (1st Silicagel 60, 40–63 μm, dichloromethane/methanol 92:8, 2nd Sephadex LH20, MeOH) to yield **12b** (50 mg, 33%) as a yellowish syrup. *R*_f_ = 0.22 (toluene/methanol 7:3 containing 0.1% acetic acid); ^1^H NMR (400 MHz, Pyridine-d_5_) *δ* (ppm) 9.21 (s, 1H, N*H*), 8.27 (s, 1H, triazole C=C*H*), 7.63 (d, *J* = 8.4 Hz, 2H, arom.), 7.18 (d, *J* = 8.4 Hz, 2H, arom.), 5.18–5.14 (m, 2H, H-1, PMB-C*H*_2_a), 4.96 (d, *J* = 10.8 Hz, 1H, PMB-C*H*_2_b), 4.69–4.64 (m, 5H, H-5′, NC*H*_2_, SC*H*_2_,), 4.51–4.48 (m, 1H, H-9′a), 4.39–4.37 (m, 1H, H-9′b), 4.14–3.65 (m, 31H, 7 × OC*H*_2_ TEG, 2 × octyl OC*H*_2_, H-2, H-3, H-4, H-5, H-6a,b, H-4′, H-6′, H-7′, H-8′, PMB-OC*H*_3_), 3.56 (s, 3H, C-1-OC*H*_3_), 2.45–2.42 (m, 1H, H-3′a), 2.14 (s, 3H, NAc C*H*_3_), 1.83–1.28 (m, 29H, H-3′b, 4 × O*H*, 12 × octyl C*H*_2_), 0.91–0.90 (m, 6H, 2 × octyl C*H*_3_); ^13^C NMR (100 MHz, Pyridine-d_5_) *δ* (ppm) 175.1 (1C, Ac C=O), 172.7 (1C, C-1′), 160.7 (1C, C_q_ arom.), 144.8 (1C, triazole *C*=CH), 132.6 (1C, C_q_ arom.), 131.0 (2C, arom.), 115.2 (2C, arom.), 99.3 (1C, C-1), 86.7 (1C, C-2′), 83.3 (1C, C-3), 82.0 (1C, C-2), 79.0 (1C, C-4), 77.8 (1C, C-6′), 75.7 (1C, PMB-*C*H_2_), 74.7 (1C, O*C*H_2_ octyl), 74.0 (1C, C-8′), 72.1 (1C, O*C*H_2_ octyl), 71.9 (2C, C-5, C-7′), 71.7, 71.6, 71.5, 70.5 (8C, C-6, 7 × O*C*H_2_ TEG), 70.2 (1C, C-4′), 65.4 (1C, C-9′), 56.4 (1C, PMB-O*C*H_3_), 56.2 (1C, C-1-O*C*H_3_), 55.1 (1C, C-5′), 51.2 (1C, N*C*H_2_), 44.2 (1C, C-3′), 33.1, 33.0, 32.0, 30.8, 30.7, 30.5, 27.6, 27.4 (10C, 10 × octyl *C*H_2_), 25.6 (1C, S*C*H_2_), 23.9 (1C, NAc C*H*_3_), 23.8 (2C, 2 × octyl *C*H_2_), 15.2 (2C, 2 × octyl *C*H_3_); ESI-QTOF MS: *m*/*z* calcd for C_53_H_89_N_4_O_18_S^−^: 1101.589 [M-H]^−^; found: 1101.599.

#### 3.1.10. Compound **12c**

Compounds **10** (70 mg, 0.185 mmol, 1 equiv.) and **8c** (138 mg, 0.278 mmol, 1.5 equiv.) were dissolved in anhydrous acetonitrile (10 mL). Triethylamine (52 µL, 0.37 mmol, 2 equiv.) and copper(I) iodide (3.5 mg, 0.0185 mmol, 0.1 equiv.) were added. The reaction mixture was stirred at room temperature overnight; then, additional triethylamine (26 µL, 0.185 mmol, 1 equiv.) and copper(I) iodide (3.5 mg, 0.0185 mmol, 0.1 equiv.) were added. After 3 more days, the solvent was evaporated in a vacuum, and the residue was purified by flash column chromatography (dichloromethane/methanol 95:5) to yield **11** (73.2 mg, 43%) as a yellowish syrup (*R*_f_ = 0.62 in dichloromethane/methanol 7:3 containing 0.1% acetic acid).

Compound **11** (60 mg, 0.06 mmol) was dissolved in anhydrous dioxane (9 mL) and water (1 mL), and the solution was cooled to 0 °C. Then, a 0.06 M solution of lithium hydroxide in water (300 µL, 0.179 mmol, 3 equiv) was added. The reaction mixture was stirred at 0 °C for 1 h and at room temperature for 4 h. Thereafter, the solvent was evaporated, and the residue was purified by flash column chromatography (toluene/methanol 7:3) to yield **12c** (54 mg, 91%) as a yellowish syrup. *R*_f_ = 0.64 (toluene/methanol 6:4); ^1^H NMR (400 MHz, Pyridine-D_5_) *δ* (ppm) 8.06 (s, 1H, triazole C=C*H*), 7.51 (d, *J* = 8.0 Hz, 2H, arom.), 7.07 (d, *J* = 8.1 Hz, 2H, arom.), 6.16 (s, 1H, N*H*), 5.06–4.82 (m, 3H, H-1, PMB-C*H*_2_a,b), 4.73–4.44 (m, 4H, SC*H*_2_, NC*H*_2_), 4.01–3.53 (m, 33H, 7 × OC*H*_2_ TEG, 2 × butyl OC*H*_2_, H-3, H-4, H-5, H-6a,b, H-4′, H-5′, H-6′, H-7′, H-8′, H-9′a,b, PMB-OC*H*_3_), 3.45–3.42 (m, 1H, H-2), 3.39 (s, 3H, C-1-OC*H*_3_), 2.34–2.30 (m, 1H, H-3′a), 2.03 (s, 3H, NAc), 1.66–1.54 (m, 5H, H-3′b, 2 × butyl C*H*_2_), 1.44–1.37 (m, 4H, 2 × butyl C*H*_2_), 0.89–0.83 (m, 6H, 2 × butyl C*H*_3_); ^13^C NMR (100 MHz, Pyridine-D_5_) *δ* (ppm) 177.4 (1C, Ac C=O), 174.0 (1C, C-1′), 160.1 (1C, C_q_ arom.), 144.5 (1C, triazole *C*=CH), 132.2 (1C, C_q_ arom.), 130.3 (2C, arom.), 124.7 (1C, triazole C=*C*H), 114.6 (2C, arom.), 98.7 (1C, C-1), 86.7 (1C, C-2′), 82.7 (1C, C-3), 81.6 (1C, C-2), 78.5 (1C, C-4), 77.3 (1C, C-6′), 75.0 (1C, PMB-*C*H_2_), 73.5 (1C, O*C*H_2_ butyl), 73.0 (1C, C-8′), 71.6 (1C, O*C*H_2_ butyl), 71.4 (1C- C-5), 71.2, 71.1, 71.0, 70.9 (9C, C-7′, 7 × O*C*H_2_ TEG, C-6), 68.1 (1C, C-4′), 63.1 (1C, C-9′), 55.6 (1C, PMB-O*C*H_3_), 55.4 (1C, C-1-O*C*H_3_), 54.5 (1C, C-5′), 50.6 (1C, N*C*H_2_), 41.7 (1C, C-3′), 33.5, 32.9 (2C, 2 × butyl *C*H_2_), 25.1 (1C, S*C*H_2_), 23.4 (1C, NAc), 20.1, 19.9 (2C, 2 × butyl *C*H_2_), 14.6, 14.4 (2C, 2 × butyl *C*H_3_); ESI-QTOF MS: *m*/*z* calcd for C_45_H_74_N_4_O_18_SH^+^: 991.479 [M+H]^+^; found: 991.490.

#### 3.1.11. Compound **15a**

Compound **14** (500 mg, 1.97 mmol) was dissolved in anhydrous methanol (10 mL); small pieces of sodium were added to make the solution alkaline (pH ~ 11). The reaction mixture was stirred for 1 h; then, Serdolit Red H^+^ ion exchange resin was added to make the pH neutral. Thereafter, the reaction mixture was filtered, and the solvent was evaporated. A dichloromethane/methanol 9:1 mixture was used for TLC (*R*_f_ = 0.60).

Sodium hydride (315 mg, 7.88 mmol, 2 equiv./OH, 60% in mineral oil) was washed with hexane and dried under an argon atmosphere; then, anhydrous *N*,*N*-dimethylformamide (DMF) (3 mL) was added. The crude product of the previous step was dissolved in anhydrous DMF (2 mL) and added to the sodium hydride. The reaction mixture was stirred under an argon atmosphere for 20 min; then, decyl bromide (980 μL, 4.72 mmol, 1.2 equiv/OH) was added in two portions, and the reaction was further stirred at room temperature overnight. Then, methanol (1 mL) and water (1 mL) were added successively, and the reaction mixture was stirred for 30 min. The solvent was evaporated, and the residue was purified by flash column chromatography (hexane/ethylacetate 97:3) to yield **15a** (378 mg, 43%) as a colorless syrup. *R*_f_ = 0.89 (hexane/ethylacetate 8:2); ^1^H NMR (400 MHz, CDCl_3_) *δ* (ppm) 5.92–5.89 (m, 1H, H-3), 5.88–5.82 (m, 1H, allyl C*H*=CH_2_), 5.82–5.79 (m, 1H, H-2), 5.12–5.05 (m, 2H, allyl CH=C*H*_2_), 4.23 (ddd, *J* = 8.1 Hz, *J* = 6.2 Hz, *J* = 2.2 Hz, 1H, H-1), 3.82 (dq, *J* = 7.3 Hz, *J* = 1.9 Hz, 1H, H-4), 3.69–3.65 (m, 1H, H-5), 3.64–3.61 (m, 3H, decyl OC*H*_2_a, H-6a,b), 3.54–3.39 (m, 3H, decyl C*H*_2_b, decyl C*H*_2_), 2.51–2.44 (m, 1H, allyl C*H*_2_a), 2.32–2.25 (m, 1H, allyl C*H*_2_b), 1.63–1.53 (m, 4H, 2 × decyl C*H*_2_), 1.30–1.26 (m, 28H, 14 × decyl C*H*_2_), 0.88 (t, *J* = 6.7 Hz, 6H, 2 × decyl C*H*_3_); ^13^C NMR (100 MHz, CDCl_3_) *δ* (ppm) 134.8 (1C, allyl C*H*=CH_2_), 130.7 (1C, C-2), 126.3 (1C, C-3), 117.3 (1C, allyl CH=C*H*_2_), 72.4 (1C, C-1), 71.8 (1C, decyl O*C*H_2_), 71.2 (1C, C-5), 70.6 (1C, C-4), 70.2 (1C, C-6), 69.5 (1C, decyl O*C*H_2_), 38.1 (1C, allyl *C*H_2_), 32.0, 30.2, 29.8, 29.7, 29.6, 29.5, 26.3, 22.8 (16C, 16 × decyl *C*H_2_), 14.2 (2C, 2 × decyl *C*H_3_); MALDI-TOF MS (Autoflex): *m*/*z* calcd for C_29_H_54_O_3_Na^+^: 473.3965 [M+Na]^+^; found: 473.3960.

#### 3.1.12. Compound **15b**

Deacetylation of compound **14** (500 mg, 1.97 mmol) and subsequent alkylation with octyl bromide (820 μL, 4.72 mmol, 1.2 equiv./OH) in the presence of sodium hydride (315 mg, 7.88 mmol, 2 equiv./OH, 60% in mineral oil) were performed according to the synthesis of **15a**. The crude product was purified by flash column chromatography (hexane/ethylacetate 97:3→9:1) to yield **15b** (466 mg, 60%) as a colorless syrup. *R*_f_ = 0.24 (hexane/ethylacetate 95:5); ^1^H NMR (400 MHz, CDCl_3_) *δ* (ppm) 5.92–5.88 (m, 1H, H-3), 5.87–5.83 (m, 1H, allyl C*H*=CH_2_), 5.82–5.78 (m, 1H, H-2), 5.12–5.05 (m, 2H, allyl CH=C*H*_2_), 4.25–4.21 (m, 1H, H-1), 3.83–3.80 (m, 1H, H-4), 3.69–3.66 (m, 1H, H-5), 3.64–3.58 (m, 3H, octyl OC*H*_2_a, H-6a,b), 3.54–3.40 (m, 3H, octyl C*H*_2_b, octyl C*H*_2_), 2.47 (dt, *J* = 14.4 Hz, *J* = 7.3 Hz, 1H, allyl C*H*_2_a), 2.29 (dt, *J* = 13.9 Hz, *J* = 6.8 Hz, 1H, allyl C*H*_2_b), 1.62–1.53 (m, 4H, 2 × octyl C*H*_2_), 1.30–1.26 (m, 20H, 10 × octyl C*H*_2_), 0.89–0.86 (m, 6H, 2 × octyl C*H*_3_); ^13^C NMR (100 MHz, CDCl_3_) *δ* (ppm) 134.8 (1C, allyl C*H*=CH_2_), 130.7 (1C, C-2), 126.3 (1C, C-3), 117.2 (1C, allyl CH=C*H*_2_), 72.4 (1C, C-1), 71.8 (1C, octyl O*C*H_2_), 71.2 (1C, C-5), 70.6 (1C, C-4), 70.2 (1C, C-6), 69.4 (1C, octyl O*C*H_2_), 38.1 (1C, allyl *C*H_2_), 31.9, 30.2, 29.7, 29.6, 29.5, 29.4, 26.3, 22.8 (12C, 12 × octyl *C*H_2_), 14.2 (2C, 2 × octyl *C*H_3_); MALDI-TOF MS (Autoflex): *m*/*z* calcd for C_25_H_46_O_3_Na^+^: 417.3339 [M+Na]^+^; found: 417.3343.

#### 3.1.13. Compound **15c**

Deacetylation of compound **14** (500 mg, 1.97 mmol) and subsequent alkylation with hexyl bromide (664 μL, 4.72 mmol, 1.2 equiv./OH) in the presence of sodium hydride (315 mg, 7.88 mmol, 2 equiv./OH, 60% in mineral oil) were performed according to the synthesis of **15a**. The crude product was purified by flash column chromatography (hexane/ethylacetate 97:3→9:1) to yield **15c** (600 mg, 90%) as a colorless syrup. *R*_f_ = 0.28 (hexane/ethyl acetate 9:1); ^1^H NMR (400 MHz, CDCl_3_) *δ* (ppm) 5.92–5.89 (m, 1H, H-3), 5.88–5.82 (m, 1H, allyl C*H*=CH_2_), 5.82–5.79 (m, 1H, H-2), 5.12–5.05 (m, 2H, allyl CH=C*H*_2_), 4.23 (ddd, *J* = 8.1 Hz, *J* = 6.1 Hz, *J* = 2.2 Hz, 1H, H-1), 3.82 (dq, *J* = 7.4 Hz, *J* = 2.0 Hz, 1H, H-4), 3.68 (q, *J* = 3.7 Hz, *J* = 3.1 Hz, 1H, H-5), 3.64–3.59 (m, 3H, hexyl OC*H*_2_a, H-6a,b), 3.54–3.40 (m, 3H, hexyl C*H*_2_b, hexyl C*H*_2_), 2.51–2.44 (m, 1H, allyl C*H*_2_a), 2.32–2.25 (m, 1H, allyl C*H*_2_b), 1.63–1.53 (m, 4H, 2 × hexyl C*H*_2_), 1.39–1.21 (m, 12H, 6 × hexyl C*H*_2_), 0.91–0.87 (m, 6H, 2 × hexyl C*H*_3_); ^13^C NMR (100 MHz, CDCl_3_) *δ* (ppm) 134.8 (1C, allyl C*H*=CH_2_), 130.7 (1C, C-2), 126.3 (1C, C-3), 117.2 (1C, allyl CH=C*H*_2_), 72.4 (1C, C-1), 71.8 (1C, hexyl O*C*H_2_), 71.2 (1C, C-5), 70.6 (1C, C-4), 70.2 (1C, C-6), 69.4 (1C, hexyl O*C*H_2_), 38.1 (1C, allyl *C*H_2_), 31.8, 31.7, 30.1, 29.7, 26.0, 22.8 (8C, 8 × hexyl *C*H_2_), 14.2 (2C, 2 × hexyl *C*H_3_); MALDI-TOF MS (Autoflex): *m*/*z* calcd for C_21_H_38_O_3_Na^+^: 361.2713 [M+Na]^+^; found: 361.2691.

#### 3.1.14. Compound **17a**

Compound **15a** (100 mg, 0.22 mmol) and compound **16** (135 mg, 0.266 mmol, 1.2 equiv.) were dissolved in anhydrous toluene (5 mL), and 2,2-dimethoxy-2-phenylacetophenone (DPAP) (5.6 mg, 0.022 mmol, 0.1 equiv) was added. The reaction mixture was cooled to 0 °C and irradiated with UV light 4 times for 15 min, and DPAP (5.6 mg, 0.022 mmol, 0.1 equiv) was added before each irradiation cycle. Then, the solvent was evaporated and the crude product was purified by flash column chromatography (dichloromethane/acetone 94:6→9:1) to yield **17a** (81 mg, 38%) as a yellowish syrup. *R*_f_ = 0.28 (dichloromethane/acetone 9:1); ^1^H NMR (400 MHz, CDCl_3_) *δ* (ppm) 5.87 (d, *J* = 10.4 Hz, 1H, H-3), 5.77 (d, *J* = 10.9 Hz, 1H, H-2), 5.39 (d, *J* = 10.1 Hz, 1H, N*H*Ac), 5.33–5.31 (m, 2H, H-7′, H-8′), 4.86 (td, *J* = 11.5 Hz, *J* = 4.6 Hz, 1H, H-4′), 4.31 (dd, *J* = 12.4 Hz, *J* = 2.0 Hz, 1H, H-9′a), 4.17–4.15 (m, 1H, H-1), 4.11 (dd, *J* = 12.6 Hz, *J* = 4.6 Hz, 1H, H-9′b), 4.07–4.02 (m, 1H, H-5′), 3.85–3.81 (m, 2H, H-4, H-6′), 3.79 (s, 3H, COOC*H*_3_), 3.63–3.58 (m, 4H, H-5, H-6a,b, decyl OC*H*_2_a), 3.55–3.37 (m, 3H, decyl C*H*_2_b, decyl C*H*_2_), 2.76 (dd, *J* = 13.0 Hz, *J* = 7.1 Hz, 1H, SC*H*_2_a), 2.71 (dd, *J* = 12.8 Hz, *J* = 4.6 Hz, 1H, H-3′a), 2.58 (dt, *J* = 12.8 Hz, *J* = 7.3 Hz, 1H, SC*H*_2_b), 2.15, 2.14, 2.04, 2.03 (4 × s, 12H, 4 × Ac C*H*_3_), 1.97 (t, *J* = 12.3 Hz, 1H, H-3′b), 1.88 (s, 3H, NHAc C*H*_3_), 1.77–1.52 (m, 8H, 2 × C*H*_2_, 2 × decyl C*H*_2_), 1.28–1.24 (m, 28H, 14 × decyl C*H*_2_), 0.88 (t, *J* = 6.8 Hz, 6H, 2 × decyl C*H*_3_); ^13^C NMR (100 MHz, CDCl_3_) *δ* (ppm) 171.0, 170.7, 170.3, 170.2, 170.1 (5C, 5 × Ac C=O), 168.5 (1C, C-1′), 131.1 (1C, C-2), 126.1 (1C, C-3), 83.2 (1C, C-2′), 74.2 (1C, C-6′), 72.3 (1C, C-1), 71.8 (1C, decyl O*C*H_2_), 70.8 (1C, C-5), 70.5 (1C, C-4), 70.1 (1C, C-6), 69.8 (1C, C-4′), 69.4 (1C, decyl O*C*H_2_), 68.8 (1C, C-8′), 67.4 (1C, C-7′), 62.3 (1C, C-9′), 53.0 (1C, COO*C*H_3_), 49.4 (1C, C-5′), 38.1 (1C, C-3′), 32.0, 30.1, 29.7, 29.6, 29.4, 28.8, 26.3, 25.8, 22.8 (19C, 2 × *C*H_2_, S*C*H_2_, 16 × decyl *C*H_2_), 23.2 (1C, NHAc), 21.3, 20.9, 20.8 (4C, 4 × Ac *C*H_3_), 14.2 (2C, 2 × decyl *C*H_3_); MALDI-TOF MS (Autoflex): *m*/*z* calcd for C_49_H_83_NO_15_SNa^+^: 980.5376 [M+Na]^+^; found: 980.5368.

#### 3.1.15. Compound **17b**

Compound **15b** (95 mg, 0.24 mmol) was reacted with compound **16** (146 mg, 0.288 mmol, 1.2 equiv.) in the presence of DPAP (6.2 mg, 0.024 mmol, 0.1 equiv) according to the synthesis of **15a**. The crude product was purified by flash column chromatography (dichloromethane/methanol 99:1) to yield **17b** (125 mg, 57%) as a yellowish syrup. *R*_f_ = 0.46 (dichloromethane/acetone 8:2); ^1^H NMR (500 MHz, CDCl_3_) *δ* (ppm) 5.87 (dt, *J* = 10.4 Hz, *J* = 1.8 Hz, 1H, H-3), 5.77 (dt, *J* = 10.5 Hz, *J* = 2.0 Hz, 1H, H-2), 5.36 (dd, *J* = 5.8 Hz, *J* = 2.5 Hz, 1H, H-8′), 5.33 (td, *J* = 8.4 Hz, *J* = 7.8 Hz, *J* = 2.2 Hz, 1H, H-7′), 5.19 (d, *J* = 10.1 Hz, 1H, N*H*Ac), 4.86 (td, *J* = 11.5 Hz, *J* = 4.6 Hz, 1H, H-4′), 4.30 (dd, *J* = 12.5 Hz, *J* = 2.4 Hz, 1H, H-9′a), 4.16–4.15 (m, 1H, H-1), 4.11 (dd, *J* = 12.5 Hz, *J* = 5.0 Hz, 1H, H-9′b), 4.04 (q, *J* = 10.4 Hz, 1H, H-5′), 3.85–3.81 (m, 2H, H-4, H-6′), 3.80 (s, 3H, COOC*H*_3_), 3.63–3.58 (m, 4H, H-5, H-6a,b, octyl OC*H*_2_a), 3.54–3.38 (m, 3H, octyl C*H*_2_b, octyl C*H*_2_), 2.76 (dd, *J* = 13.1 Hz, *J* = 5.7 Hz, 1H, SC*H*_2_a), 2.71 (dd, *J* = 12.8 Hz, *J* = 4.7 Hz, 1H, H-3′a), 2.61–2.56 (m, 1H, SC*H*_2_b), 2.15, 2.14, 2.04, 2.03 (4 × s, 12H, 4 × Ac C*H*_3_), 1.97 (t, *J* = 12.3 Hz, 1H, H-3′b), 1.87 (s, 3H, NHAc C*H*_3_), 1.79–1.53 (m, 8H, 2 × C*H*_2_, 2 × octyl C*H*_2_), 1.34–1.26 (m, 20H, 10 × octyl C*H*_2_), 0.89–0.87 (m, 6H, 2 × octyl C*H*_3_); ^13^C NMR (125 MHz, CDCl_3_) *δ* (ppm) 170.9, 170.6, 170.1, 169.9 (5C, 5 × Ac C=O), 168.5 (1C, C-1′), 131.0 (1C, C-2), 126.0 (1C, C-3), 83.1 (1C, C-2′), 74.1 (1C, C-6′), 72.2 (1C, C-1), 71.7 (1C, octyl O*C*H_2_), 70.8 (1C, C-5), 70.5 (1C, C-4), 70.1 (1C, C-6), 69.7 (1C, C-4′), 69.3 (1C, octyl O*C*H_2_), 68.6 (1C, C-8′), 67.3 (1C, C-7′), 62.2 (1C, C-9′), 52.9 (1C, COO*C*H_3_), 49.4 (1C, C-5′), 38.1 (1C, C-3′), 32.0, 31.8, 30.1, 29.6, 29.5, 29.3, 28.7, 26.2, 25.8, 22.7 (15C, 2 × *C*H_2_, S*C*H_2_, 12 × decyl *C*H_2_), 23.2 (1C, NHAc), 21.2, 20.8, 20.7 (4C, 4 × Ac *C*H_3_), 14.1 (2C, 2 × decyl *C*H_3_); MALDI-TOF MS (Autoflex): *m*/*z* calcd for C_45_H_75_NO_15_SNa^+^: 924.4750 [M+Na]^+^; found: 924.4753.

#### 3.1.16. Compound **17c**

Compound **15c** (97 mg 0.25 mmol) was reacted with compound **16** (152 mg, 0.30 mmol, 1.2 equiv.) in the presence of DPAP (6.5 mg, 0.025 mmol, 0.1 equiv) according to the synthesis of **15a**. The crude product was purified by flash column chromatography (dichloromethane/methanol 99:1) to yield **17c** (85 mg, 35%) as a yellowish syrup. *R*_f_ = 0.35 (dichloromethane/acetone 8:2); ^1^H NMR (400 MHz, CDCl_3_) *δ* (ppm) 5.87 (d, *J* = 10.4 Hz, 1H, H-3), 5.77 (d, *J* = 10.5 Hz, 1H, H-2), 5.38–5.31 (m, 2H, H-7′, H-8′), 5.28 (d, *J* = 10.1 Hz, 1H, N*H*Ac), 4.86 (td, *J* = 11.3 Hz, *J* = 4.6 Hz, 1H, H-4′), 4.30 (dd, *J* = 12.4 Hz, *J* = 1.9 Hz, 1H, H-9′a), 4.16–4.15 (m, 1H, H-1), 4.11 (dd, *J* = 12.5 Hz, *J* = 4.6 Hz, 1H, H-9′b), 4.09–4.01 (m, 1H, H-5′), 3.83–3.81 (m, 2H, H-4, H-6′), 3.80 (s, 3H, COOC*H*_3_), 3.63–3.58 (m, 4H, H-5, H-6a,b, hexyl OC*H*_2_a), 3.55–3.38 (m, 3H, hexyl C*H*_2_b, hexyl C*H*_2_), 2.76 (dd, *J* = 12.7 Hz, *J* = 6.7 Hz, 1H, SC*H*_2_a), 2.71 (dd, *J* = 12.8 Hz, *J* = 4.5 Hz, 1H, H-3′a), 2.58 (dt, *J* = 12.9 Hz, *J* = 7.2 Hz, 1H, SC*H*_2_b), 2.15, 2.14, 2.04, 2.03 (4 × s, 12H, 4 × Ac C*H*_3_), 1.97 (t, *J* = 12.3 Hz, 1H, H-3′b), 1.88 (s, 3H, NHAc C*H*_3_), 1.77–1.52 (m, 8H, 2 × C*H*_2_, 2 × hexyl C*H*_2_), 1.34–1.24 (m, 12H, 6 × hexyl C*H*_2_), 0.90–0.87 (m, 6H, 2 × hexyl C*H*_3_); ^13^C NMR (100 MHz, CDCl_3_) *δ* (ppm) 171.1, 170.7, 170.3, 170.2, 170.1 (5C, 5 × Ac C=O), 168.6 (1C, C-1′), 131.1 (1C, C-2), 126.1 (1C, C-3), 83.2 (1C, C-2′), 74.2 (1C, C-6′), 72.3 (1C, C-1), 71.8 (1C, hexyl O*C*H_2_), 70.8 (1C, C-5), 70.6 (1C, C-4), 70.2 (1C, C-6), 69.8 (1C, C-4′), 69.4 (1C, hexyl O*C*H_2_), 68.7 (1C, C-8′), 67.4 (1C, C-7′), 62.3 (1C, C-9′), 53.1 (1C, COO*C*H_3_), 49.5 (1C, C-5′), 38.2 (1C, C-3′), 32.1, 31.8, 30.1, 29.7, 28.8, 26.0, 25.9, 22.7 (11C, 2 × *C*H_2_, S*C*H_2_, 8 × decyl *C*H_2_), 23.3 (1C, NHAc), 21.3, 21.0, 20.9 (4C, 4 × Ac *C*H_3_), 14.2 (2C, 2 × hexyl *C*H_3_); MALDI-TOF MS (Autoflex): *m*/*z* calcd for C_41_H_67_NO_15_SNa^+^: 868.4124 [M+Na]^+^; found: 868.4126.

#### 3.1.17. Compound **18a**

Compound **17a** (75 mg, 0.078 mmol) was dissolved in anhydrous methanol (10 mL), and little pieces of sodium were added to make the pH basic (pH ~ 11). After 1 h of stirring, Amberlite IR-50 H^+^ ion exchange resin was added, and the pH was adjusted to neutral. Then, the reaction mixture was filtered, and the solvent was evaporated. 

The crude product of the previous step was dissolved in 1,4-dioxane (9 mL) and water (1 mL), and the reaction mixture was cooled to 0 °C. Then, an aqueous solution of potassium hydroxide (0.2 M, 1.95 mL, 0.39 mmol, 5 equiv.) was added, and the reaction mixture was stirred at room temperature for 4 h; then, Amberlite IR-50 H^+^ ion exchange resin was added, and the pH was adjusted to neutral. The reaction mixture was filtered, and the solvent was evaporated in a vacuum. The residue was purified by flash column chromatography (acetonitrile/water 95:5→9:1) to yield **18a** (44.3 mg, 73%) as a yellowish syrup. *R*_f_ = 0.45 (acetonitrile/water 8:2); ^1^H NMR (400 MHz, MeOD) *δ* (ppm) 5.89–5.82 (m, 2H, H-2, H-3), 4.14–4.10 (m, 1H, H-1), 3.85–3.67 (m, 6H, H-4, H-4′, H-5′, H-8′, H-9′a,b), 3.64–3.59 (m, 4H, H-5, H-6a,b, H-7′), 3.55–3.40 (m, 5H, H-6′, 2 × decyl OC*H*_2_), 2.89–2.82 (m, 2H, H-3′a, SC*H*_2_a), 2.74–2.67 (m, 1H, SC*H*_2_b), 2.01 (s, 3H, NHAc C*H*_3_), 1.82–1.52 (m, 9H, H-3′b, 2 × C*H*_2_, 2 × decyl C*H*_2_), 1.32–1.28 (m, 28H, 14 × decyl C*H*_2_), 0.90 (t, *J* = 6.6 Hz, 6H, 2 × decyl C*H*_3_); ^13^C NMR (100 MHz, MeOD) *δ* (ppm) 175.5, 175.1 (2C, C-1′, NAc C=O), 132.5 (1C, C-2), 126.4 (1C, C-3), 87.2 (1C, C-2′), 76.6 (1C, C-6′), 73.5 (1C, C-1), 73.0 (1C, C-8′), 72.6 (1C, decyl O*C*H_2_), 72.3 (1C, C-5), 71.8 (1C, C-4), 71.1 (1C, C-6), 70.2 (1C, C-7′), 70.0 (1C, decyl O*C*H_2_), 69.6 (1C, C-4′), 64.3 (1C, C-9′), 54.0 (1C, C-5′), 43.2 (1C, C-3′), 33.4, 33.1, 31.1, 30.8, 30.7, 30.6, 30.5, 30.4, 27.7, 27.3, 23.7 (19C, 2 × *C*H_2_, S*C*H_2_, 16 × decyl *C*H_2_), 22.6 (1C, NHAc), 14.5 (2C, 2 × decyl *C*H_3_); MALDI-TOF MS (Autoflex): *m*/*z* calcd for C_40_H_73_NO_11_SNa^+^: 798.4797 [M+Na]^+^; found: 798.4800.

#### 3.1.18. Compound **18b**

Compound **17b** (124 mg, 0.135 mmol) was deacetylated; then, methyl ester hydrolysis was performed with potassium hydroxide solution (0.2 M, 3.38 mL, 0.675 mmol, 5 equiv.) according to the procedure for **18a**. The crude product was purified by flash column chromatography (acetonitrile/water 95:5→9:1) to yield **18b** (37 mg, 39%) as a yellowish syrup. *R*_f_ = 0.47 (acetonitrile/water 9:1); ^1^H NMR (500 MHz, MeOD) *δ* (ppm) 5.89–5.82 (m, 2H, H-2, H-3), 4.12–4.11 (m, 1H, H-1), 3.84–3.61 (m, 7H, H-4, H-4′, H-5′, H-5, H-8′, H-9′a,b), 3.60–3.58 (m, 2H, H-6a,b), 3.54–3.41 (m, 6H, H-6′, H-7′, 2 × octyl OC*H*_2_), 2.88–2.83 (m, 2H, H-3′a, SC*H*_2_a), 2.74–2.68 (m, 1H, SC*H*_2_b), 2.01 (s, 3H, NHAc C*H*_3_), 1.81–1.52 (m, 9H, H-3′b, 2 × C*H*_2_, 2 × octyl C*H*_2_), 1.37–1.25 (m, 20H, 10 × octyl C*H*_2_), 0.90 (t, *J* = 6.8 Hz, 6H, 2 × octyl C*H*_3_); ^13^C NMR (125 MHz, MeOD) *δ* (ppm) 175.5 (1C, NAc C=O), 175.1 (1C, C-1′), 132.6 (1C, C-2), 126.4 (1C, C-3), 87.2 (1C, C-2′), 76.6 (1C, C-6′), 73.4 (1C, C-1), 73.0 (1C, C-8′), 72.6 (1C, octyl O*C*H_2_), 72.3 (1C, C-5), 71.8 (1C, C-4), 71.1 (1C, C-6), 70.2 (1C, C-7′), 70.0 (1C, octyl O*C*H_2_), 69.6 (1C, C-4′), 64.3 (1C, C-9′), 54.0 (1C, C-5′), 43.2 (1C, C-3′), 33.4 (1C, *C*H_2_), 33.0, 31.1, 30.7, 30.6, 30.5 (9C, S*C*H_2_, 8 × octyl *C*H_2_), 27.5 (1C, *C*H_2_), 27.3, 23.7 (4C, 4 × octyl *C*H_2_), 22.6 (1C, NHAc), 14.5 (2C, 2 × octyl *C*H_3_); MALDI-TOF MS (Autoflex): *m*/*z* calcd for C_36_H_65_NO_11_SNa^+^: 742.417 [M+Na]^+^; found: 742.406.

#### 3.1.19. Compound **18c**

Compound **17c** (78 mg, 0.092 mmol) was deacetylated; then, methyl ester hydrolysis was performed with potassium hydroxide solution (0.2 M, 2.3 mL, 0.46 mmol, 5 equiv.) according to the procedure for **18a**. The crude product was purified by flash column chromatography (acetonitrile/water 95:5→9:1) to yield **18c** (25.3 mg, 41%) as a yellowish syrup. *R*_f_ = 0.38 (acetonitrile/water 8:2); ^1^H NMR (400 MHz, MeOD) *δ* (ppm) 5.89–5.82 (m, 2H, H-2, H-3), 4.14–4.10 (m, 1H, H-1), 3.82–3.67 (m, 6H, H-4, H-4′, H-5′, H-8′, H-9′a,b), 3.65–3.59 (m, 4H, H-5, H-6a,b, H-7′), 3.54–3.39 (m, 5H, H-6′, 2 × hexyl OC*H*_2_), 2.88–2.85 (m, 2H, H-3′a, SC*H*_2_a), 2.72–2.70 (m, 1H, SC*H*_2_b), 2.00 (s, 3H, NHAc C*H*_3_), 1.83–1.51 (m, 9H, H-3′b, 2 × C*H*_2_, 2 × hexyl C*H*_2_), 1.40–1.29 (m, 12H, 6 × hexyl C*H*_2_), 0.91 (t, *J* = 6.7 Hz, 6H, 2 × hexyl C*H*_3_); ^13^C NMR (100 MHz, MeOD) *δ* (ppm) 175.5 (2C, C-1′, NAc C=O), 132.6 (1C, C-2), 126.4 (1C, C-3), 86.2 (1C, C-2′), 76.6 (1C, C-6′), 73.4 (1C, C-1), 73.0 (1C, C-8′), 72.6 (1C, hexyl O*C*H_2_), 72.3 (1C, C-5), 71.8 (1C, C-4), 71.2 (1C, C-6), 70.3 (1C, C-7′), 70.0 (1C, decyl O*C*H_2_), 69.7 (1C, C-4′), 64.4 (1C, C-9′), 54.0 (1C, C-5′), 43.2 (1C, C-3′), 33.4 (1C, *C*H_2_), 32.9, 32.8, 31.8, 30.7 (4C, 4 × hexyl *C*H_2_), 30.5 (1C, S*C*H_2_), 27.5 (1C, *C*H_2_), 27.0, 23.7 (4C, 4 × hexyl *C*H_2_), 22.6 (1C, NHAc), 14.4 (2C, 2 × hexyl *C*H_3_); MALDI-TOF MS (Autoflex): *m*/*z* calcd for C_32_H_56_NO_11_SNa_2_^+^: 708.3364 [M-H+2Na]^+^; found: 708.3367.

#### 3.1.20. Compound **20**

Tri-*O*-acetyl-D-glucal (**13**, 1.8 g, 6.6 mmol) and monoallyl tetraethylene glycol (**19**, 2.319 g, 9.9 mmol, 1.5 equiv.) were dissolved in anhydrous dichloromethane (15 mL). A 4Å molecular sieve (1.8 g) was added, and the flask was plugged with a drying tube filled with anhydrous CaCl_2_. The mixture was stirred at room temperature for 15 min; then, boron trifluoride diethyl etherate (405 µL, 3.3 mmol, 0.5 equiv.) was added. After 60 min, a saturated aqueous solution of NaHCO_3_ (5 mL) was added, and the reaction mixture was stirred for 15 min. Then, the solvent was evaporated in a vacuum, and the residue was dissolved in dichloromethane (500 mL). This solution was washed three times with aqueous saturated NaHCO_3_ solution (3 × 50 mL) and twice with water (2 × 50 mL). The organic phase was dried over Na_2_SO_4_, then filtered, and the solvent was evaporated in a vacuum. The residue was purified by flash column chromatography (hexane/acetone 8:2) to yield **20** (1.855 g, 90%) as a colorless syrup. *R*_f_ = 0.24 (hexane/acetone 7:3); ^1^H NMR (400 MHz, CDCl_3_) *δ* (ppm) 5.96–5.89 (m, 1H, C*H*=CH_2_), 5.87 (s, 2H, H-2, H-3), 5.32 (d, *J* = 9.6 Hz, 1H, H-4), 5.27 (dd, *J* = 17.2 Hz, *J* = 1.6 Hz, 1H, allyl CH=C*H*_2_a), 5.19–5.16 (m, 1H, allyl CH=C*H*_2_b), 5.08 (s, 1H, H-1), 4.26 (dd, *J* = 12.1 Hz, *J* = 5.1 Hz, 1H, H-6a), 4.17 (dd, *J* = 12.1 Hz, *J* = 2.3 Hz, 1H, H-6b), 4.12 (ddd, *J* = 9.3 Hz, *J* = 5.1 Hz, *J* = 2.2 Hz, 1H, H-5), 4.03–4.02 (m, 2H, allyl C*H*_2_), 3.91–3.59 (m, 16H, 8 × TEG OC*H*_2_), 2.10, 2.09 (2 × s, 6H, 2 × Ac C*H*_3_); ^13^C NMR (100 MHz, CDCl_3_) *δ* (ppm) 170.8, 170.3 (2C, 2 × Ac C=O), 134.8 (1C, allyl C*H*=CH_2_), 129.2, 127.8 (2C, C-2, C-3), 117.1 (1C, allyl CH=C*H*_2_), 94.7 (1C, C-1), 72.8, 70.6, 70.5, 69.5, 67.9 (9C, allyl *C*H_2_, 8 × TEG O*C*H_2_), 66.9 (1C, C-5), 65.3 (1C, C-4), 63.0 (1C, C-6), 21.0, 20.9 (2C, 2 × Ac *C*H_3_); MALDI-TOF MS (Autoflex): *m*/*z* calcd for C_21_H_34_O_10_Na^+^: 469.2044 [M+Na]^+^; found: 469.2035.

#### 3.1.21. Compound **21a**

Compound **20** (500 mg, 1.12 mmol) was dissolved in anhydrous methanol (20 mL), and small pieces of sodium were added to make the solution alkaline (pH~11). The reaction mixture was stirred for 1 h; then, Serdolit Red H^+^ ion exchange resin was added to make the pH neutral. Thereafter, the reaction mixture was filtered, and the solvent was evaporated.

In the second step, sodium hydride (0.179 g, 4.48 mmol, 2 equiv./OH, 60% in mineral oil) was washed with hexane and dried under argon for 30 min. The crude product of the previous step was dissolved in anhydrous *N*,*N*-dimethylformamide (5 mL), and this solution was added to the washed sodium hydride. The mixture was stirred under an argon atmosphere for 30 min; then, octyl bromide (0.585 mL, 3.36 mmol, 3 equiv.) was added. The reaction mixture was stirred at room temperature overnight; then, methanol (1 mL) and water (1 mL) were added successively, and the reaction mixture was stirred for 2 × 15 min. The solvent was evaporated, and the residue was purified by flash column chromatography (hexane/acetone 9:1) to yield **21a** (240 mg, 37%) as a colorless syrup. *R*_f_ = 0.6 (hexane/acetone 7:3); ^1^H NMR (400 MHz, CDCl_3_) *δ* (ppm) 6.03 (d, *J* = 10.3 Hz, 1H, H-3), 5.91 (ddt, *J* = 16.1 Hz, *J* = 10.7 Hz, *J* = 5.7 Hz, 1H, C*H*=CH_2_), 5.75 (dt, *J* = 10.3 Hz, *J* = 2.0 Hz, 1H, H-2), 5.29–5.25 (m, 1H, allyl CH=C*H*_2_a), 5.19–5.15 (m, 1H, allyl CH=C*H*_2_b), 5.03 (s, 1H, H-1), 4.03–4.02 (m, 2H, allyl C*H*_2_), 3.98–3.96 (m, 1H, H-4), 3.91–3.87 (m, 1H, H-6a), 3.85–3.82 (m, 1H, H-5), 3.71–3.60 (m, 17H, H-6b, 8 × TEG OC*H*_2_), 3.59–3.37 (m, 4H, 2 × octyl OC*H*_2_), 1.63–1.51 (m, 4H, 2 × octyl C*H*_2_), 1.41–1.27 (m, 20H, 10 × octyl C*H*_2_), 0.89–0.86 (m, 6H, 2 × octyl C*H*_3_); ^13^C NMR (100 MHz, CDCl_3_) *δ* (ppm) 134.8 (1C, allyl C*H*=CH_2_), 131.2 (1C, C-3), 126.2 (1C, C-2), 117.1 (1C, allyl CH=C*H*_2_), 94.9 (1C, C-1), 72.3, 71.8, 70.7, 70.6, 70.5, 69.7, 69.5 (12C, C-4, allyl *C*H_2_, 2 × octyl O*C*H_2_, 8 × TEG O*C*H_2_), 69.3 (1C, C-5), 67.6 (1C, C-6), 31.9, 30.1, 29.7, 29.5, 29.4, 26.2, 22.7 (12C, 12 × octyl *C*H_2_), 14.2 (2C, 2 × octyl *C*H_3_); MALDI-TOF MS (Autoflex): *m*/*z* calcd for C_33_H_62_O_8_Na^+^: 609.4337 [M+Na]^+^; found: 609.4340.

#### 3.1.22. Compound **21b**

Compound **20** (500 mg, 1.12 mmol) was deacetylated, then alkylated with hexyl bromide (0.473 mL, 3.36 mmol, 3 equiv.) in the presence of sodium hydride (0.179 g, 4.48 mmol, 2 equiv./OH, 60% in mineral oil) according to the procedure for **21a**. The crude product was purified by flash column chromatography (hexane/acetone 9:1) to yield **21b** (303 mg, 41%) as a colorless syrup. *R*_f_ = 0.59 (hexane/acetone 6:4); ^1^H NMR (400 MHz, CDCl_3_) *δ* (ppm) 6.03 (d, *J* = 10.2 Hz, 1H, H-3), 5.96–5.82 (m, 1H, C*H*=CH_2_), 5.77–5.73 (m, 1H, H-2), 5.27 (dq, *J* = 17.2 Hz, *J* = 1.6 Hz, 1H, allyl CH=C*H*_2_a), 5.19–5.14 (m, 1H, allyl CH=C*H*_2_b), 5.04 (s, 1H, H-1), 4.02 (dt, *J* = 5.7 Hz, *J* = 1.4 Hz, 2H, allyl C*H*_2_), 3.98–3.96 (m, 1H, H-4), 3.91–3.88 (m, 1H, H-6a), 3.86–3.81 (m, 1H, H-5), 3.72–3.60 (m, 17H, H-6b, 8 × TEG OC*H*_2_), 3.59–3.37 (m, 4H, 2 × hexyl C*H*_2_), 1.65–1.51 (m, 4H, 2 × hexyl C*H*_2_), 1.37–1.26 (m, 12H, 6 × hexyl C*H*_2_), 0.91–0.87 (m, 6H, 2 × hexyl C*H*_3_); ^13^C NMR (100 MHz, CDCl_3_) *δ* (ppm) 134.8 (1C, allyl C*H*=CH_2_), 131.2 (1C, C-3), 126.2 (1C, C-2), 117.1 (1C, allyl CH=C*H*_2_), 94.9 (1C, C-1), 72.3, 71.8, 70.7, 70.6, 70.5, 69.7, 69.5 (12C, C-4, allyl *C*H_2_, 2 × hexyl O*C*H_2_, 8 × TEG O*C*H_2_), 69.2 (1C, C-5), 67.6 (1C, C-6), 31.8, 31.7, 30.1, 29.6, 25.9, 22.7 (8C, 8 × hexyl *C*H_2_), 14.1 (2C, 2 × hexyl *C*H_3_); MALDI-TOF MS (Autoflex): *m*/*z* calcd for C_29_H_54_O_8_Na^+^: 553.3711 [M+Na]^+^; found: 553.3692.

#### 3.1.23. Compound **22a**

Compound **21a** (183 mg, 0.312 mmol) and compound **16** (174 mg, 0.343 mmol, 1.1 equiv.) were dissolved in anhydrous toluene (3 mL), and 2,2-dimethoxy-2-phenylacetophenone (DPAP) (8 mg, 0.0312 mmol, 0.1 equiv.) was added. The reaction mixture was cooled to 0 °C and irradiated with UV light for 4 × 15 min; DPAP (8 mg, 0.0312 mmol, 0.1 equiv.) was added before each irradiation cycle. Then, the solvent was evaporated in a vacuum, and the residue was purified by flash column chromatography (hexane/acetone 9:1) to yield **22a** (72 mg, 21%) as a colorless syrup. It was used for further conversion without NMR characterization. *R*_f_ = 0.38 (hexane/acetone 6:4); MALDI-TOF MS (Autoflex): *m*/*z* calcd for C_53_H_91_N_1_O_20_SNa^+^: 1116.5747 [M+Na]^+^; found: 1116.5711.

#### 3.1.24. Compound **22b**

Compound **21b** (150 mg, 0.283 mmol) and compound **16** (172 mg, 0.339 mmol, 1.2 equiv.) were reacted in the presence of DPAP (7 mg, 0.0283 mmol, 0.1 equiv.) according to the procedure applied for the synthesis of **22a**. The crude product was purified by flash column chromatography (hexane/acetone 8:2→6:4) to yield **22b** (170 mg, 51%) as a colorless syrup. It was used for further conversion without NMR characterization. *R*_f_ = 0.33 (dichloromethane/acetone 8:2); MALDI-TOF MS (Autoflex): *m*/*z* calcd for C_49_H_83_N_1_O_20_SNa^+^: 1060.5121 [M+Na]^+^; found: 1060.5128.

#### 3.1.25. Compound **23a**

Compound **22a** (71 mg, 0.065 mmol) was dissolved in anhydrous methanol (10 mL), and little pieces of sodium were added to make the pH basic (pH~11). After 1 h of stirring, Amberlite IR-50 H^+^ ion exchange resin was added, and the pH was adjusted to neutral. Then, the reaction mixture was filtered, and the solvent was evaporated.

The residue was dissolved in 1,4-dioxane (9 mL) and water (1 mL), and the reaction mixture was cooled to 0 °C. Then, an aqueous solution of potassium hydroxide (0.2 M, 1.625 mL, 0.325 mmol, 5 equiv.) was added, and the reaction mixture was stirred at room temperature for 4 h; then, Amberlite IR-50 H^+^ ion exchange resin was added, and the pH was adjusted to neutral. The reaction mixture was filtered; the solvent was evaporated in a vacuum, and the residue was purified by flash column chromatography (acetonitrile/water 9:1) to yield **23a** (46.4 mg, 78%) as a colorless syrup. *R*_f_ = 0.43 (acetonitrile/water 8:2); ^1^H NMR (400 MHz, CDCl_3_) *δ* (ppm) 5.99 (d, *J* = 10.3 Hz, 1H, H-3), 5.69 (d, *J* = 10.3 Hz, 1H, H-2), 4.93 (s, 1H, H-1), 3.81–3.68 (m, 4H, H-4, H-5, H-6a, H-9′a), 3.66–3.30 (m, 29H, H-6b, H-4′, H-5′, H-6′, H-7′, H-8′, H-9′b, OC*H*_2_, 2 × octyl OC*H*_2_, 8 × TEG OC*H*_2_), 2.79–2.72 (m, 2H, H-3′a, SC*H*_2_a), 2.66–2.59 (m, 1H, SC*H*_2_b), 1.92 (s, 3H, NHAc), 1.87–1.75 (m, 2H, C*H*_2_), 1.57–1.54 (m, 1H, H-3′b), 1.51–1.43 (m, 4H, 2 × octyl C*H*_2_), 1.21–1.12 (m, 20H, 10 × octyl C*H*_2_), 0.82–0.79 (m, 6H, 2 × octyl C*H*_3_); ^13^C NMR (100 MHz, CDCl_3_) *δ* (ppm) 175.5 (2C, Ac C=O, C-1′), 131.9 (1C, C-3), 127.3 (1C, C-2), 96.0 (1C, C-1), 87.4 (1C, C-2′), 76.6 (1C, C-6′), 73.0 (1C, C-8′), 72.6 (2C, 2 × octyl O*C*H_2_), 72.1 (1C, C-4), 71.4, 71.3, 71.2, 71.1, 70.9, 70.3 (9C, O*C*H_2_, 8 × TEG O*C*H_2_), 70.7 (2C, C-5, C-7′), 69.6 (1C, C-4′), 68.5 (1C, C-6), 64.4 (1C, C-9′), 54.0 (1C, C-5′), 43.2 (1C, C-3′), 33.0, 31.1, 31.0, 30.7, 30.6, 30.5, 27.4, 27.3, 23.7 (14C, *C*H_2_, S*C*H_2_, 12 × octyl *C*H_2_), 22.6 (1C, NHAc), 14.5 (2C, 2 × octyl *C*H_3_); MALDI-TOF MS (Autoflex): *m*/*z* calcd for C_44_H_80_N_1_O_16_SNa_2_^+^: 956.4988 [M-H+2Na]^+^; found: 956.4993.

#### 3.1.26. Compound **23b**

Compound **22b** (170 mg, 0.164 mmol) was deacetylated, and then, the methyl ester was hydrolyzed with an aqueous solution of potassium hydroxide (0.2 M, 4.1 mL, 0.82 mmol, 5 equiv.) according to the procedure applied for the synthesis of **23a**. The crude product was purified by flash column chromatography (acetonitrile/water 9:1) to yield **23b** (61 mg, 44%) as a colorless syrup. *R*_f_ = 0.56 (acetonitrile/water 8:2); ^1^H NMR (400 MHz, CDCl_3_) *δ* (ppm) 6.08 (d, *J* = 10.3 Hz, 1H, H-3), 5.77 (dt, *J* = 10.3 Hz, *J* = 2.1 Hz, 1H, H-2), 5.02 (s, 1H, H-1), 3.90–3.83 (m, 4H, H-4, H-5, H-6a, H-9′a), 3.78–3.39 (m, 29H, H-6b, H-4′, H-5′, H-6′, H-7′, H-8′, H-9′b, OC*H*_2_, 2 × hexyl OC*H*_2_, 8 × TEG OC*H*_2_), 2.89–2.81 (m, 2H, H-3′a, SC*H*_2_a), 2.76–2.71 (m, 1H, SC*H*_2_b), 2.02 (s, 3H, NHAc), 1.94–1.86 (m, 2H, C*H*_2_), 1.66–1.63 (m, 1H, H-3′b), 1.60–1.52 (m, 4H, 2 × hexyl C*H*_2_), 1.39–1.28 (m, 12H, 6 × hexyl C*H*_2_), 0.93–0.89 (m, 6H, 2 × hexyl C*H*_3_); ^13^C NMR (100 MHz, CDCl_3_) *δ* (ppm) 175.5, 175.0 (2C, Ac C=O, C-1′), 131.9 (1C, C-3), 127.3 (1C, C-2), 96.0 (1C, C-1), 87.3 (1C, C-2′), 76.6 (1C, C-6′), 73.0 (1C, C-8′), 72.6 (2C, 2 × hexyl O*C*H_2_), 72.1 (1C, C-4), 71.4, 71.3, 71.2, 71.1, 71.0, 70.9, 70.7, 70.3 (9C, O*C*H_2_, 8 × TEG O*C*H_2_), 70.7 (2C, C-5, C-7′), 69.5 (1C, C-4′), 68.5 (1C, C-6), 64.4 (1C, C-9′), 54.0 (1C, C-5′), 43.2 (1C, C-3′), 32.8, 32.7, 31.1, 31.0, 30.7, 27.4, 26.9, 23.7 (10C, *C*H_2_, S*C*H_2_, 8 × hexyl *C*H_2_), 22.7 (1C, NHAc), 14.4 (2C, 2 × hexyl *C*H_3_); ESI-QTOF MS: *m*/*z* calcd for C_40_H_72_NO_16_S^−^: 854.457 [M-H]^−^; found: 854.455.

### 3.2. Antiviral Evaluation

The process of CPE reduction assay for the influenza virus was described previously [44]. The virus strains were A/Virginia/ATCC3/2009, A/PR/8 (A/H1N1), A/Victoria/361/11, A/HK/7/87 (A/H3N2), and B/Ned/537/05 (IBV). On day 0, Madin-Darby canine kidney (MDCK) cells in 96-well plates were infected with influenza virus at a multiplicity of infection (MOI) of 0.0004 plaque forming units (PFU) per cell, and that was incubated at 35 °C for 3 days. After the third day, virus-induced CPE and compound cytotoxicity were scored by microscopy, and the data were confirmed by formazan-based MTS cell viability assay (CellTiter 96^®^ AQueous One Solution Cell Proliferation Assay from Promega, Madison, WI, USA). Antiviral activity was expressed as a compound concentration that resulted in 50% inhibition of virus-induced cytopathic effect (CPE) (EC_50_). Compound cytotoxicity was expressed as the concentration of the compound causing minimal changes in cell morphology (MCC) and 50% cytotoxic concentration (CC_50_) based on the MTS assay.

### 3.3. Surface Plasmon Resonance (SPR) Experiments

SPR experiments were conducted using a BIAcore S200 instrument (Cytiva, Marlborough, MA, USA) employing a CM5 chip with a carboxymethyldextran surface (Cytiva, USA). Hemagglutinins (ThermoFisher Scientific, Invitrogen, Waltham, MA, USA) from influenza type H3N2 (A/Aichi/2/1968) and H1N1 (A/Puerto Rico/8/34), as well as neuraminidase (Invitrogen, USA) from H1N1 (A/California/04/2009), were immobilized on the chip surface following the standard amine coupling procedure outlined in the manufacturer’s instructions. This immobilization process was performed at a flow rate of 10 μL/min using PBST as the running buffer. The chip surface was initially activated using an *N*-ethyl-*N*-(3-dimethylaminopropyl)carbodiimide/*N*-hydroxysuccinimide solution. Subsequently, 50 μg/mL solutions of individual proteins in 10 mM sodium acetate, pH 5, were injected into channels 2, 3, and 4, respectively. To block any unreacted groups, all channels were treated with 1 M ethanolamine, resulting in a final response of 4500 RU for HAs and 2700 RU for neuraminidase. Channel 1 was activated and blocked in the same manner, without the injection of any protein, serving as a blank.

Tested compounds in a PBST working buffer (20 mM Na-phosphate, 150 mM NaCl, pH 7.4, 0.005% Tween 20, 5% DMSO) were injected into the chip at a flow rate of 30 μL/min at 25 °C. The injection duration was 3 min, followed by a 10 min buffer flow to facilitate spontaneous dissociation. Each compound was injected as a two-fold dilution series within a concentration range of 0.78–800 μM (2.44–2500 μM for sialic acid). After each compound concentration series, five buffer injections were performed to ensure complete dissociation. To determine the apparent K_D_ toward the immobilized proteins, the steady-state responses of the blank-subtracted curves from each replicate were fitted using a single-site binding model.

In experiments conducted in a DMSO-free buffer (20 mM Na-phosphate, 150 mM NaCl, pH 7.4, 0.005% Tween 20), each replicate was followed by a two-fold dilution series of DMSO, matching the DMSO concentration in the compound solutions. In this case, the steady-state response of the blank-subtracted curve was corrected by the corresponding blank-subtracted DMSO steady-state response. The final values from each replicate were fitted using a single-site binding model using Origin 7 (OriginLab Corporation, Northampton, MA, USA).

For all experiments, the final K_D_(app) and R_max_ values, along with their standard deviations, were obtained through statistical analysis based on three replicates.

### 3.4. Dynamic Light Scattering Investigations

The hydrodynamic size and size distributions of compounds **12a**, **12b,** and **12c** were determined by dynamic light scattering (DLS). DLS measurements were performed using a Zetasizer Nano ZS (Malvern Instruments, Malvern, UK) equipped with a He-Ne laser (633 nm) at 25 °C and at a detector position of 175°. The Z-average size was calculated by cumulants analysis. The distribution by intensity of particle sizes was determined by multiple exponential fit. Solutions with a concentration of 1 mg/mL were prepared in water/DMSO 9:1 mixture from the examined compounds, and these were diluted with water for solutions with a concetration of 0.1 mg/mL.

## 4. Conclusions

Three similar series of amphiphilic neuraminic acid derivatives equipped with lipophilic side chains of different lengths were synthesized. The series differ in their linkers between the lipophilic chains and the sialic acid moiety. The sialic acid moiety is connected to the linker by an enzymatically stable thioglycosidic bond, and all of the derivatives contain double lipophilic tails (*n*-butyl, *n*-hexyl *n*-octyl, or *n*-decyl chains). Our goal was to study the influence of the linker and the length of the lipophilic chain on the antiviral activity. According to the scientific literature and our results [45], the overall lipophilicity and the length of the lipophilic side chain are momentous in terms of the antiviral effect. Mehta et al. reported on a structure–activity relationship of lipophilic side chain containing galactonojirimycin derivatives against hepatitis B virus, where the antiviral activity of the compounds decreased sharply with side chains of fewer than eight carbons [46], while Uozaki et al. showed that the antiviral activity of alkyl gallates increased with the length of the alkyl chains, up to 12 carbons, and octyl group proved to be the most ideal since octyl gallate showed a marked antiviral effect with a relatively moderate cytotoxicity [47]. In order to quantify the lipophilicity of our new compounds, the cLogP values were calculated for the synthesized derivatives (Table 4). 

According to the results of anti-influenza tests, only two octyl derivatives (**12b** and **18b**) have a moderate effect against influenza A H1N1 and H3N2 viruses, while the octyl derivative **23a**, as well as all of the other derivatives with longer or shorter side chains, have no anti-influenza effect. Moreover, the synthesized derivatives proved to be ineffective against the tested influenza B strain, which is common in anti-influenza compounds due to the different structural and binding properties of their HA and NA [40]. Activities against influenza A strains demonstrate that butyl and hexyl chains may be too short for proper interactions, while decyl chains make the compounds highly toxic. The cLogP values of octyl derivatives are almost identical for **12b** and **18b** (5.3–5.4) and significantly lower for **23a**, which may explain the ineffectiveness of **23a** against influenza strains compared to the other two octyl derivatives. From the point of view of lipophilicity, it would have been more appropriate to use a shorter ethylene glycol linker instead of tetraethylene glycol in the case of the third series. In addition to differences in lipophilicity, many other things can affect the biological activity of a compound. These amphiphilic molecules can act in the form of aggregates and may bind to the cell membrane (or to the viral membrane) with their lipophilic tails [48] as well; therefore, they may also exert their effect directly on the cell’s surface or at the viral membrane. Both in the cell membrane and in aggregates, the shape of the molecule can influence the orientation of the neuraminic acid moiety, which must be appropriate for binding to hemmaglutinin and neuraminidase. 

Using DLS measurements, we proved that the synthesized derivatives (**12a**,**b**, and **c** with decyl, octyl, and butyl chains) tend to form aggregates in aqueous media, as we assumed, and the aggregates formed by an octyl derivative (**12b**) have the smallest average diameter, which also supports the privileged role of the presence of octyl chains.

The affinity of the prepared octyl derivatives for two types of influenza hemagglutinins (A H1N1 and H3N2) and neuraminidase of an influenza H1N1 strain was determined by surface plasmon resonance (SPR) technique. All tested compounds were bound to all three immobilized proteins, of which compound **12b** was the strongest binding partner with 161 and 124 μM dissociation constant values toward hemagglutinins and half the affinity towards neuraminidase. The high affinity of **12b** toward HAs can be explained by its self-assembly and multivalent interaction with the viral glycoproteins; however, other mechanisms may also contribute to the effect. From these results, we can regard **12b** as a dual-specific inhibitor of influenza hemagglutinin and neuraminidase. While the effectiveness of **12b** is not outstanding, we can potentially improve the anti-influenza effects by fine-tuning the structure and synthesizing similar derivatives.

## Figures and Tables

**Table 1:** Anti-influenza virus activity in MDCK cells.

Compound	Antiviral EC_50_ (µM) ^a^						Cytotoxicity (µM)	
	IAV (A/H1N1)		IAV (A/H3N2)		IBV			
	CPE	MTS	CPE	MTS	CPE	MTS	MCC ^b^	CC_50_^c^
**12a**	>100	>100	>100	>100	>100	>100	20 ^d^	64 ^d^
**12b**	8.9 ± 0	15 ± 5	8.9 ± 0	8.7 ± 3.6	>100	>100	39 ± 20	34 ± 13
**12c**	>100	>100	>100	>100	>100	>100	100 ± 0	>100
**18a**	>100	>100	>100	>100	>100	>100	20 ^d^	10 ^d^
**18b**	25 ^d^	23 ^d^	4.8 ± 1.0	6.2 ± 3.0	>100	>100	63 ± 19	32 ± 8
**18c**	>100	>100	>100	>100	>100	>100	100 ^d^	>100
**23a**	>100	>100	>100	>100	>100	>100	40 ^d^	54 ^d^
**23b**	>100	>100	>100	>100	>100	>100	>100	>100
Zanamivir	10 ± 3	8.5 ± 3.4	1.6 ^d^	1.0 ^d^	2.1 ^d^	4.5 ^d^	>100	>100
Ribavirin	24 ± 6	25 ± 2	30 ± 6	24 ± 4	10 ± 4	5.4 ^d^	100 ± 0	>250

^a^ EC_50_: 50% effective concentration, i.e., compound concentration producing 50% inhibition of virus replication, as estimated by microscopic scoring of the cytopathic effect (CPE) or by MTS cell viability assay. ^b^ MCC: minimum cytotoxic concentration, i.e., minimum compound concentration causing microscopically visible alterations in cell morphology. ^c^ CC_50_: 50% cytotoxic concentration, as determined by MTS cell viability assay. Virus strains: A/Virginia: ATCC3/2009 or A/PR/8 (A/H1N1); A/Victoria/361/11 or A/HK/7/87 (A/H3N2); and B/Ned/537/05 (IBV). Cell line: Madin-Darby canine kidney (MDCK) cells. Data shown are the average values ± SEM of 2–4 independent tests, except for the values indicated with suffix ^d^, which are from single experiments.

**Table 2:** Affinity toward hemagglutinins and neuraminidase determined by SPR.

Compound	H1N1 Hemagglutinin		H3N2 Hemagglutinin		H1N1 Neuraminidase	
	K_D_(app) [μM]	R_max_ [RU]	K_D_(app) [μM]	R_max_ [RU]	K_D_(app) [μM]	R_max_ [RU]
**12b**	161 ± 20	130 ± 12	124 ± 21	135 ± 11	286 ± 97	175 ± 23
**18b**	ND	ND	ND	ND	1106 ± 430	166 ± 53
**23a**	6220 ± 4044	505 ± 275	1217 ± 737	148 ± 43	502 ± 264	74 ± 13

**Table 3:** Dynamic light scattering measurements of **12a**, **12b,** and **12c**.

	12a	12b	12c
Concentration	d (nm)		
1 mg/mL	126 (2.00%) ^a^	96 (1.19%) ^a^	103 (28.3%) ^a^
0.1 mg/mL	170 (15.8%) ^a^	108 (13.4%) ^a^	111 (14.2%) ^a^

^a^ Calculated RSD values from 3 repeated measurements.

**Table 4:** CLogP values of the synthesized derivatives.

1st Series	cLogP	2nd Series	cLogP	3rd Series	cLogP
**12a**	7.52045	**18a**	7.4555		
**12b**	5.40445	**18b**	5.3395	**23a**	4.2715
**12c**	1.17245	**18c**	3.2235	**23b**	2.1555

## Data Availability

The datasets used and/or analyzed during the current study are available from the corresponding author (I.B.) upon reasonable request.

## Supplementary Materials

The following supporting information can be downloaded at https://www.mdpi.com/article/10.3390/ijms242417268/s1.
