# Supplementary material for: Amphiphilic Sialic Acid Derivatives as Potential Dual-Specific Inhibitors of Influenza Hemagglutinin and Neuraminidase
Source: Int J Mol Sci. 2023 Dec 8;24(24):17268. doi: 10.3390/ijms242417268 (PMC10743929; doi:10.3390/ijms242417268)
Supplement: Supplementary file 1 [file ijms-24-17268-s001.zip › ijms-2707832-supplementary.pdf]

Supplementary Information

Amphiphilic sialic acid derivatives as potential dual-specific inhibitors of influenza hemagglutinin and neuraminidase

Eszter Boglárka Lőrincz, Mihály Herczeg, Josef Houser, Martina Rievajová, Ákos Kuki, Lenka Malinovská, Lieve Naesens, Michaela Wimmerová, Anikó Borbás, Pál Herczegh, Ilona Bereczki

Table of content

Surface plasmon resonance measurements in the absence of DMSO S2
Dynamic light scattering measurements S3
NMR spectra S6

Surface plasmon resonance measurements in the absence of DMSO

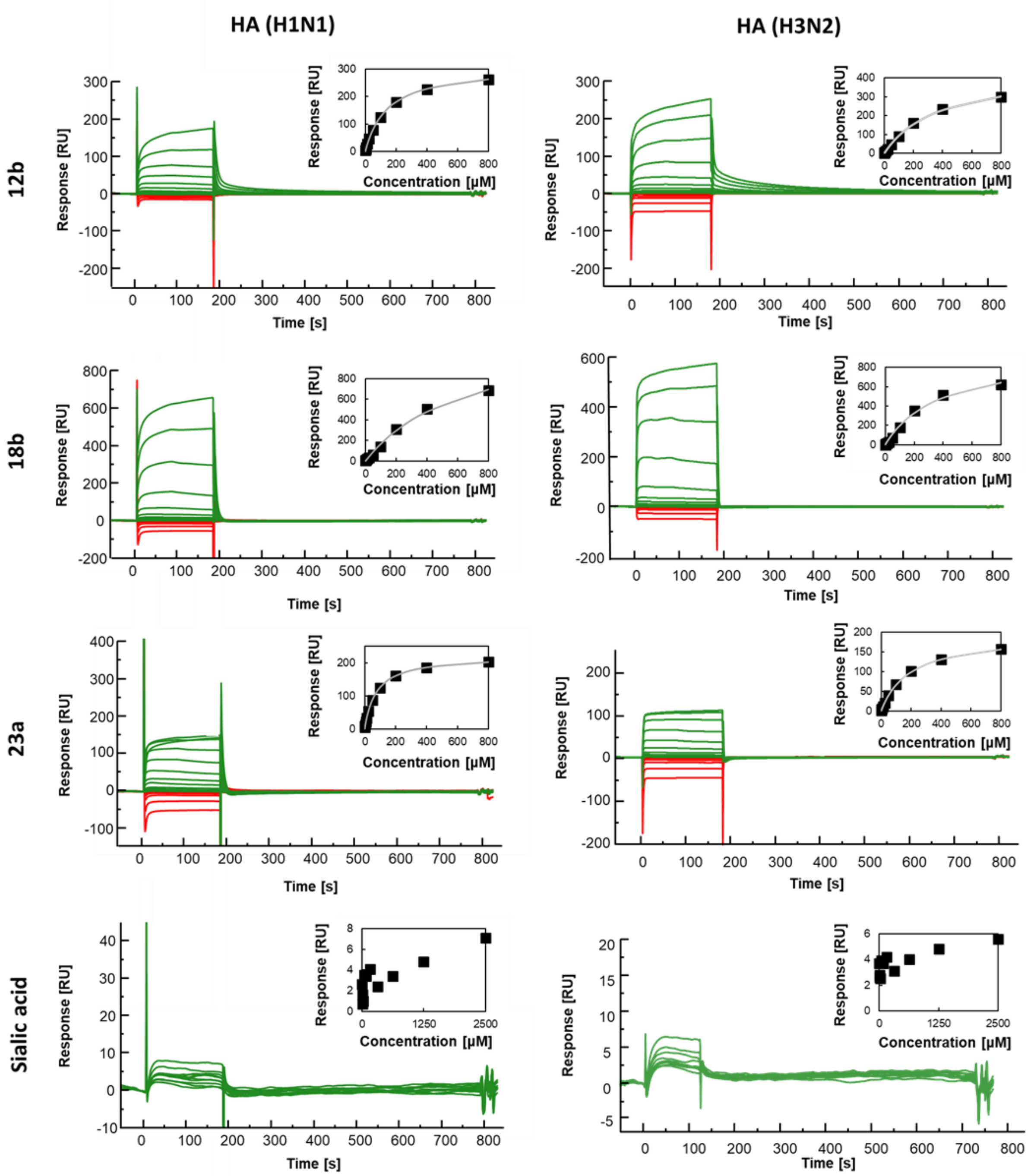

Figure S1. SPR sensorgrams. Example of a single replicate of compounds binding to immobilized hemagglutinin from H1N1 and H3N2 influenza strain. Green: Sensorgrams of two-fold dilution row of each compound. Red: Sensorgrams of corresponding DMSO injections (not applicable for sialic acid). Inset: Steady-state response after DMSO-correction as black squares, one-site binding model fit in grey (fit not shown for sialic acid).

Table S1. Affinity of tested compounds towards hemagglutinins determined by SPR in the absence of DMSO.

Compound	H1N1 hemagglutinin		H3N2 hemagglutinin	
	K_D(app) [µM]	R_{max} [RU]	K_D(app) [µM]	R_{max} [RU]
**12b**	187 ± 41	305 ± 30	279 ± 98	454 ± 39
**18b**	286 ± 19	783 ± 8	357 ± 23	925 ± 23
**23a**	106 ± 36	197 ± 24	134 ± 48	200 ± 23

Dynamic light scattering measurements

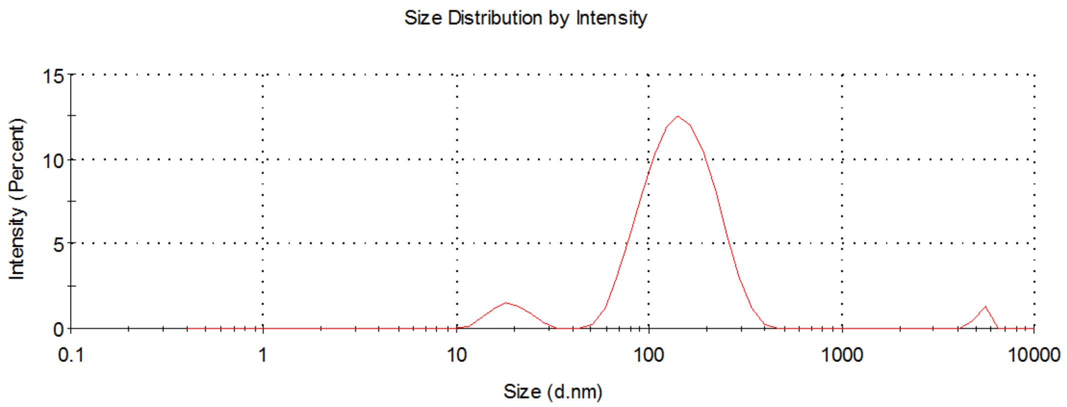

Figure S2. Size distribution of **12a** in the concentration of 1 mg/ml

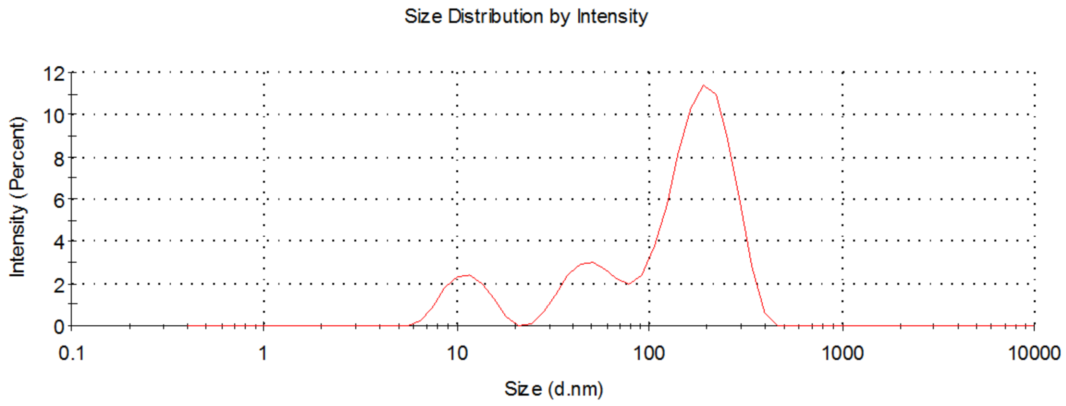

Figure S3. Size distribution of **12a** in the concentration of 0.1 mg/ml

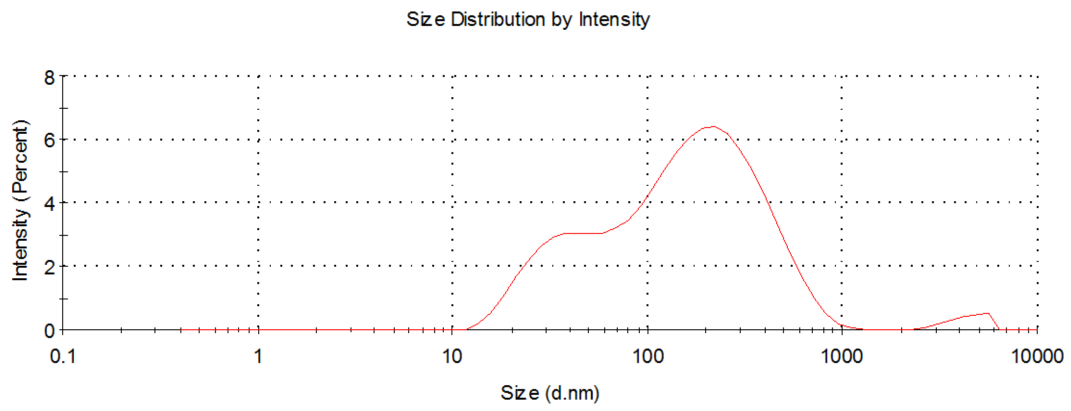

Figure S4. Size distribution of **12b** in the concentration of 1 mg/ml

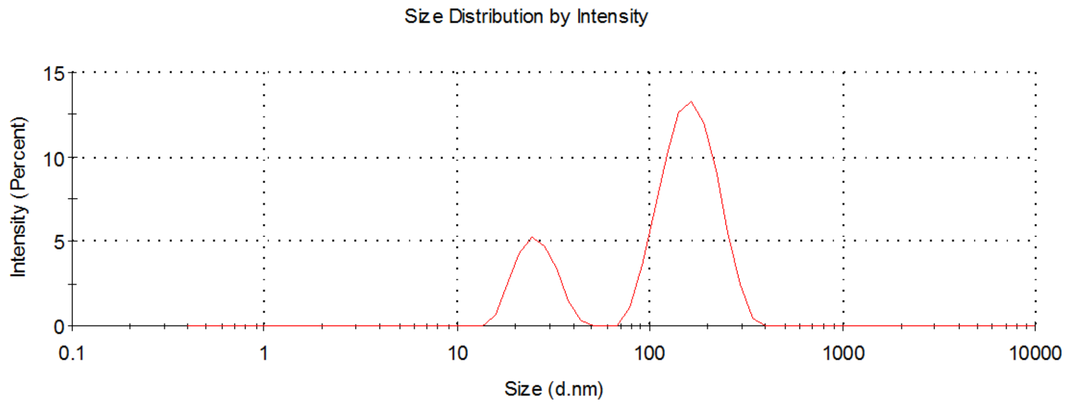

Figure S5. Size distribution of **12b** in the concentration of 0.1 mg/ml

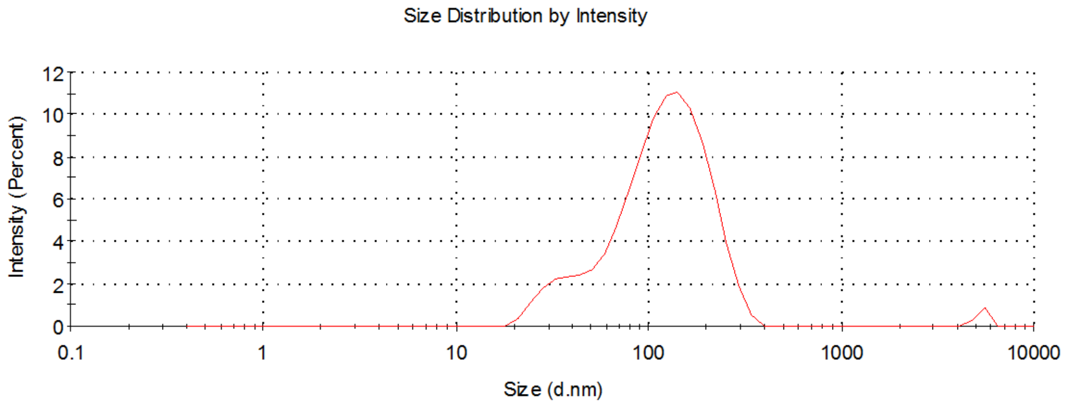

Figure S6. Size distribution of **12c** in the concentration of 1 mg/ml

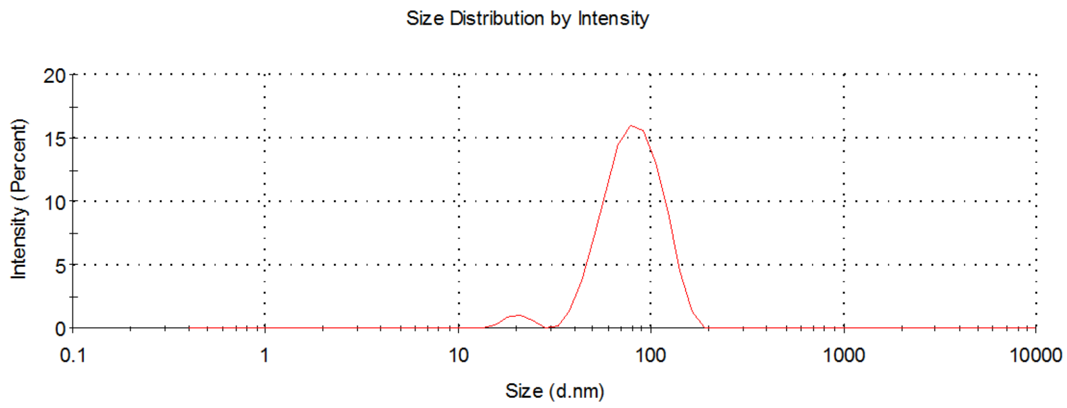

Figure S7. Size distribution of **12c** in the concentration of 0.1 mg/ml

NMR spectra

NMR spectra of compound **4a**

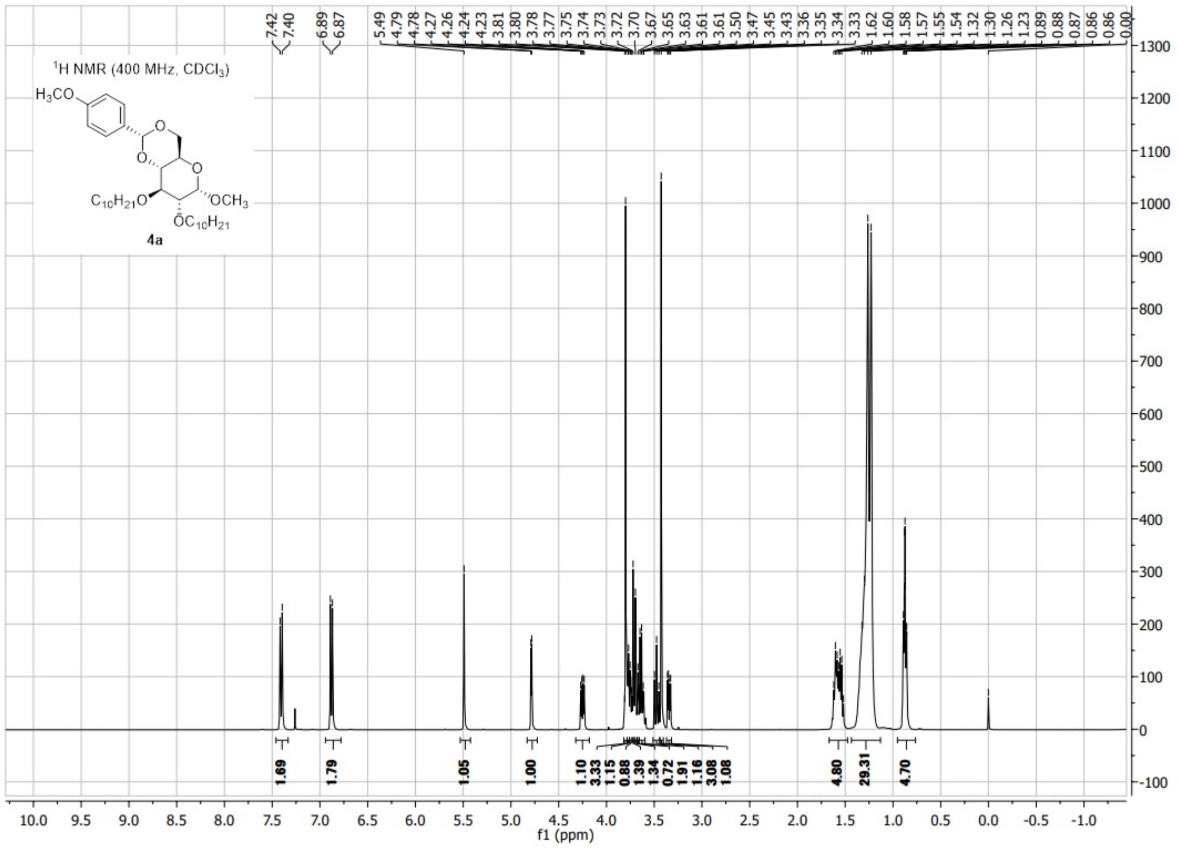

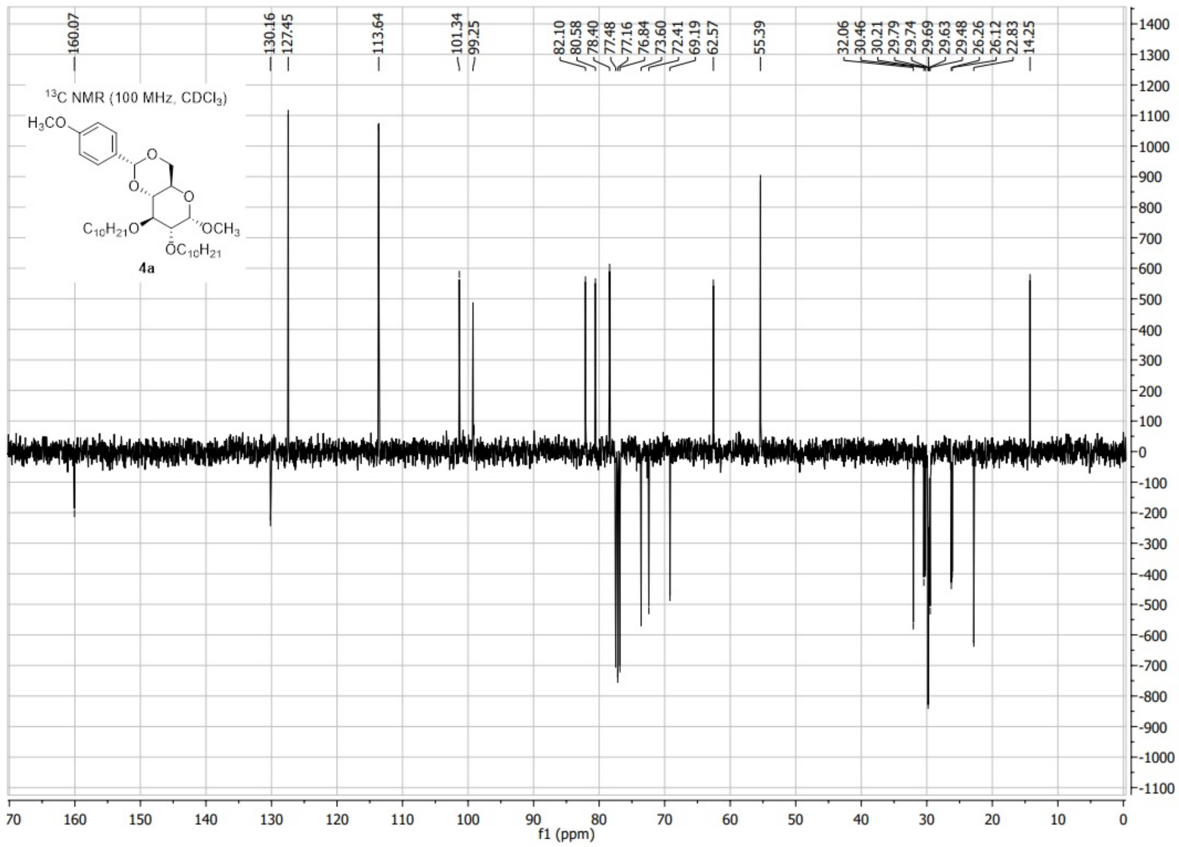

NMR spectra of compound **5a**

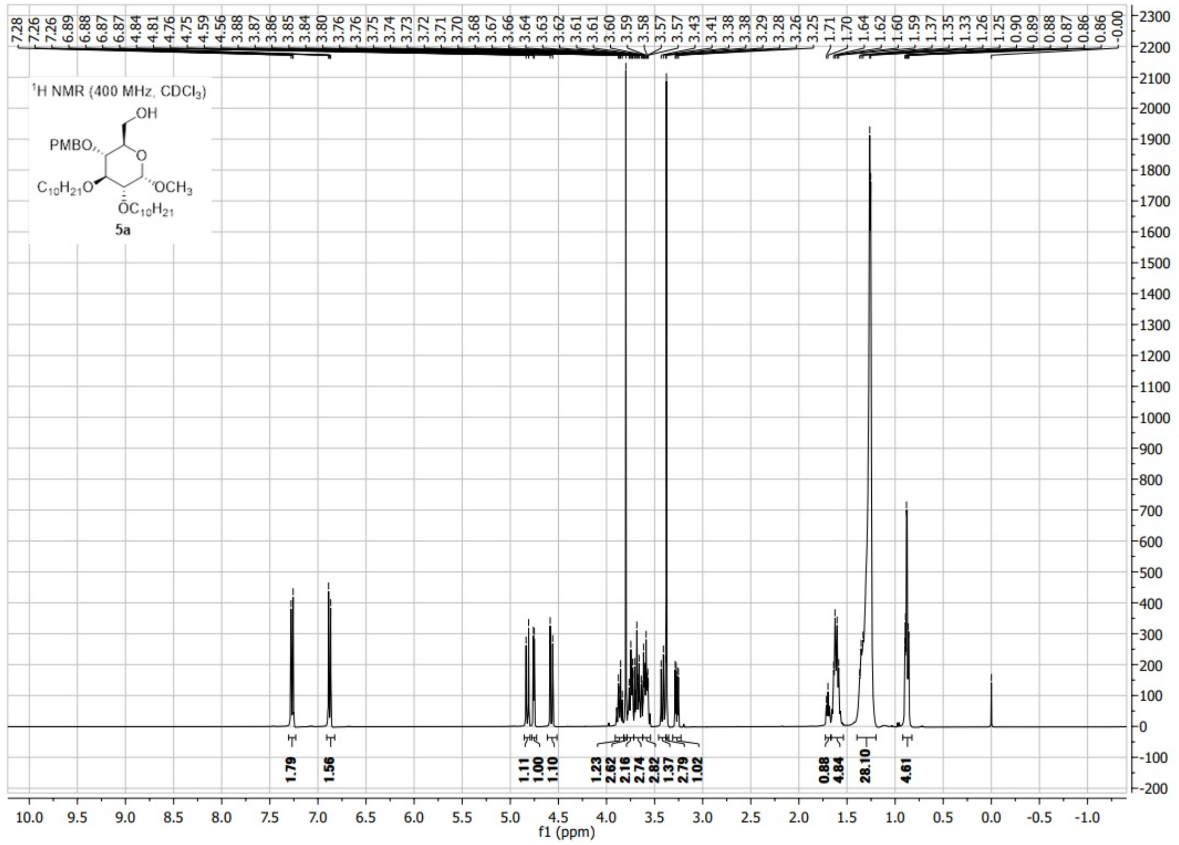

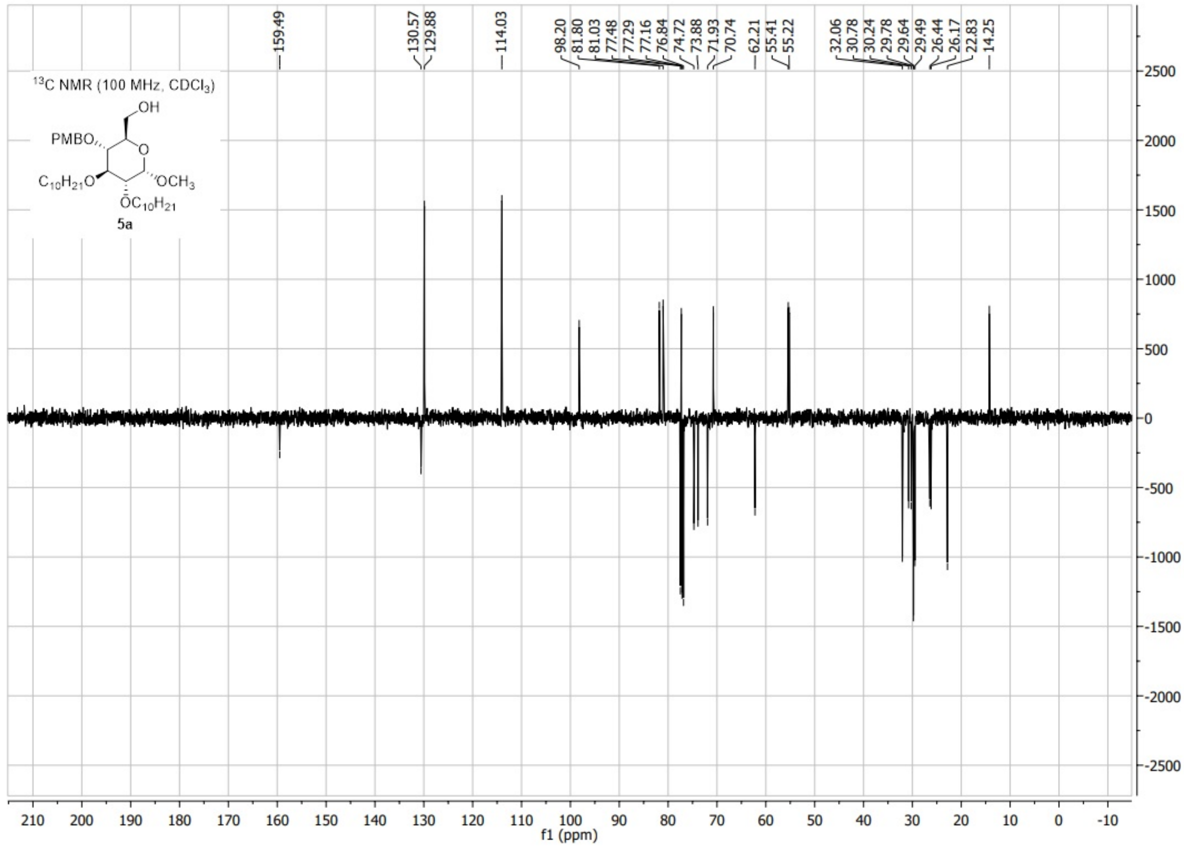

NMR spectra of compound **8a**

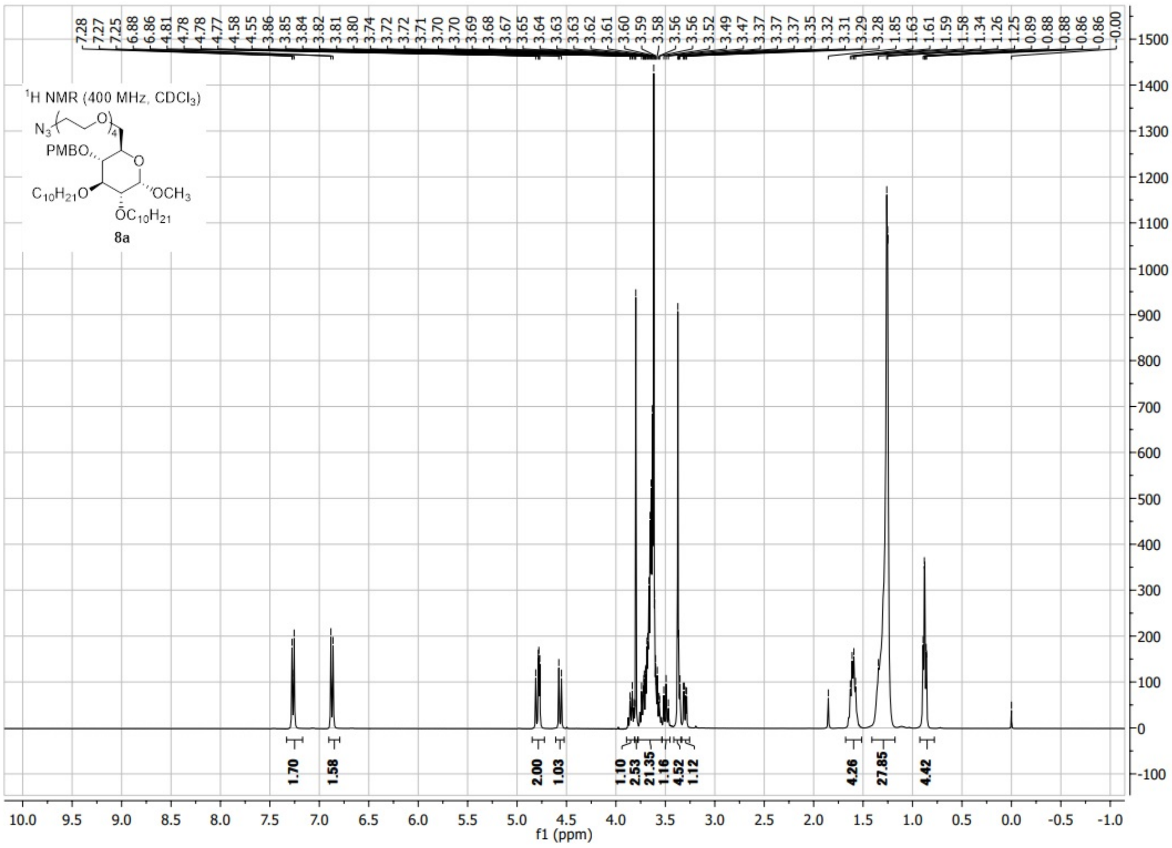

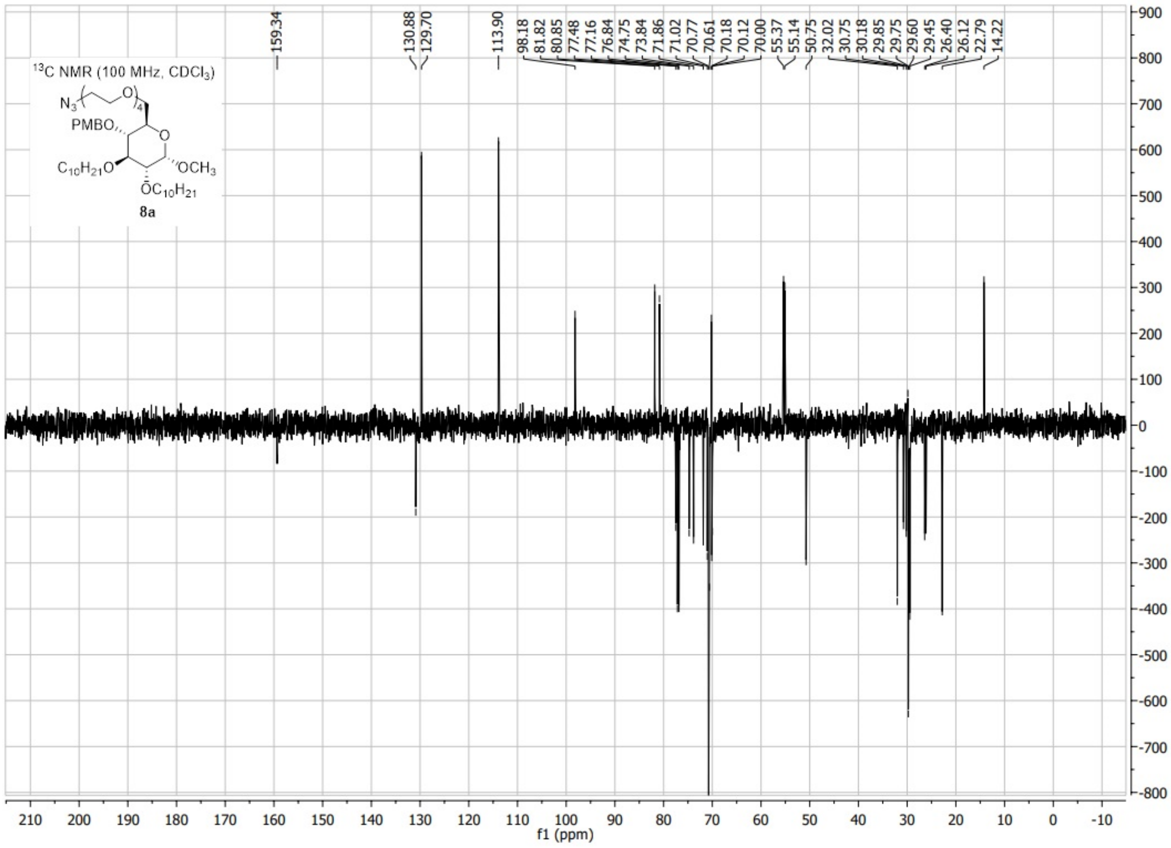

NMR spectra of compound **8b**

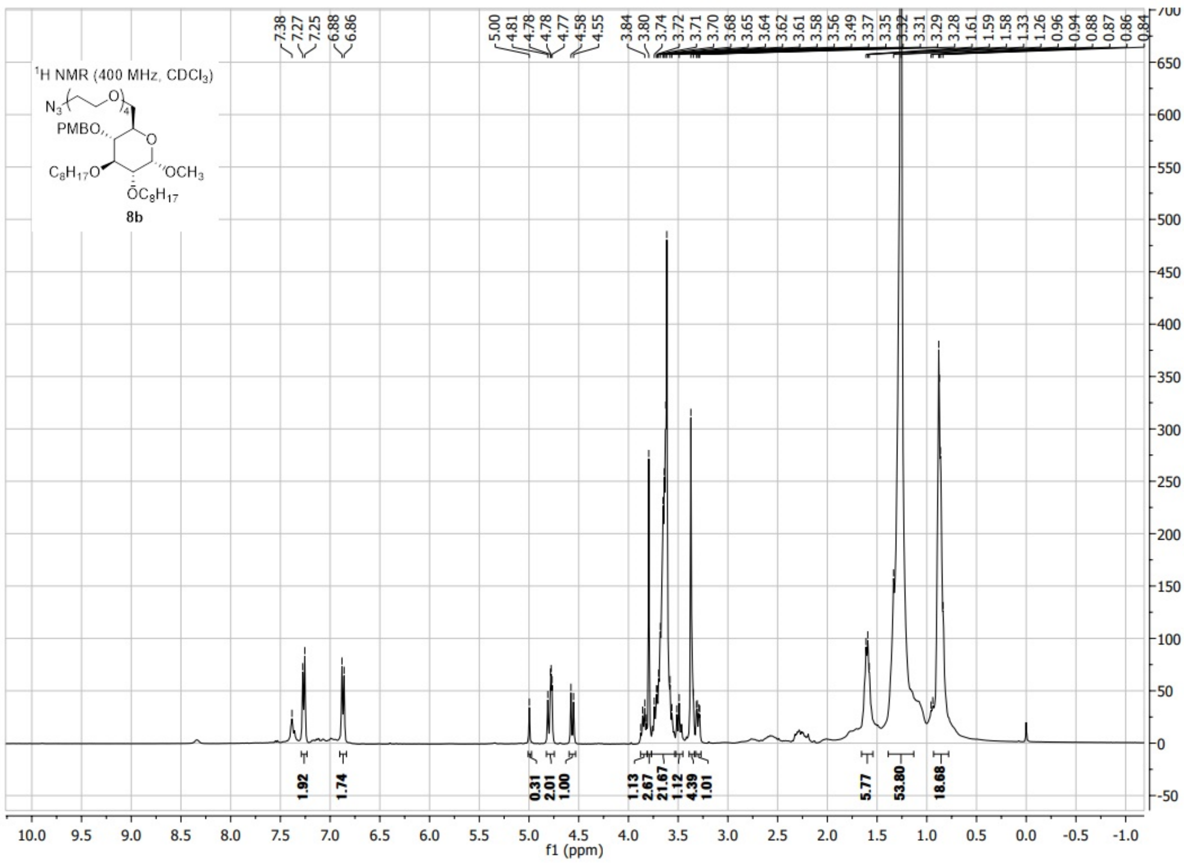

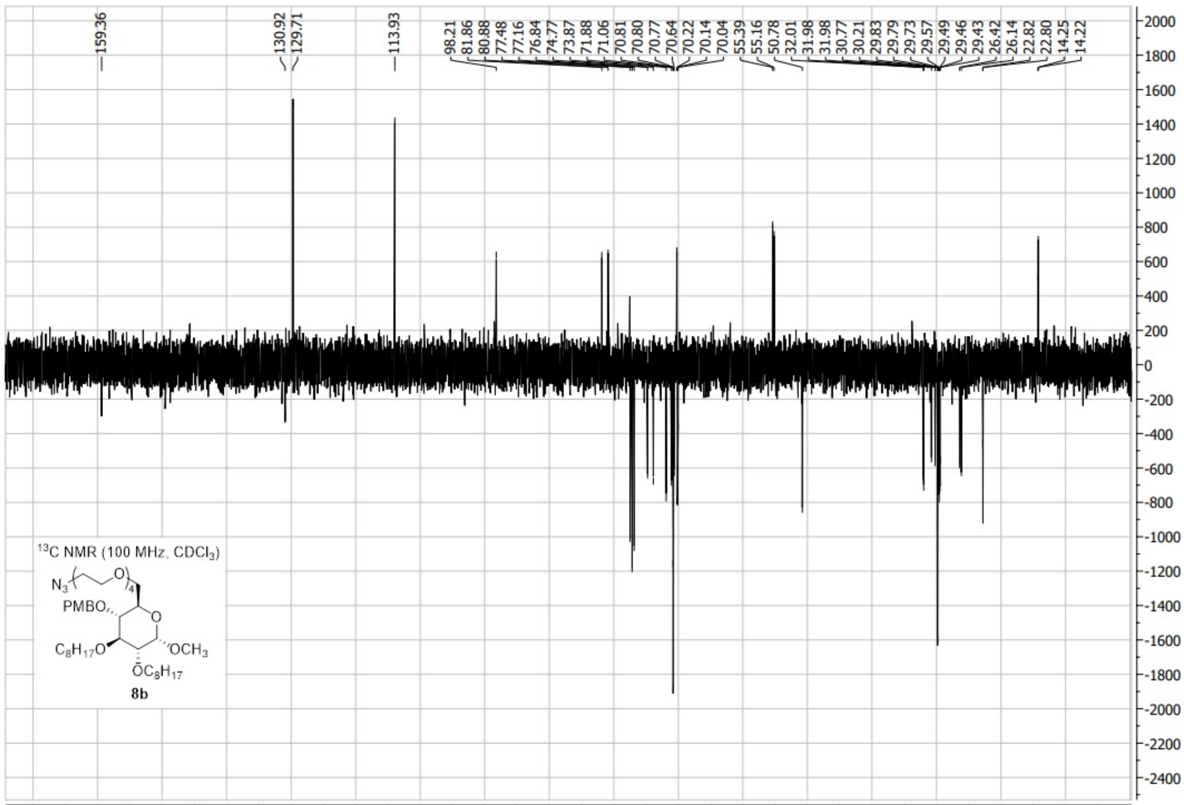

NMR spectra of compound **8c**

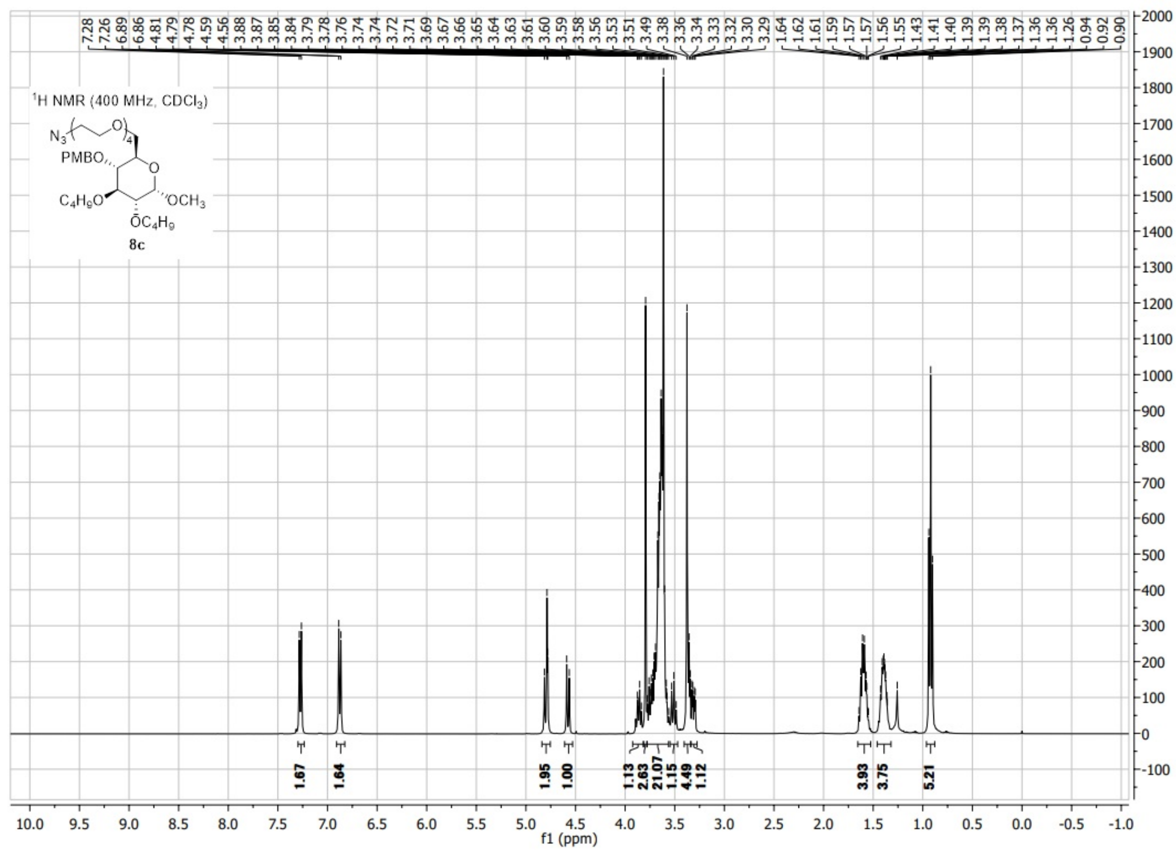

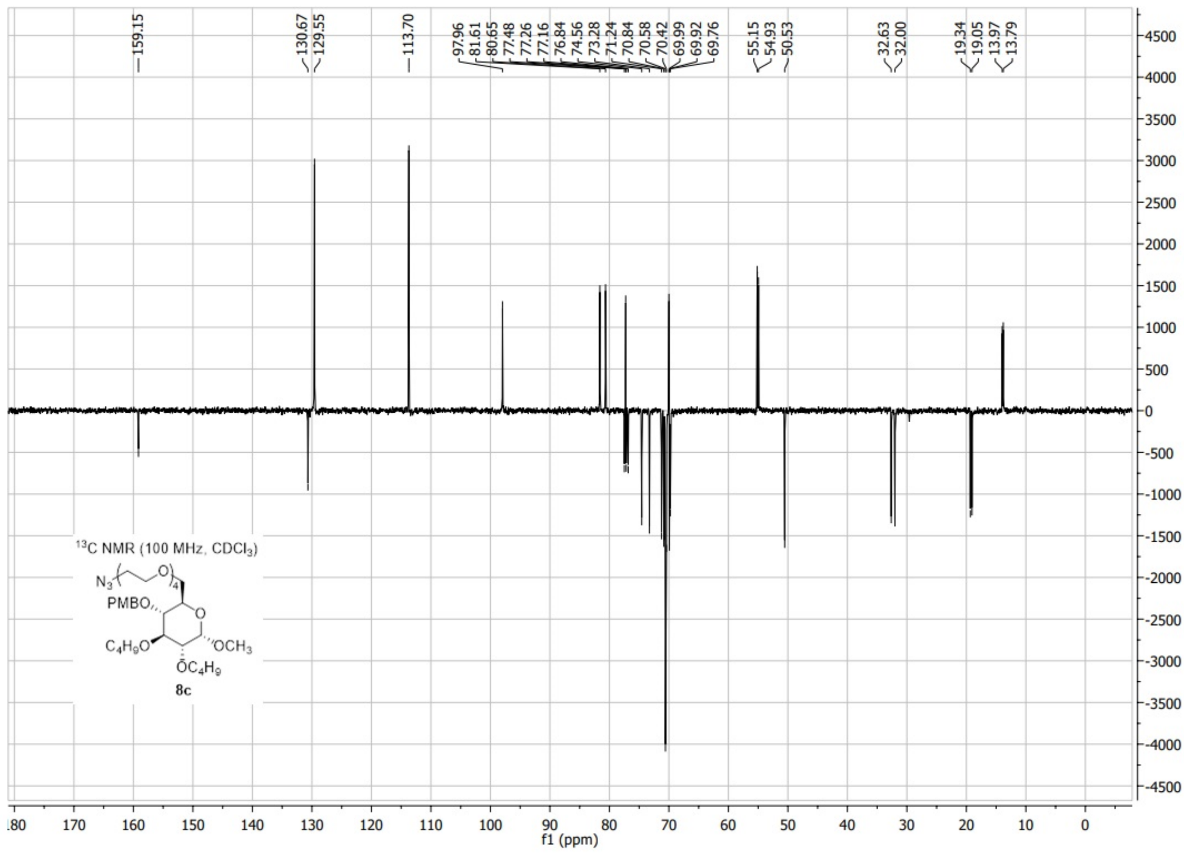

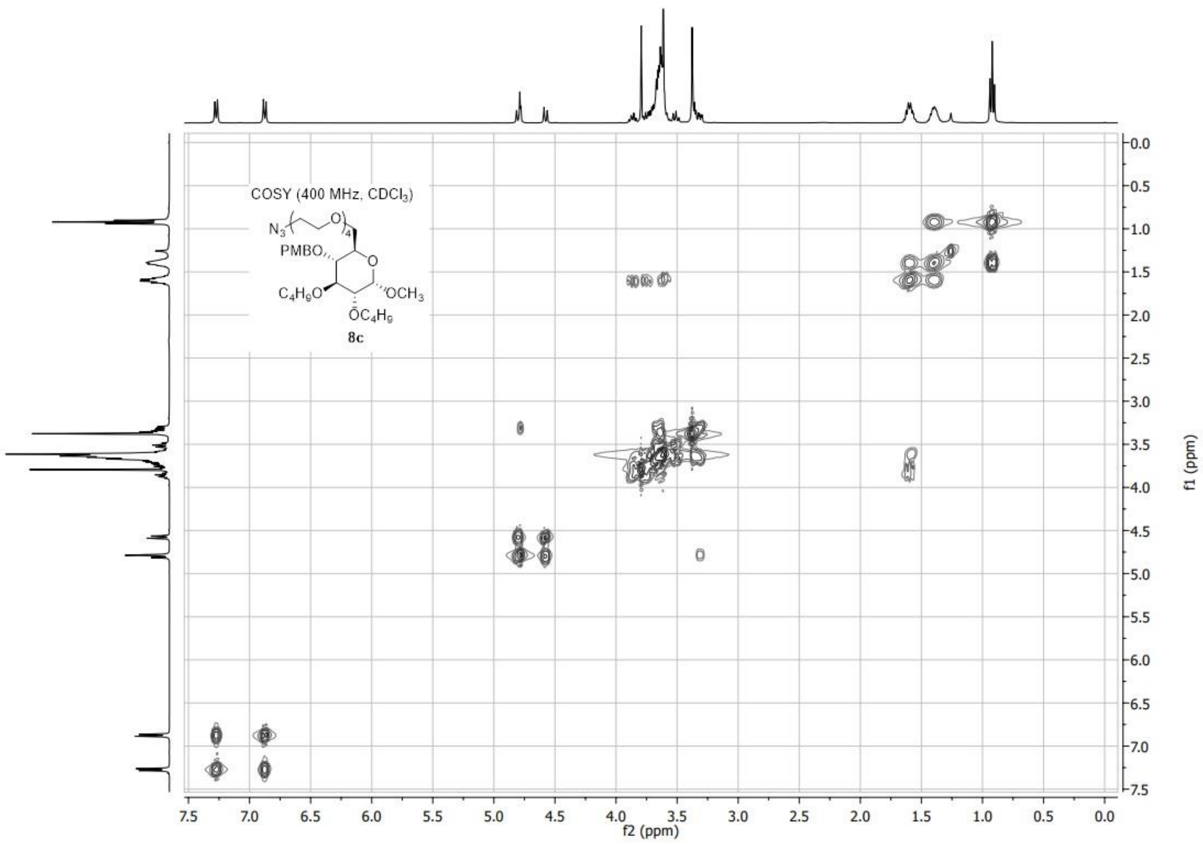
COSY (400 MHz, $CDCl_3$)
N_3
O
4
PMBO
O
C_4H_9O
OCH_3
OC_4H_9
8c
f2 (ppm)
f1 (ppm)

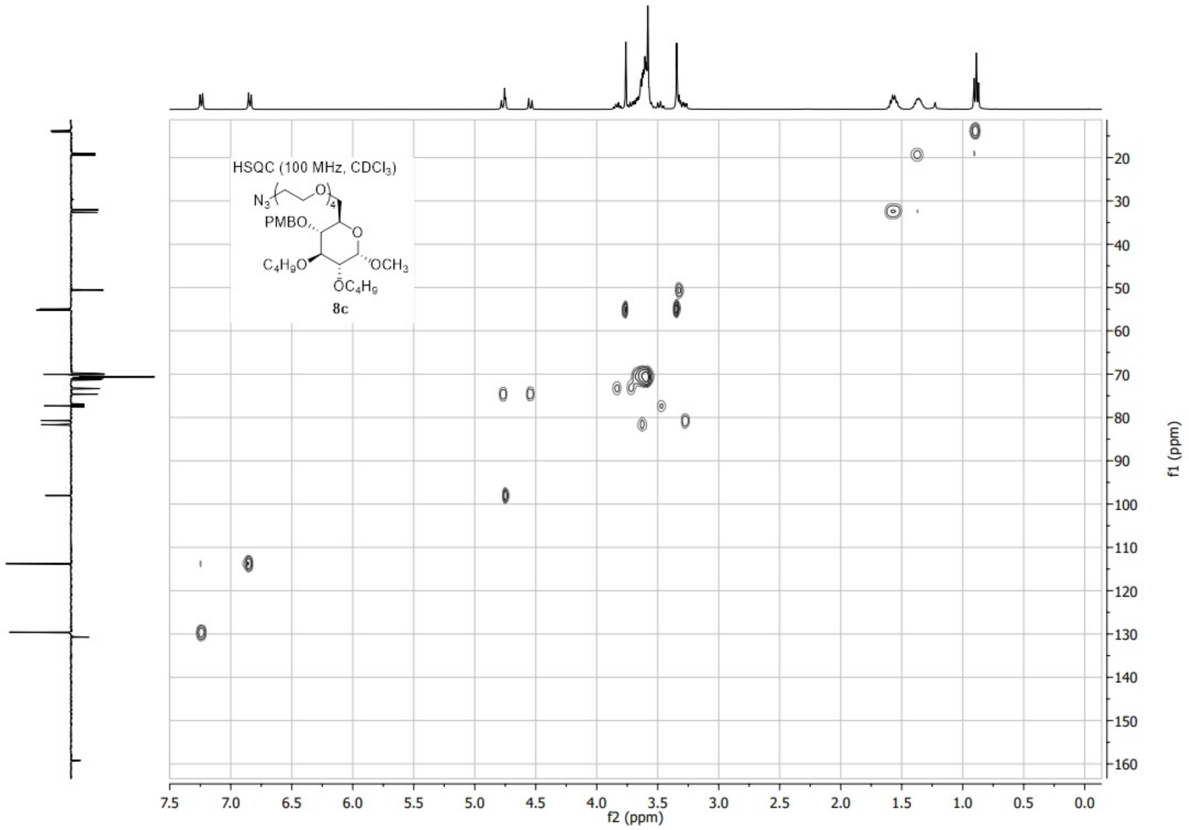
HSQC (100 MHz, $CDCl_3$)
N_3
O
4
PMBO
O
C_4H_9O
OCH_3
OC_4H_9
8c
f2 (ppm)
f1 (ppm)

NMR spectra of compound **12a**

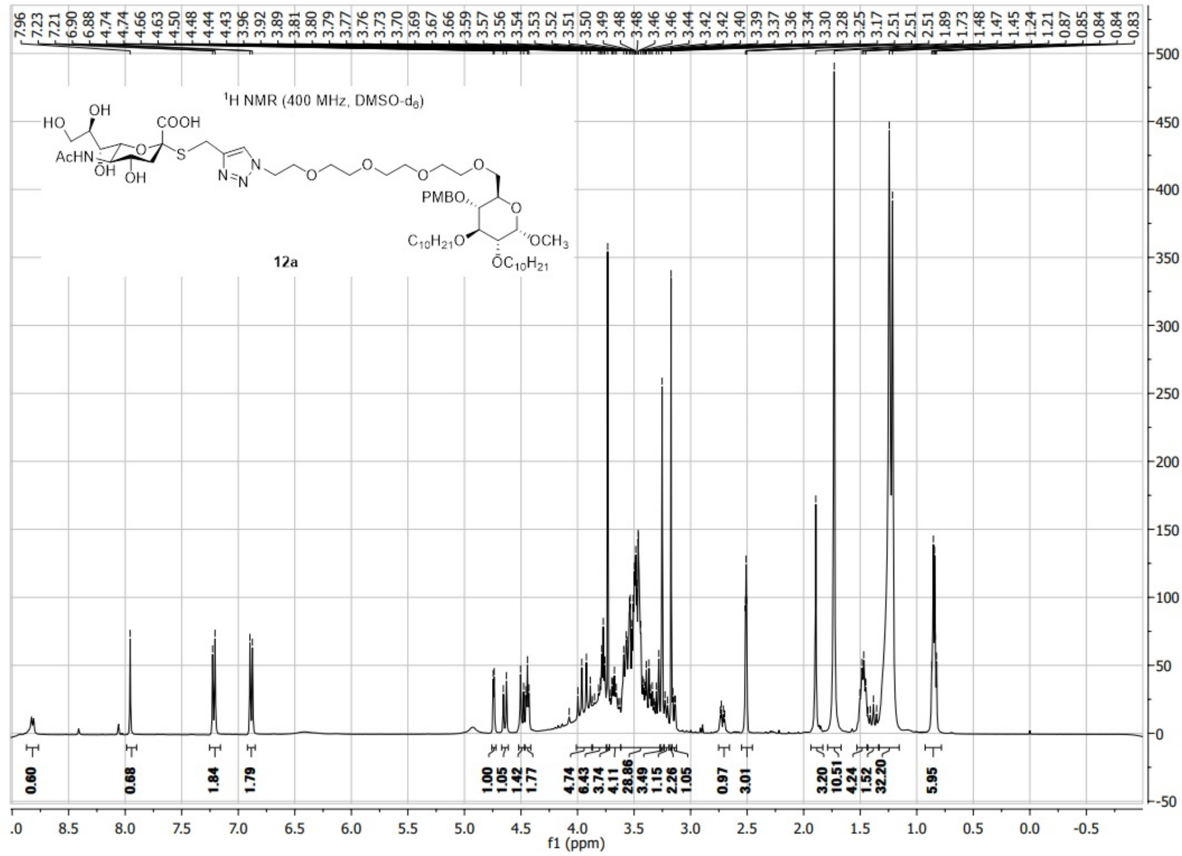

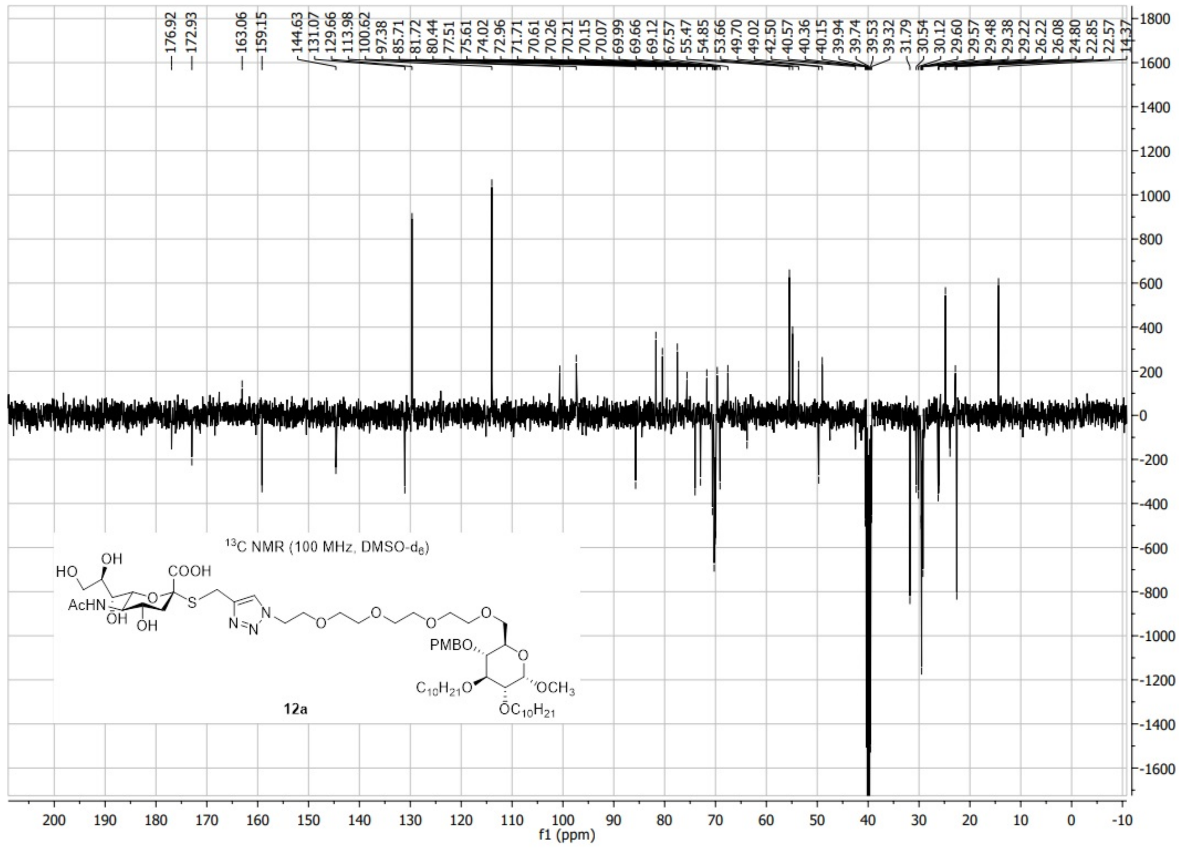

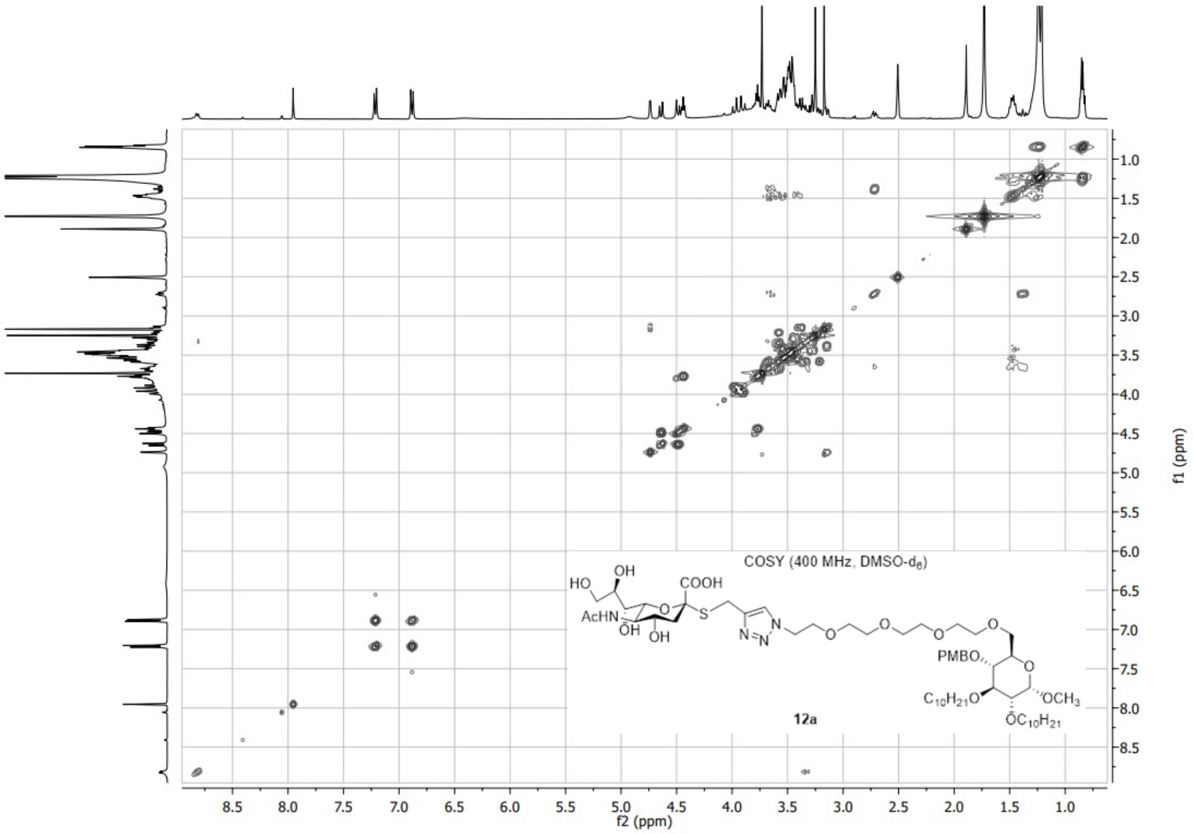
COSY (400 MHz, DMSO-d6)
12a
f2 (ppm)
f1 (ppm)

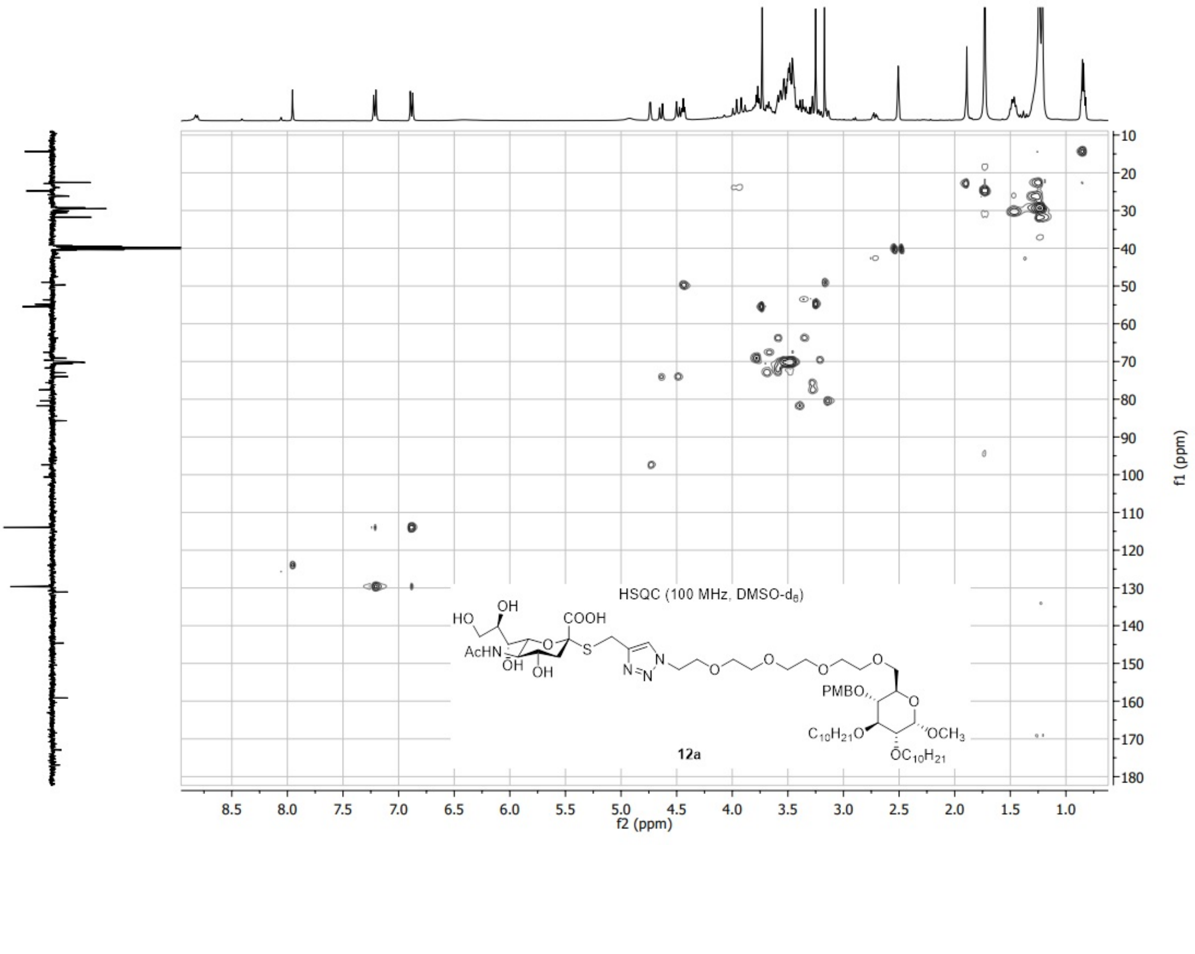
HSQC (100 MHz, DMSO-d6)
12a
f2 (ppm)
f1 (ppm)

NMR spectra of compound **12b**

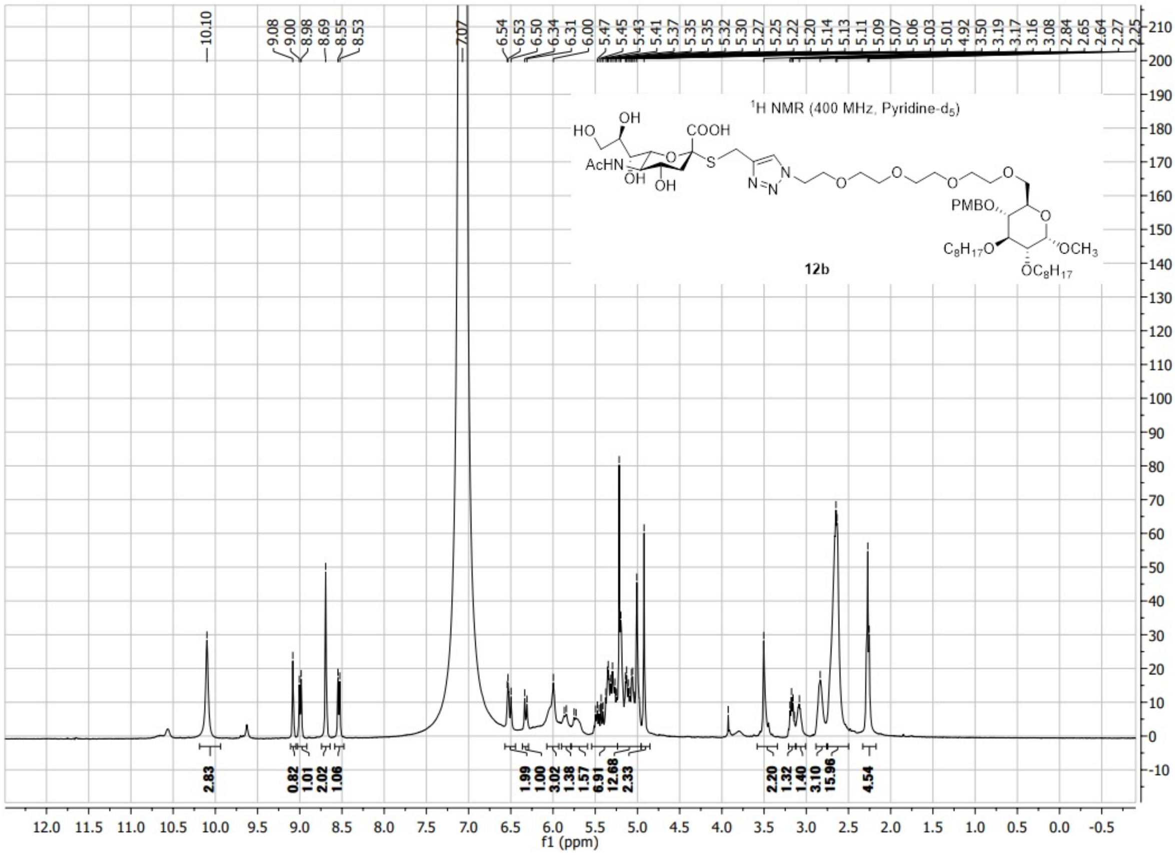

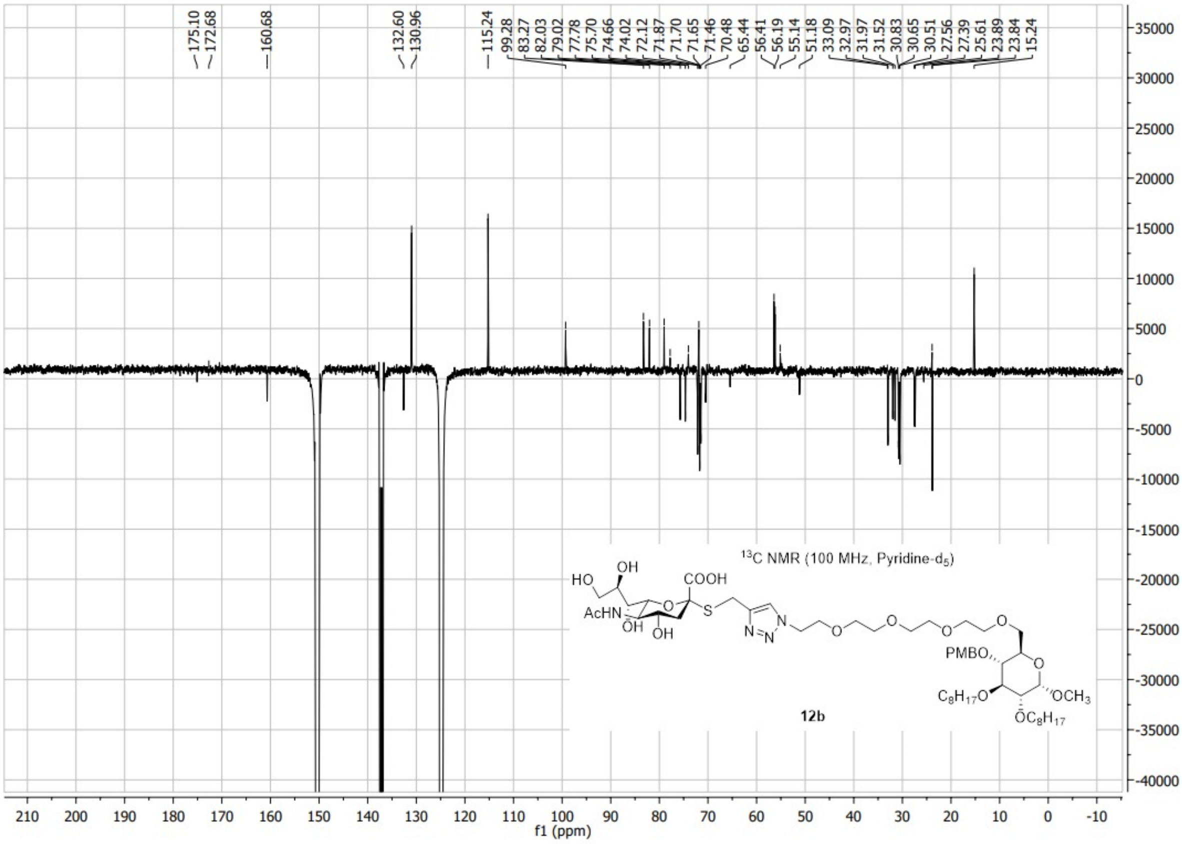

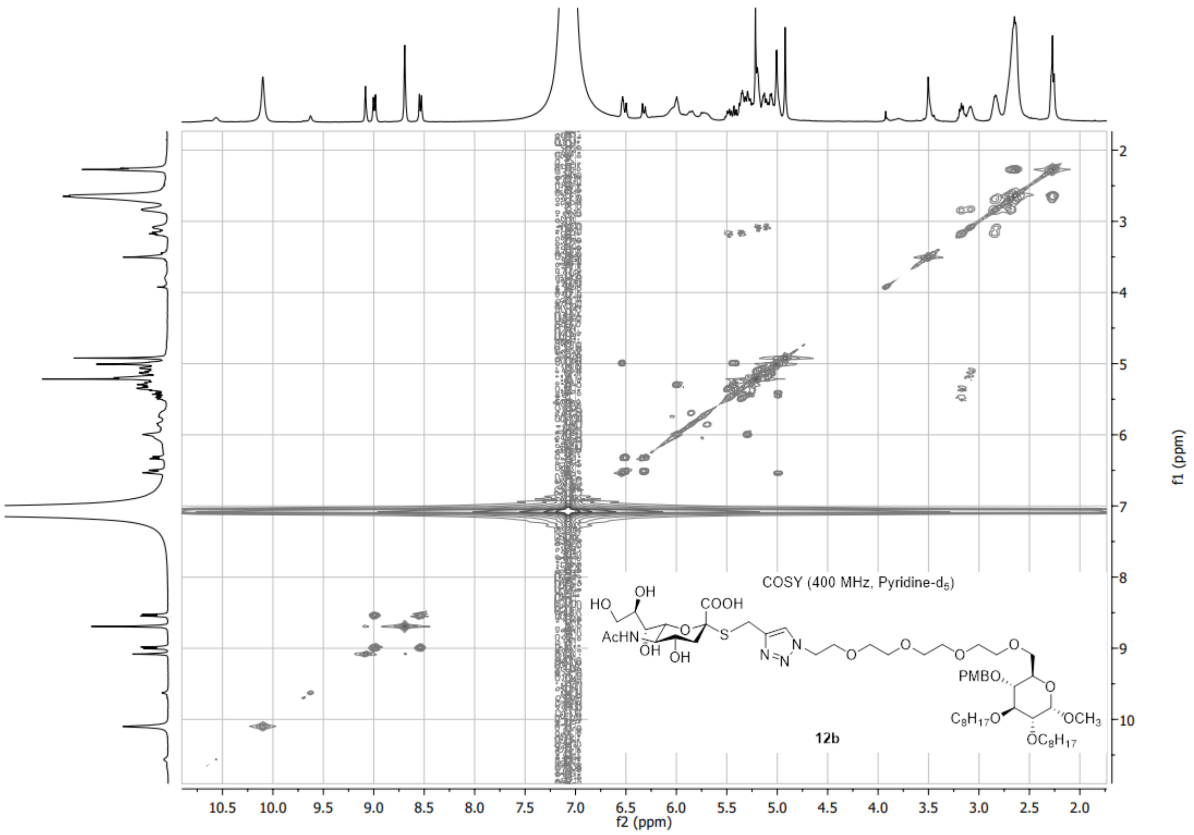
COSY (400 MHz, Pyridine-d5)
12b
f1 (ppm)
f2 (ppm)

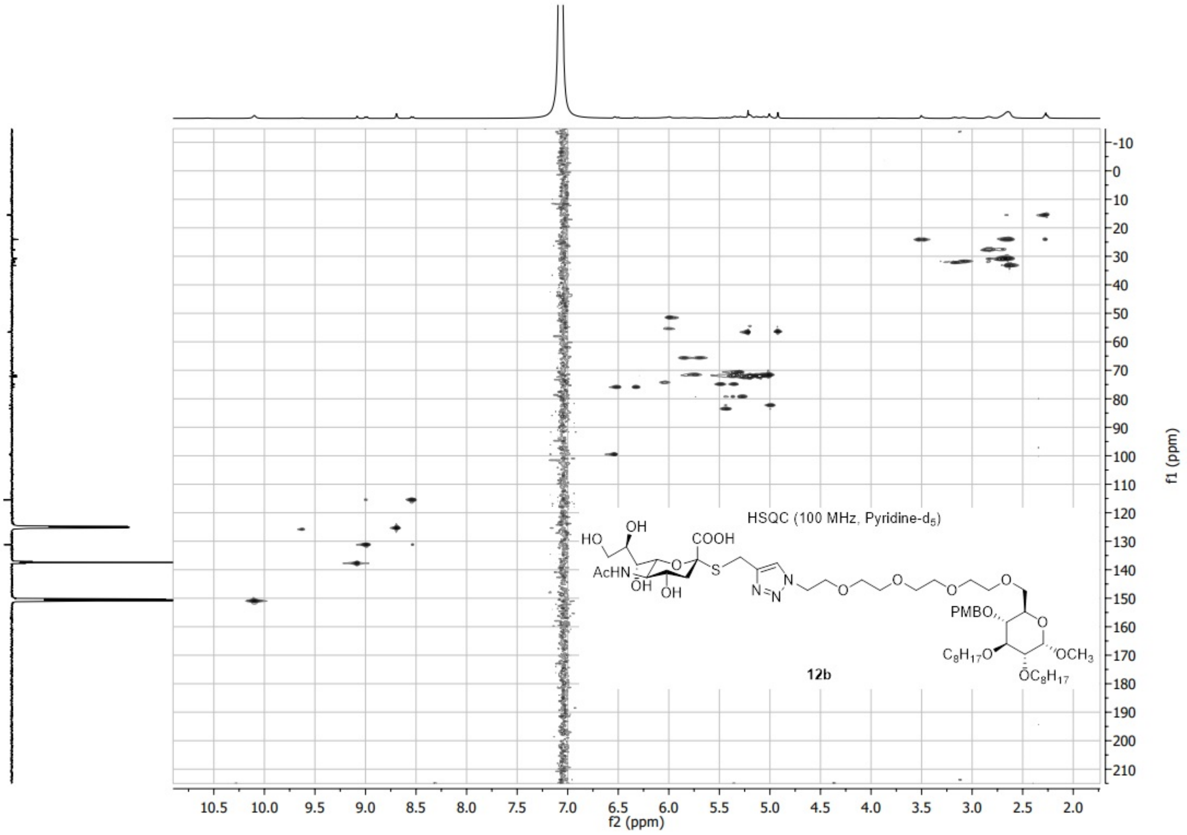
HSQC (100 MHz, Pyridine-d5)
12b
f1 (ppm)
f2 (ppm)

NMR spectra of compound **12c**

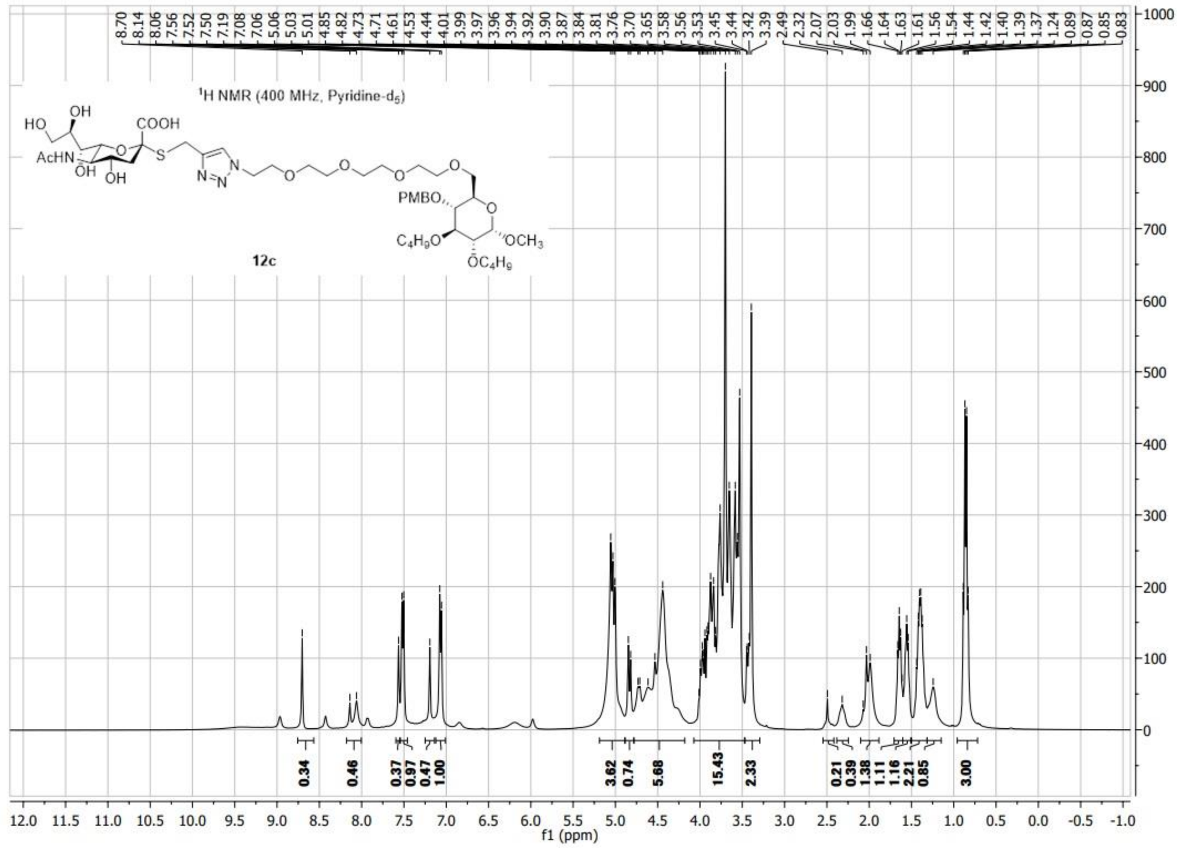

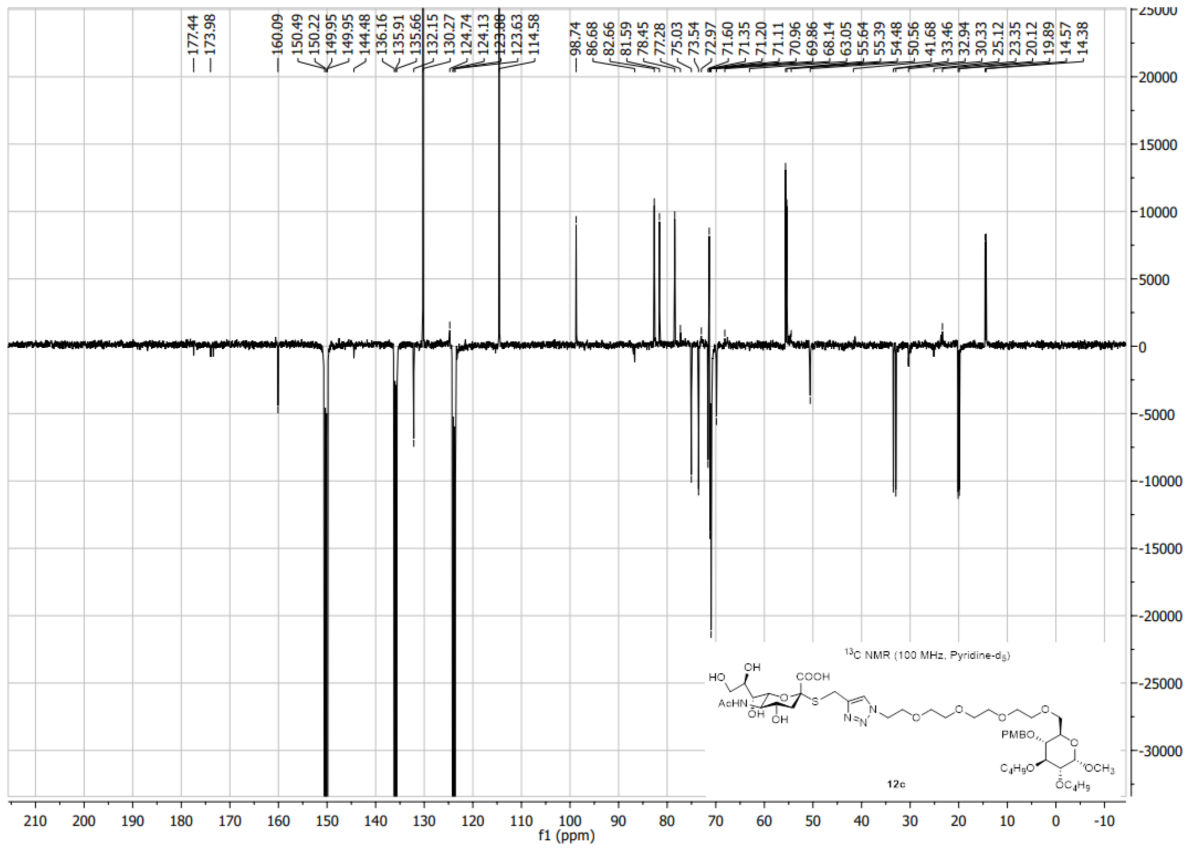

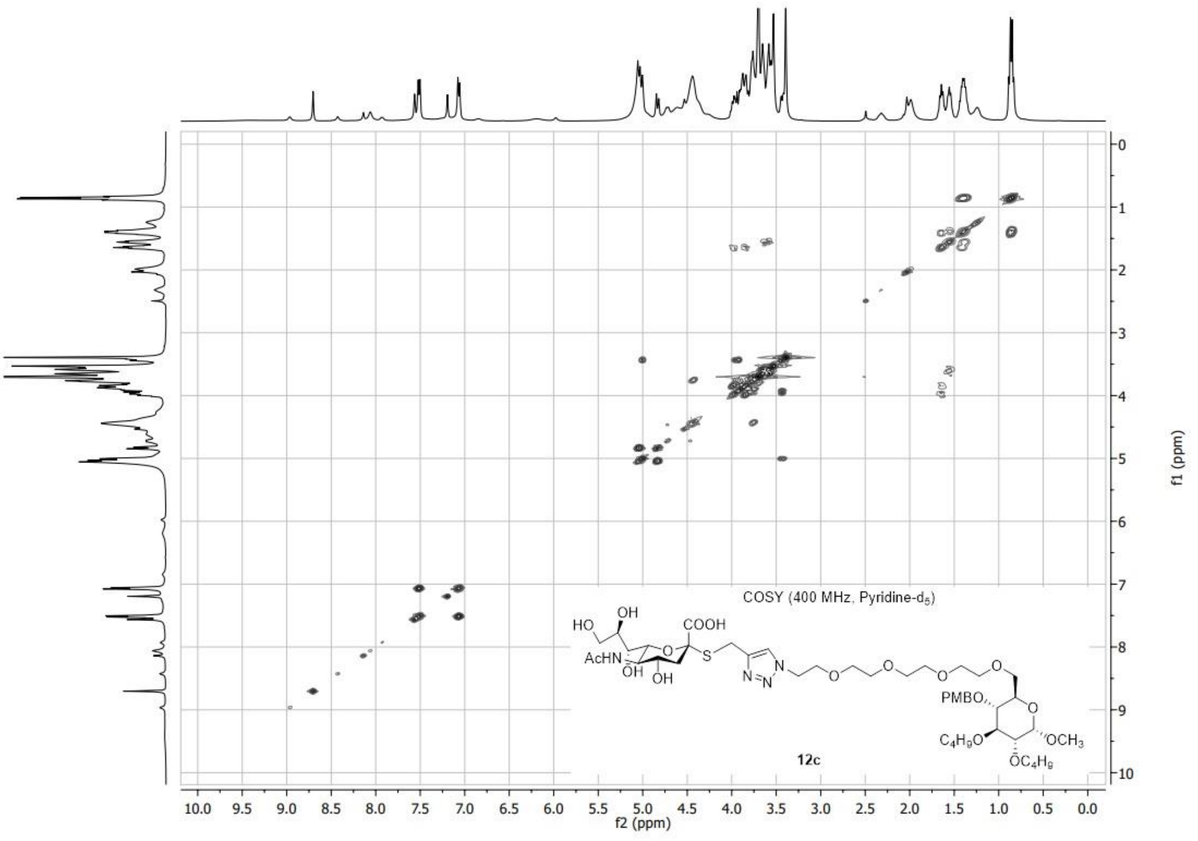
COSY (400 MHz, Pyridine-d5)
12c
f1 (ppm)
f2 (ppm)

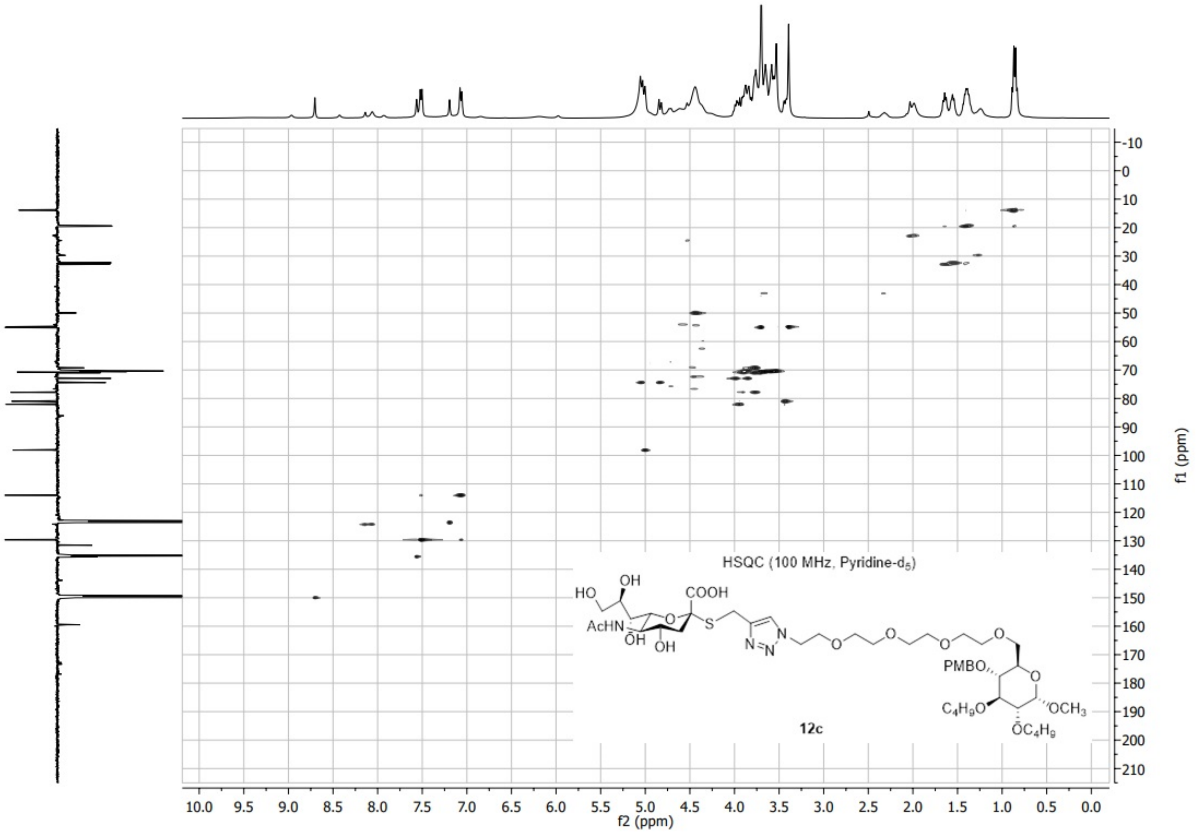
HSQC (100 MHz, Pyridine-d5)
12c
f1 (ppm)
f2 (ppm)

NMR spectra of compound **15a**

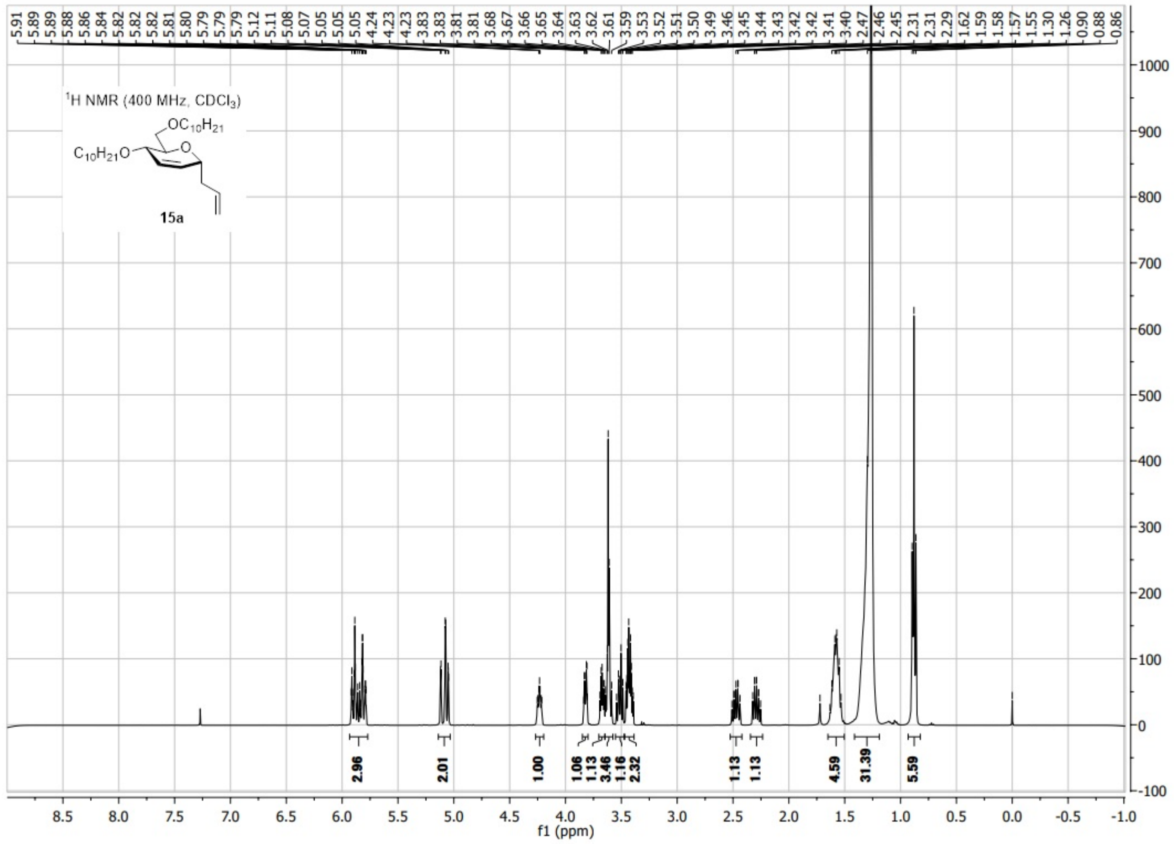

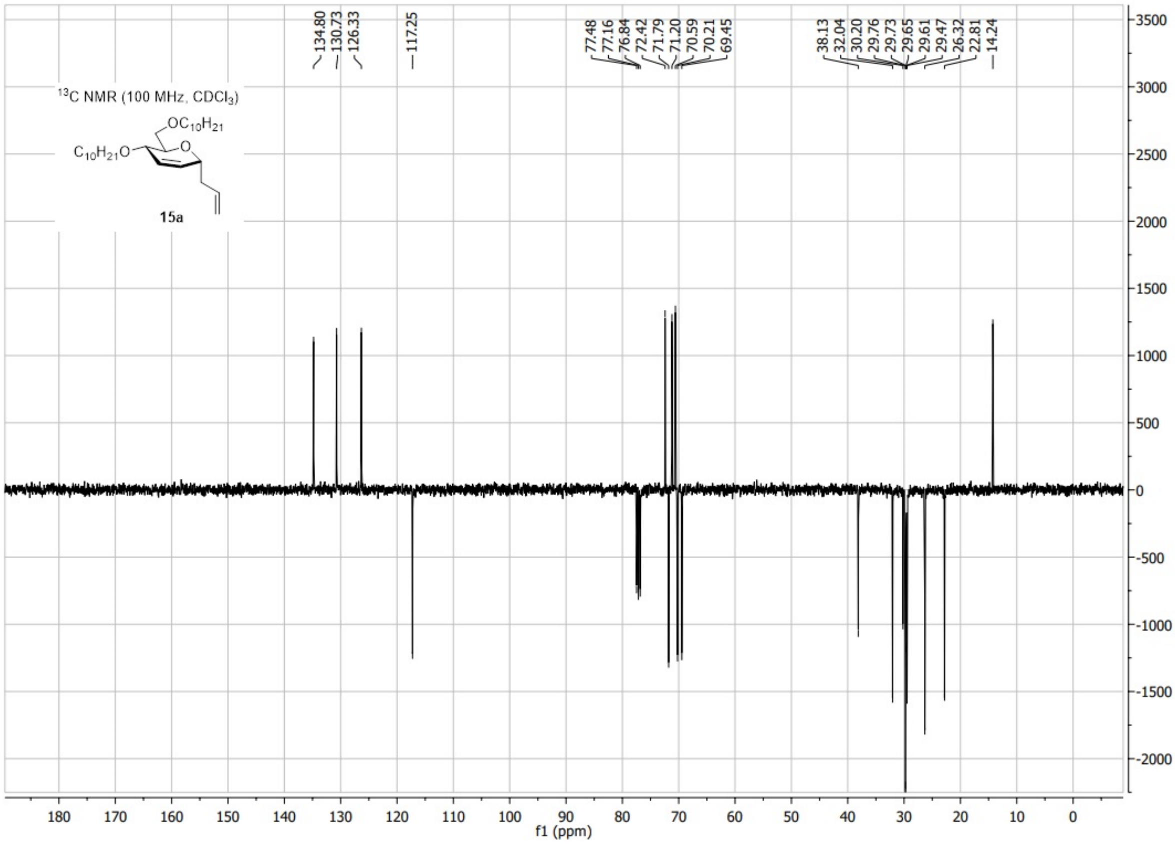

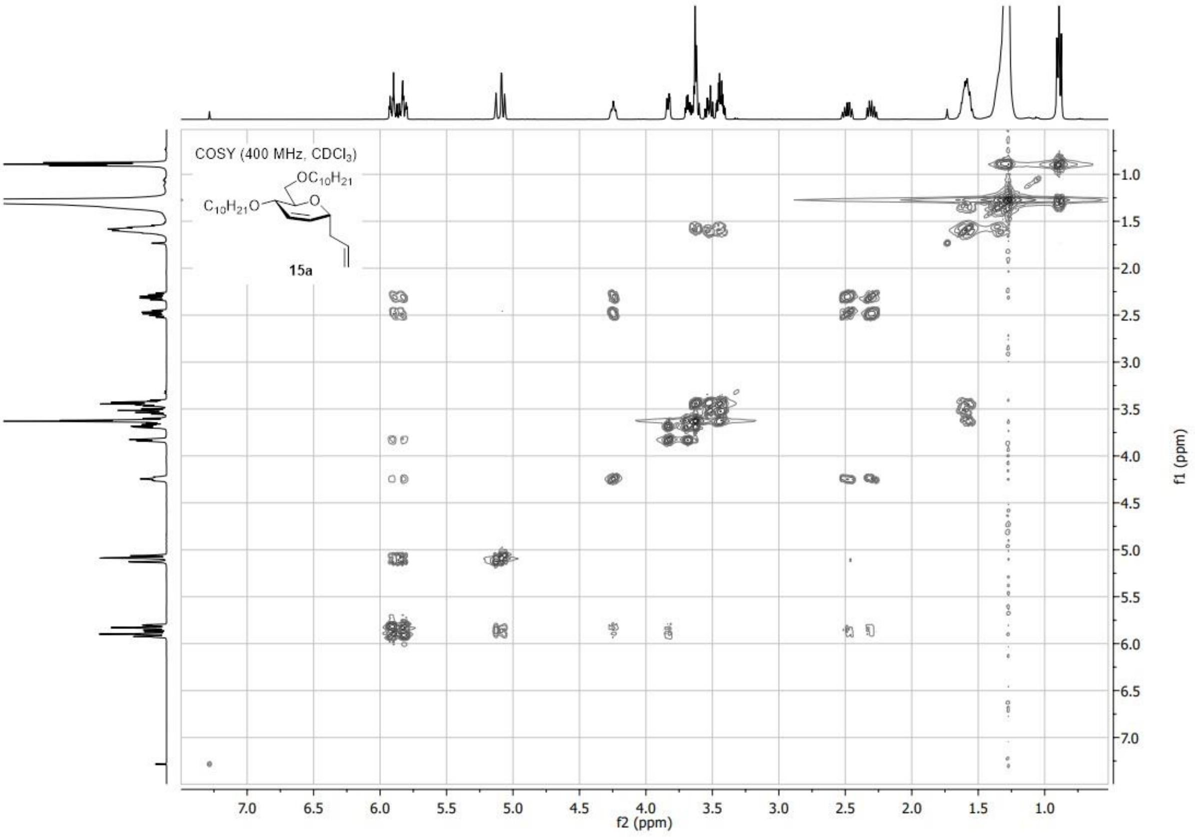
COSY (400 MHz, CDCl3)
OC10H21
C10H21O
O
15a
f2 (ppm)
f1 (ppm)

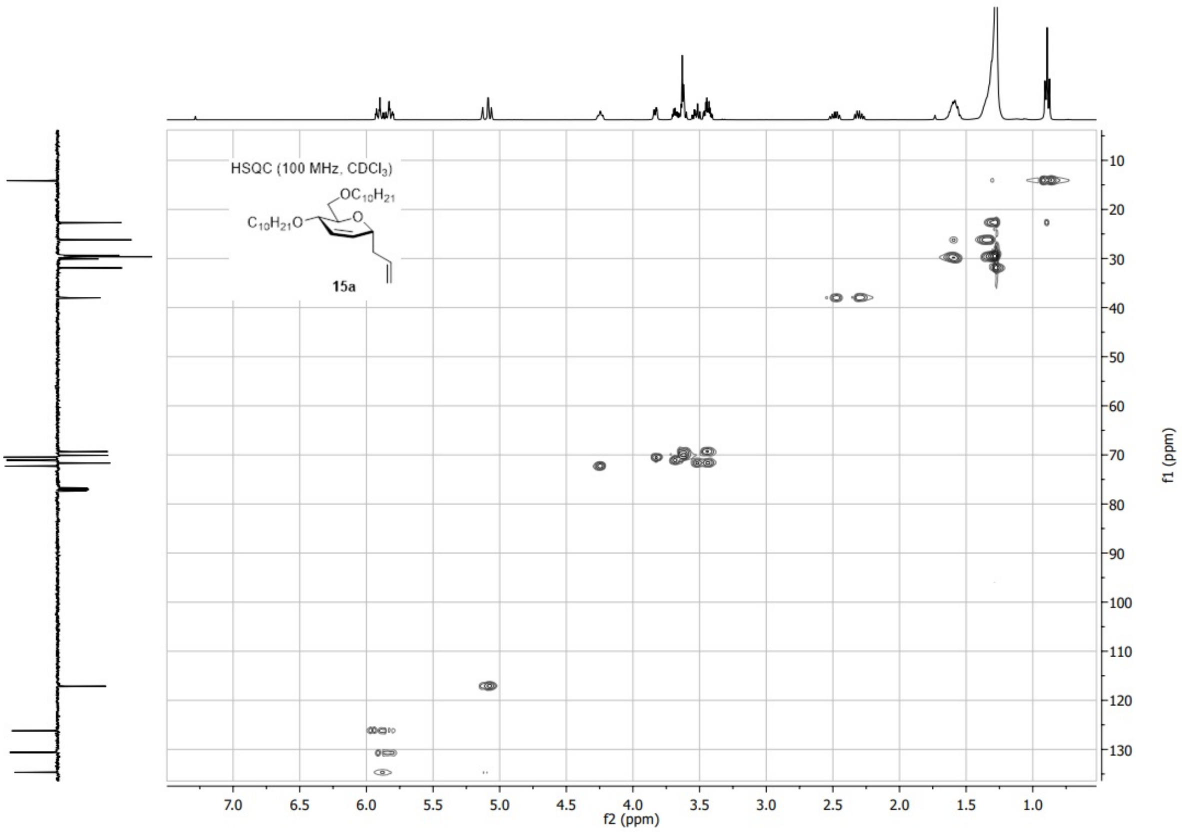
HSQC (100 MHz, CDCl3)
OC10H21
C10H21O
O
15a
f2 (ppm)
f1 (ppm)

NMR spectra of compound **15b**

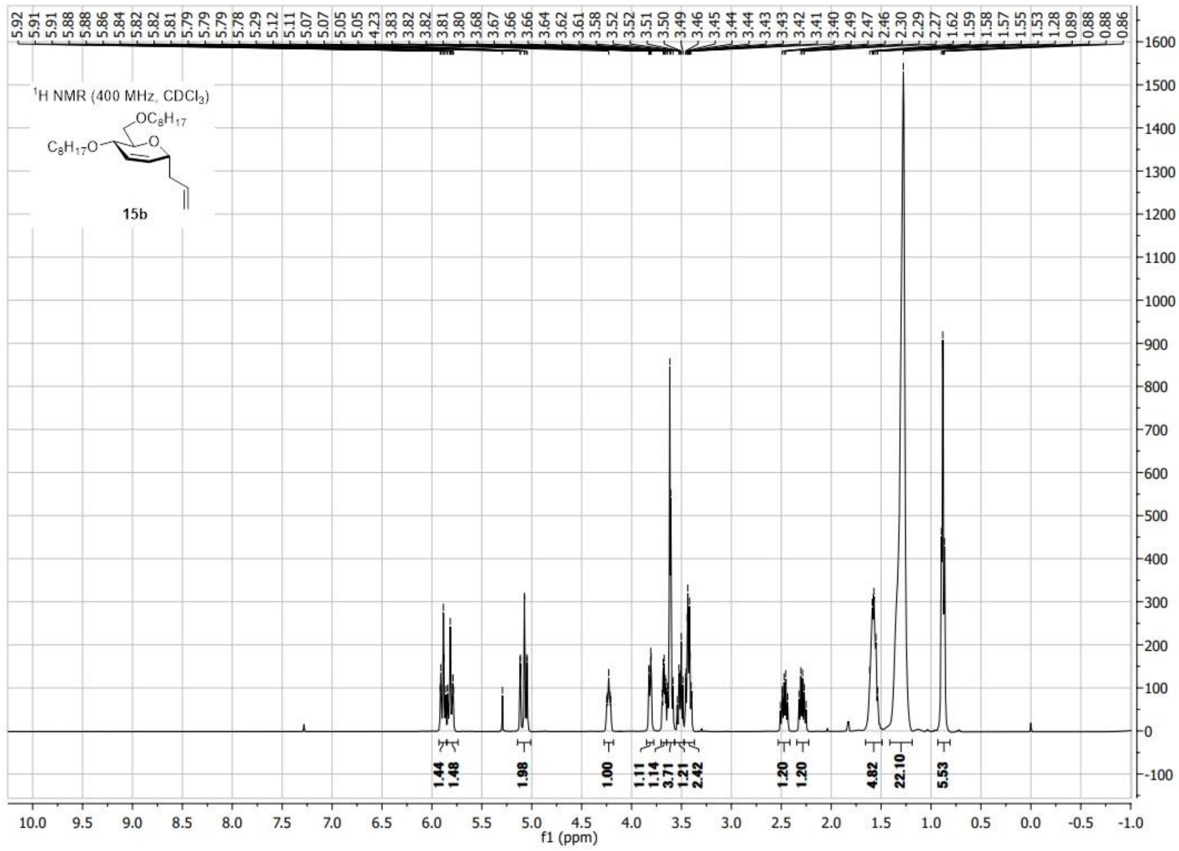

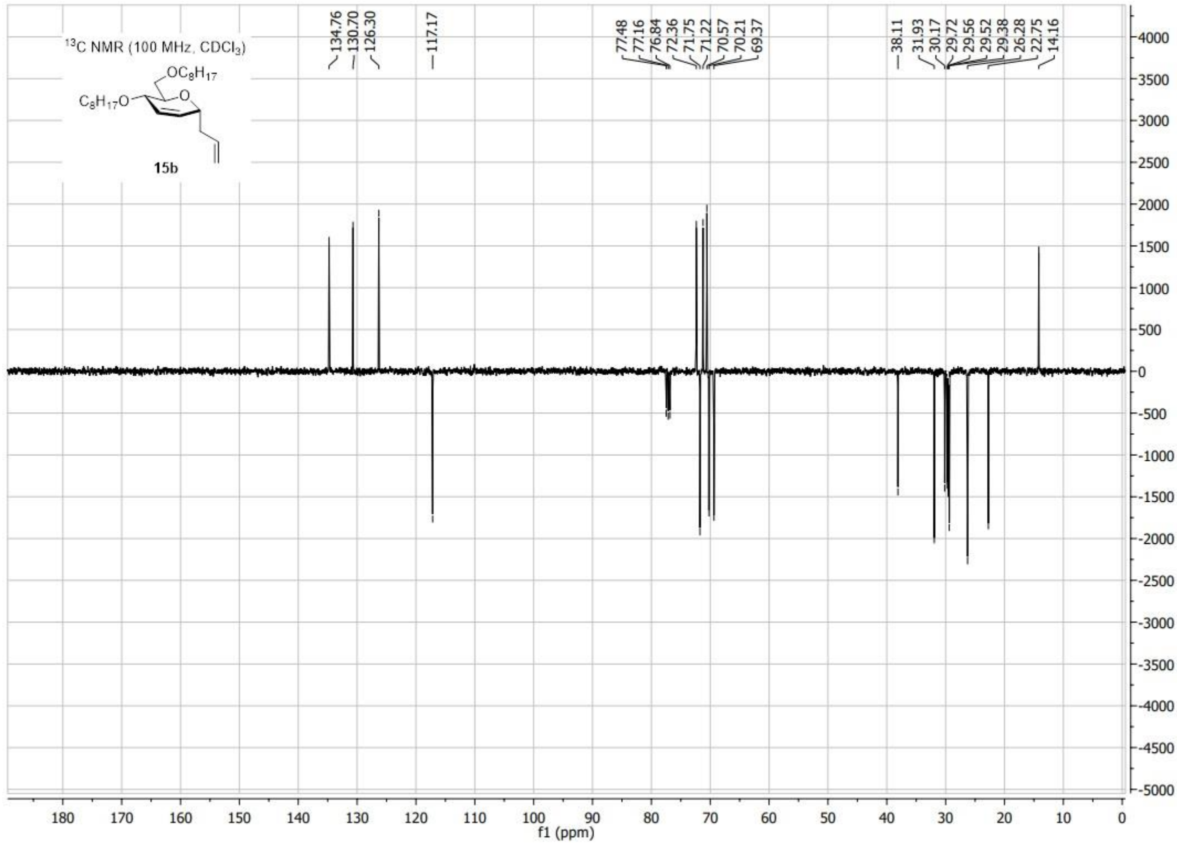

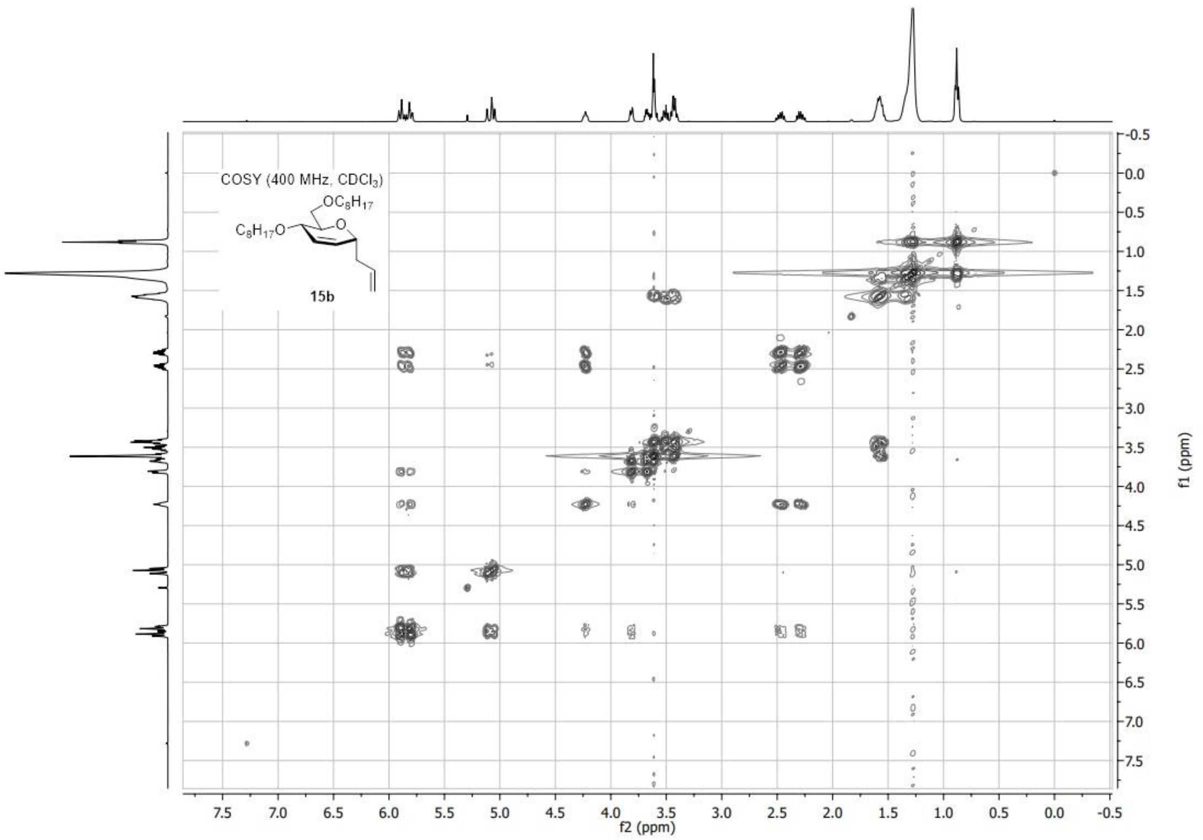
COSY (400 MHz, CDCl3)
OC8H17
C8H17O
O
15b
f2 (ppm)
f1 (ppm)

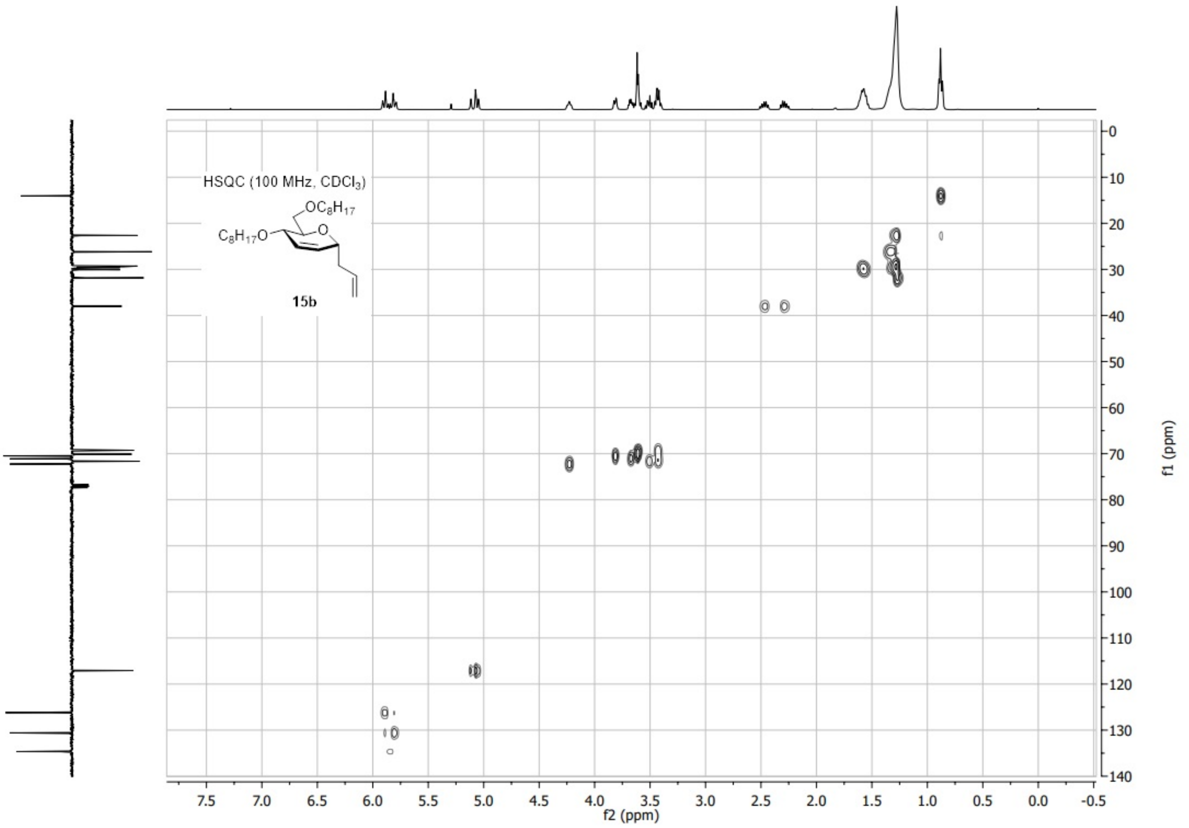
HSQC (100 MHz, CDCl3)
OC8H17
C8H17O
O
15b
f2 (ppm)
f1 (ppm)

NMR spectra of compound **15c**

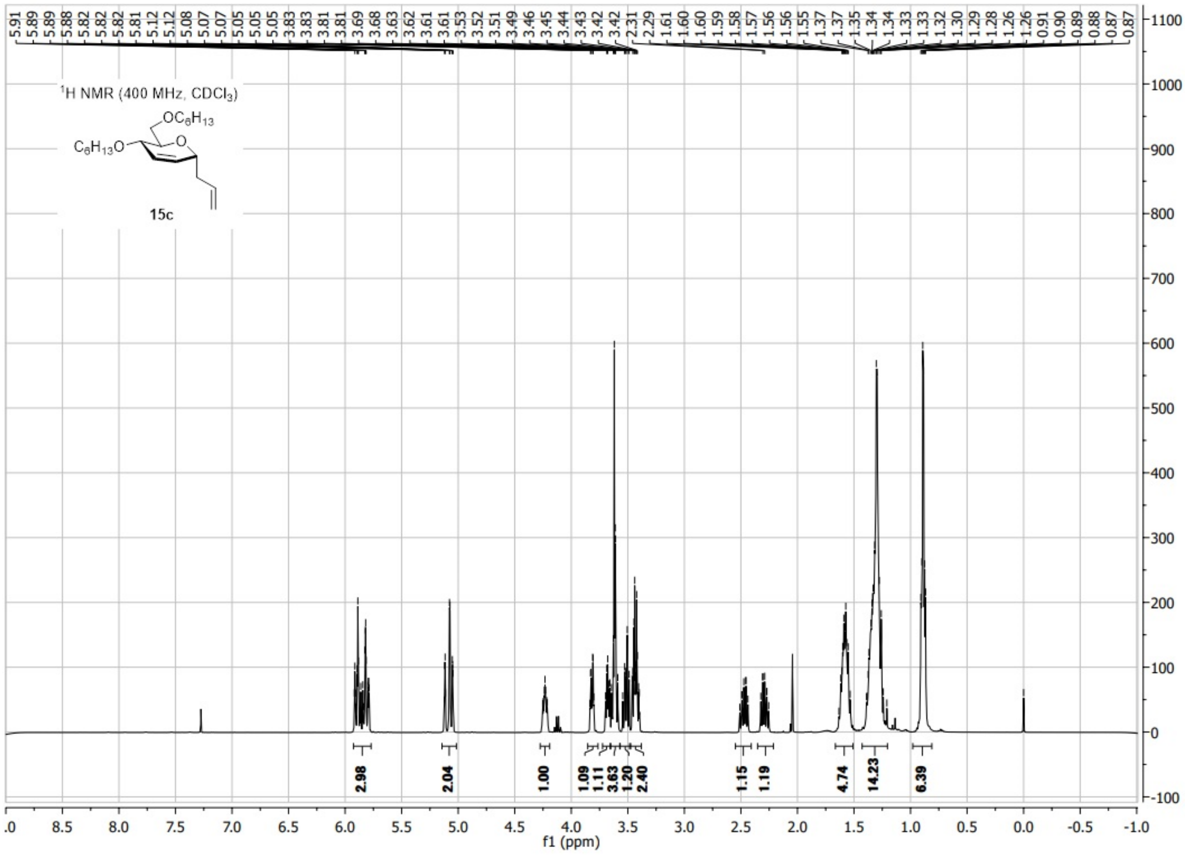

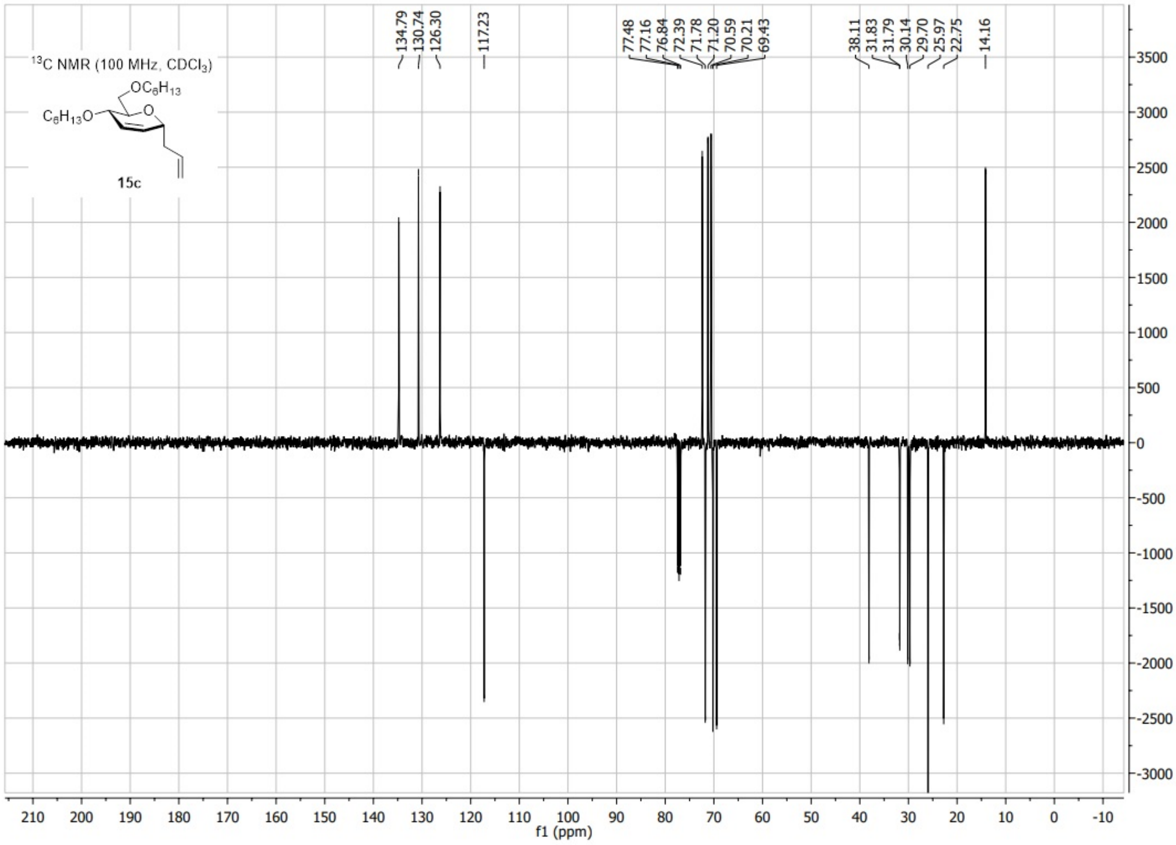

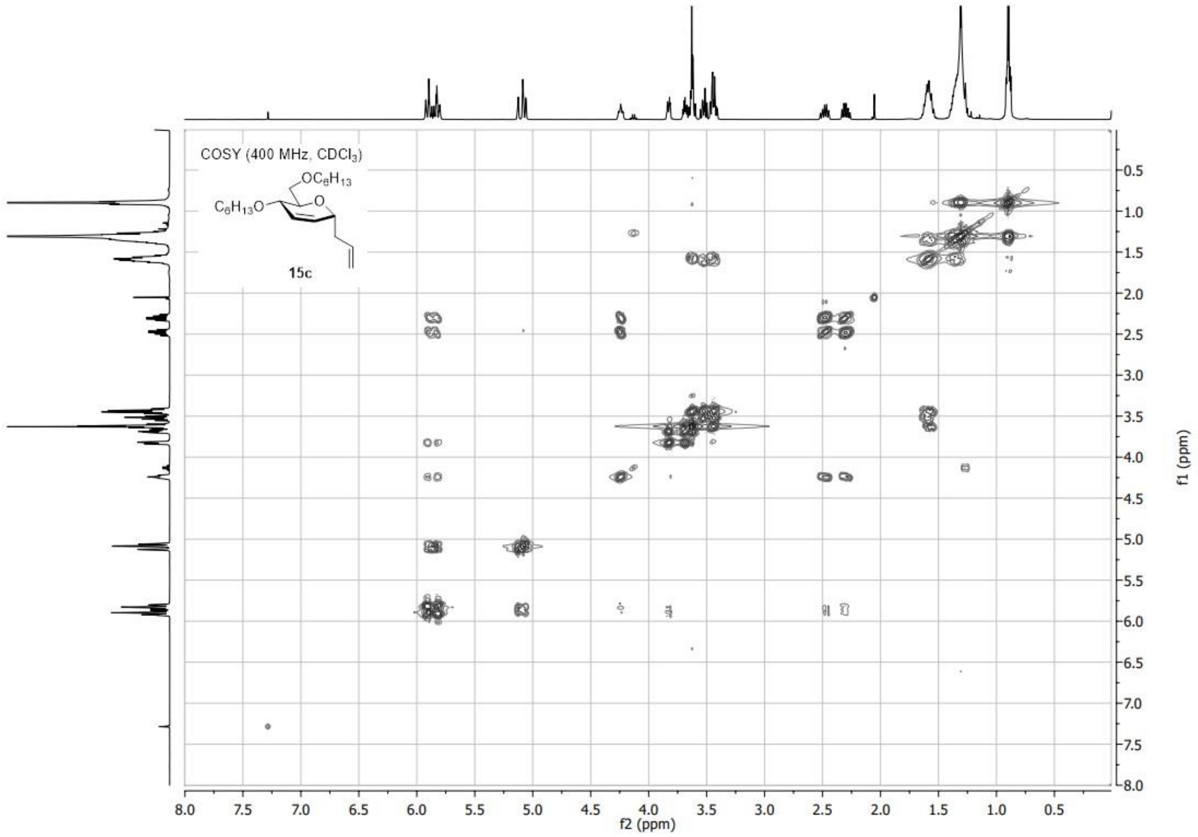
COSY (400 MHz, $CDCl_3$)
OC_6H_{13}
$C_6H_{13}O$
O
15c
f2 (ppm)
f1 (ppm)

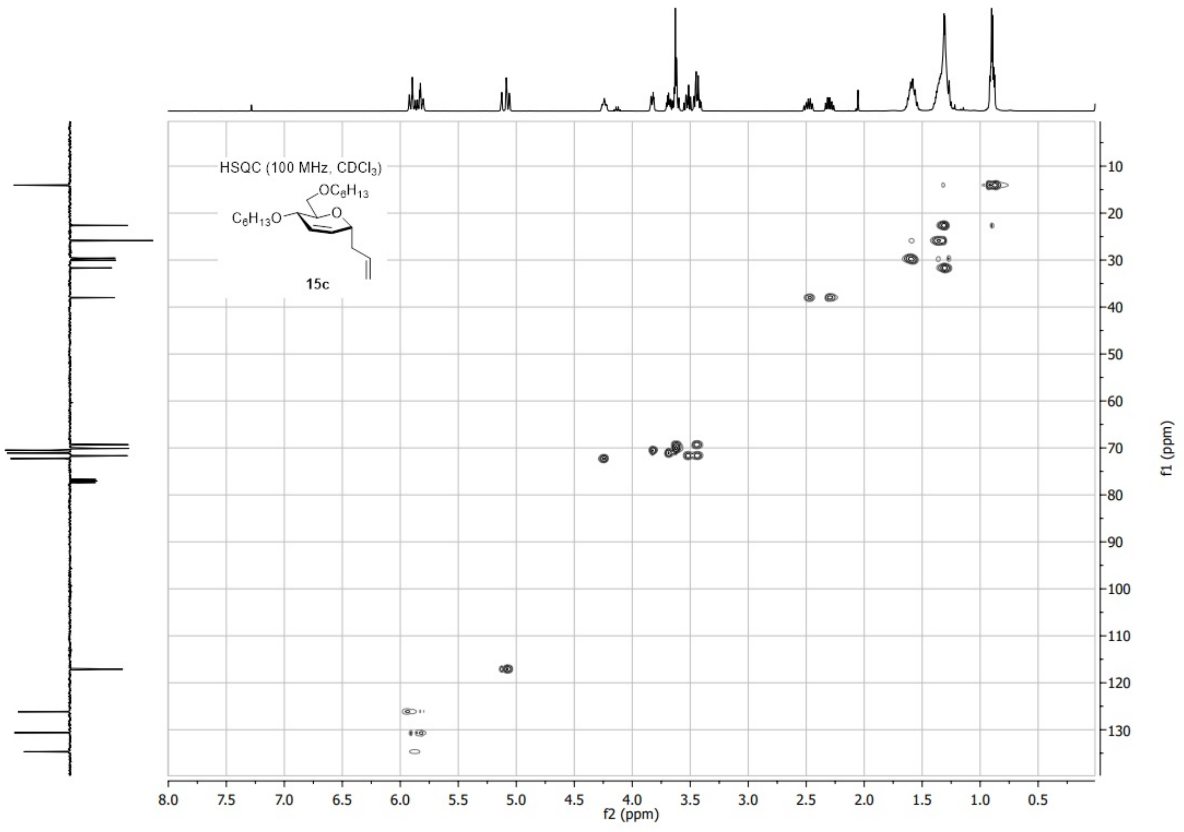
HSQC (100 MHz, $CDCl_3$)
OC_6H_{13}
$C_6H_{13}O$
O
15c
f2 (ppm)
f1 (ppm)

NMR spectra of compound **17a**

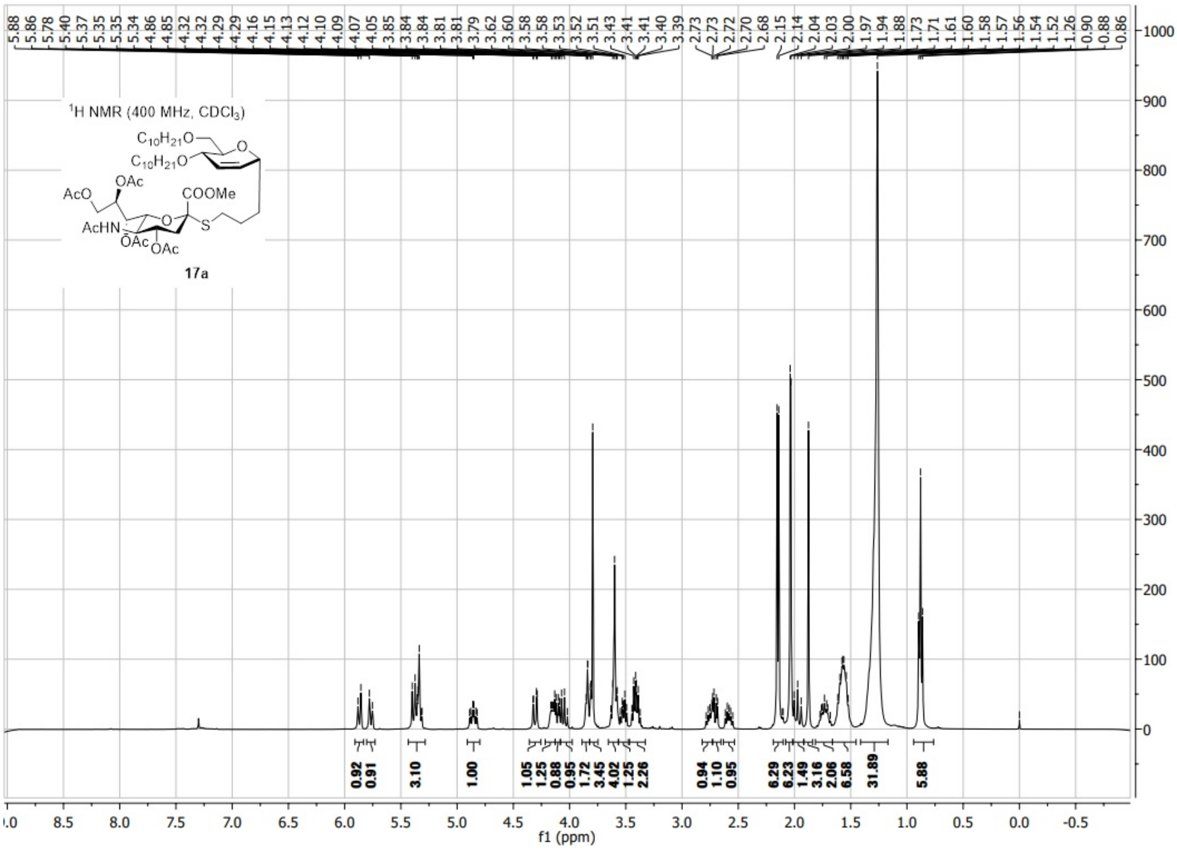

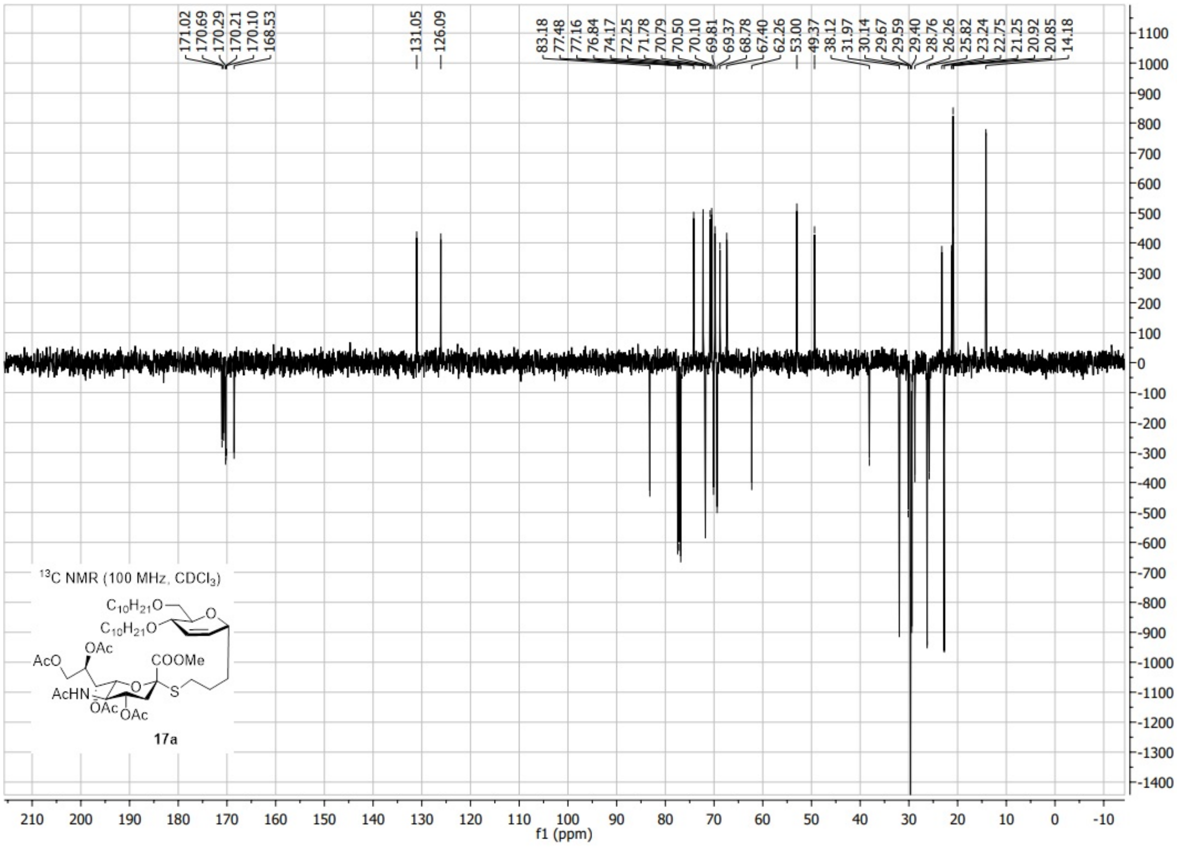

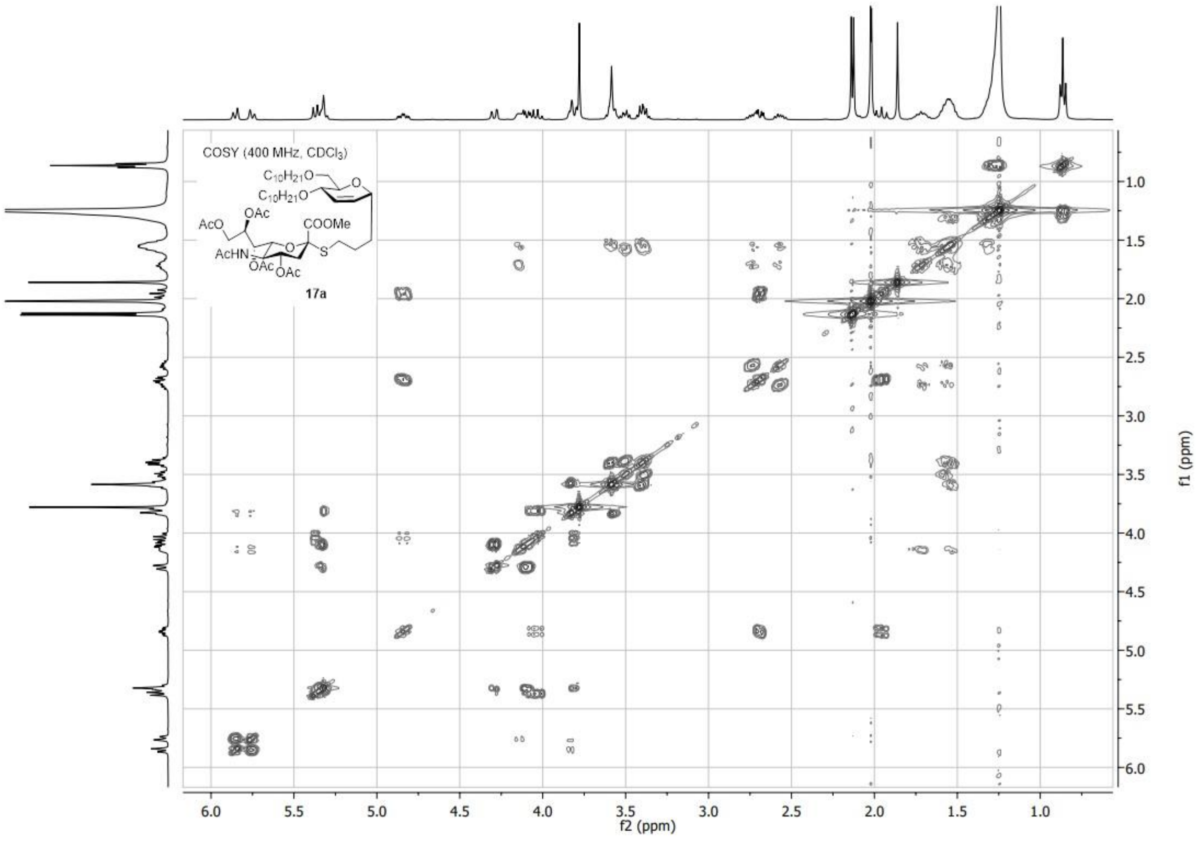
COSY (400 MHz, CDCl3)
17a
f2 (ppm)
f1 (ppm)

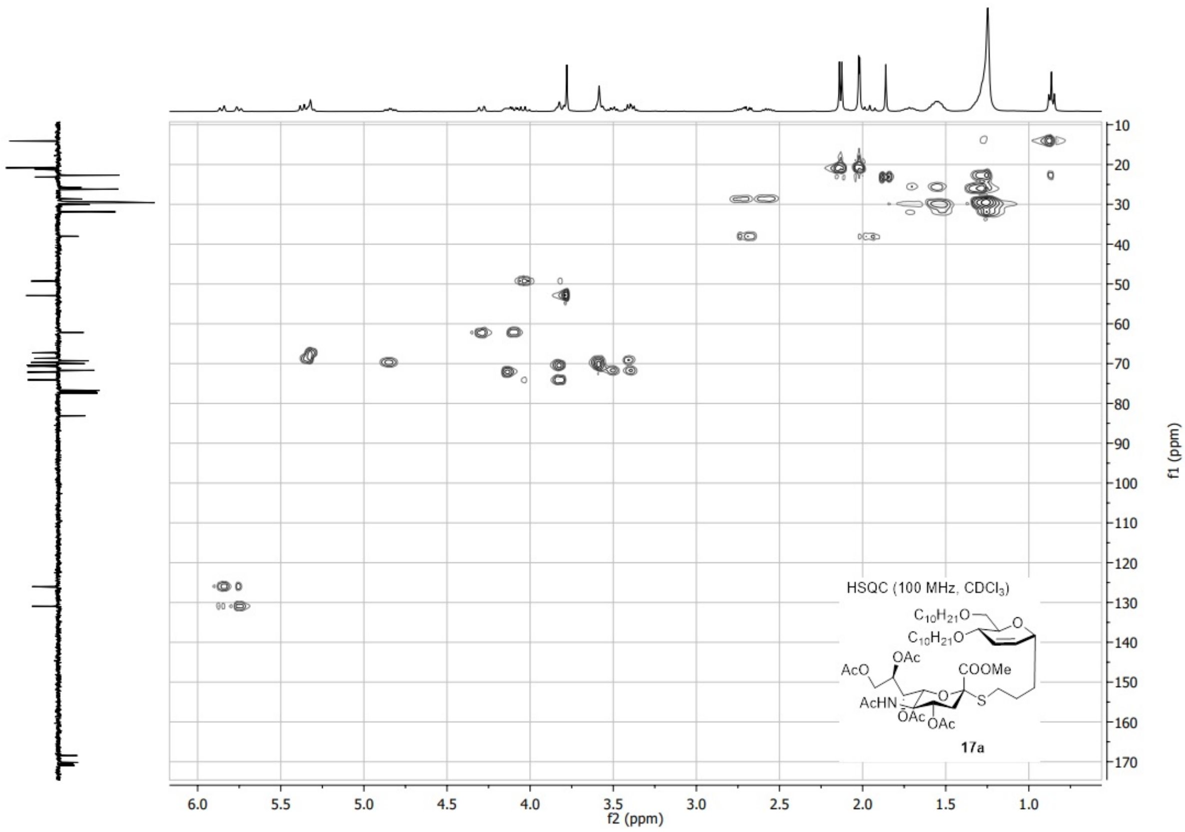
HSQC (100 MHz, CDCl3)
17a
f2 (ppm)
f1 (ppm)

NMR spectra of compound **17b**

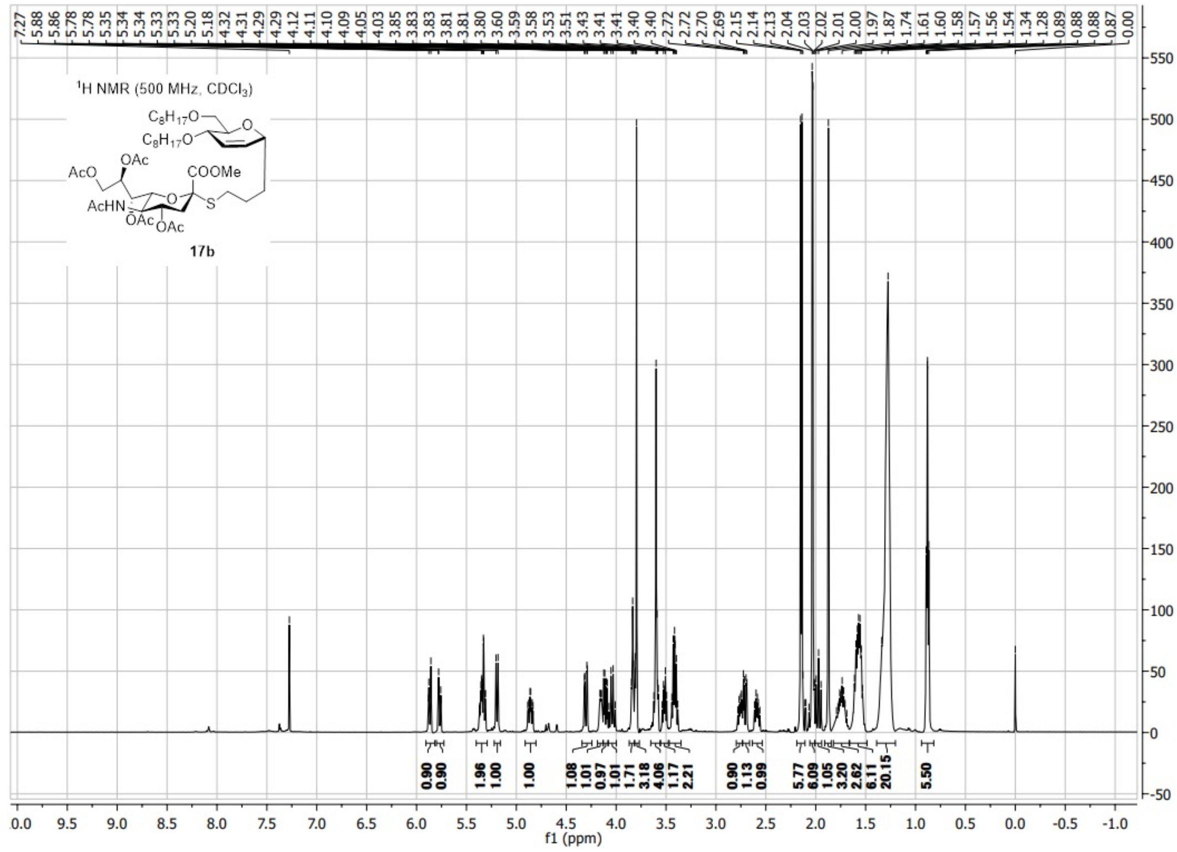

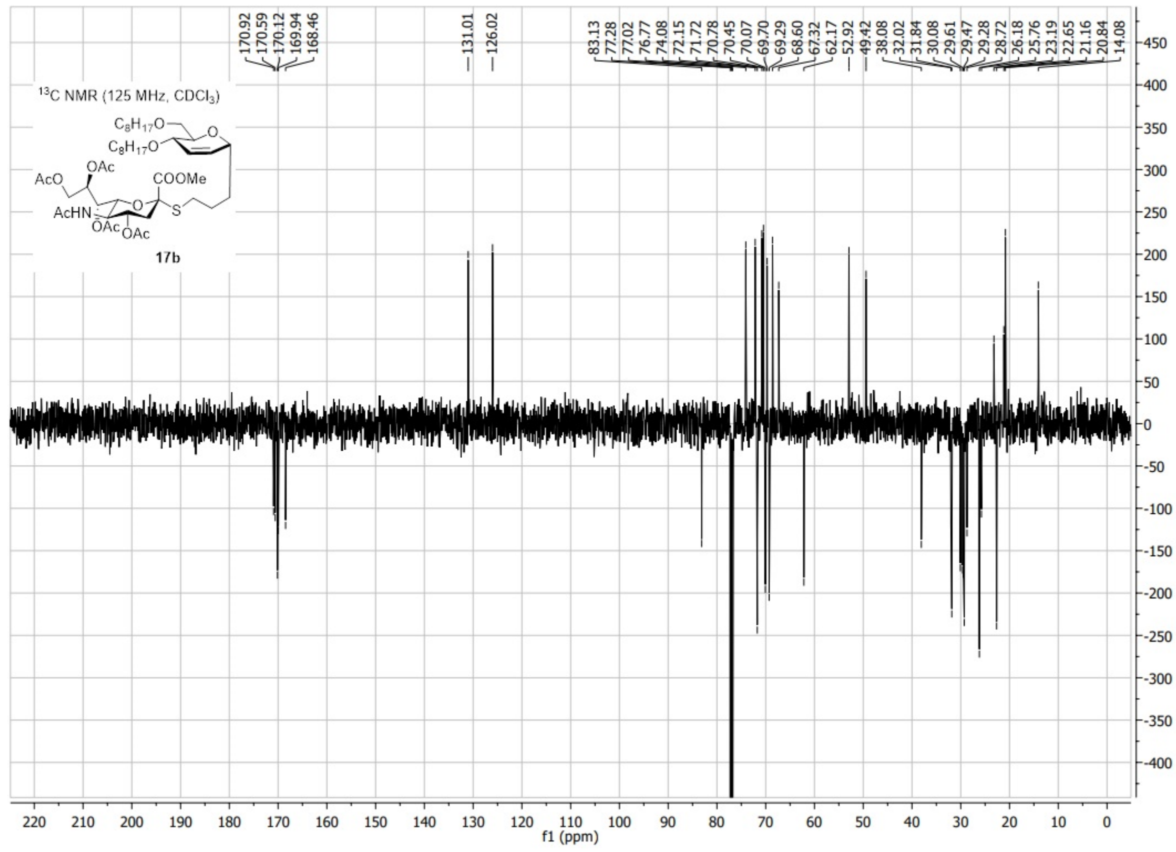

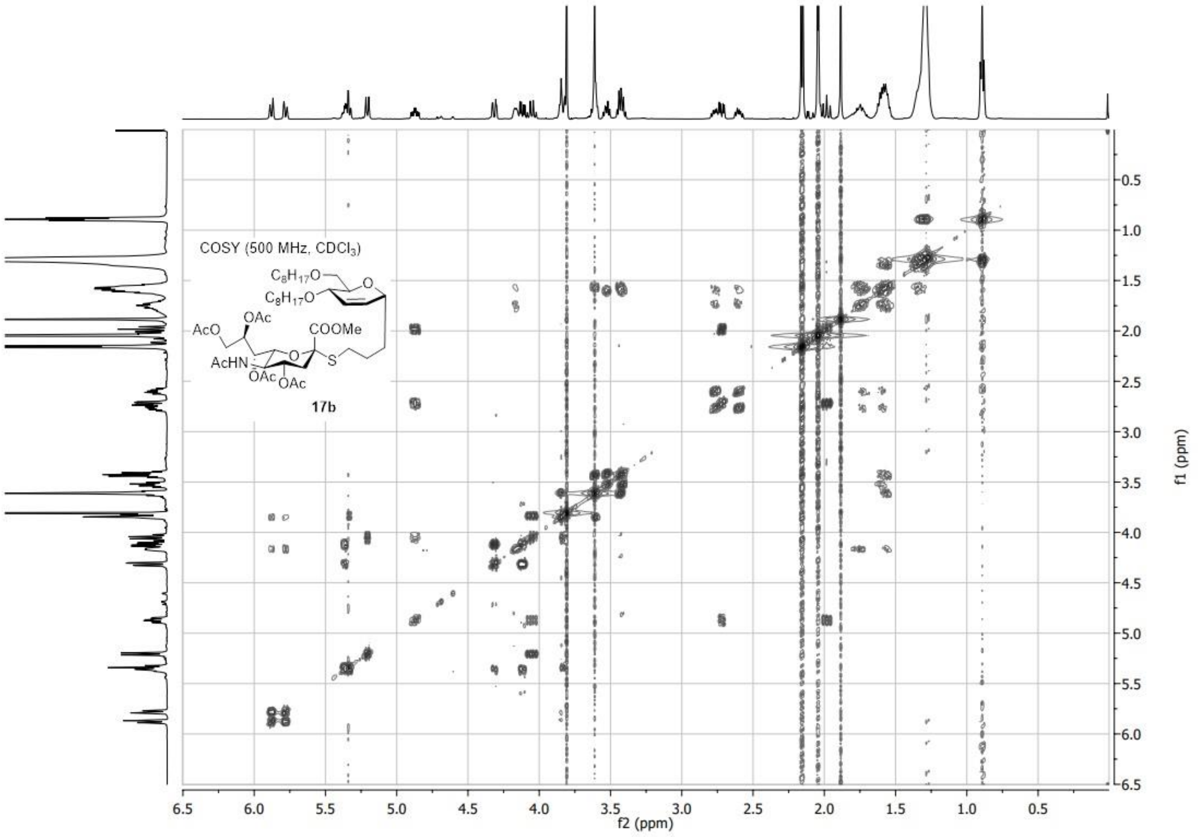
COSY (500 MHz, CDCl3)
17b
f2 (ppm)
f1 (ppm)

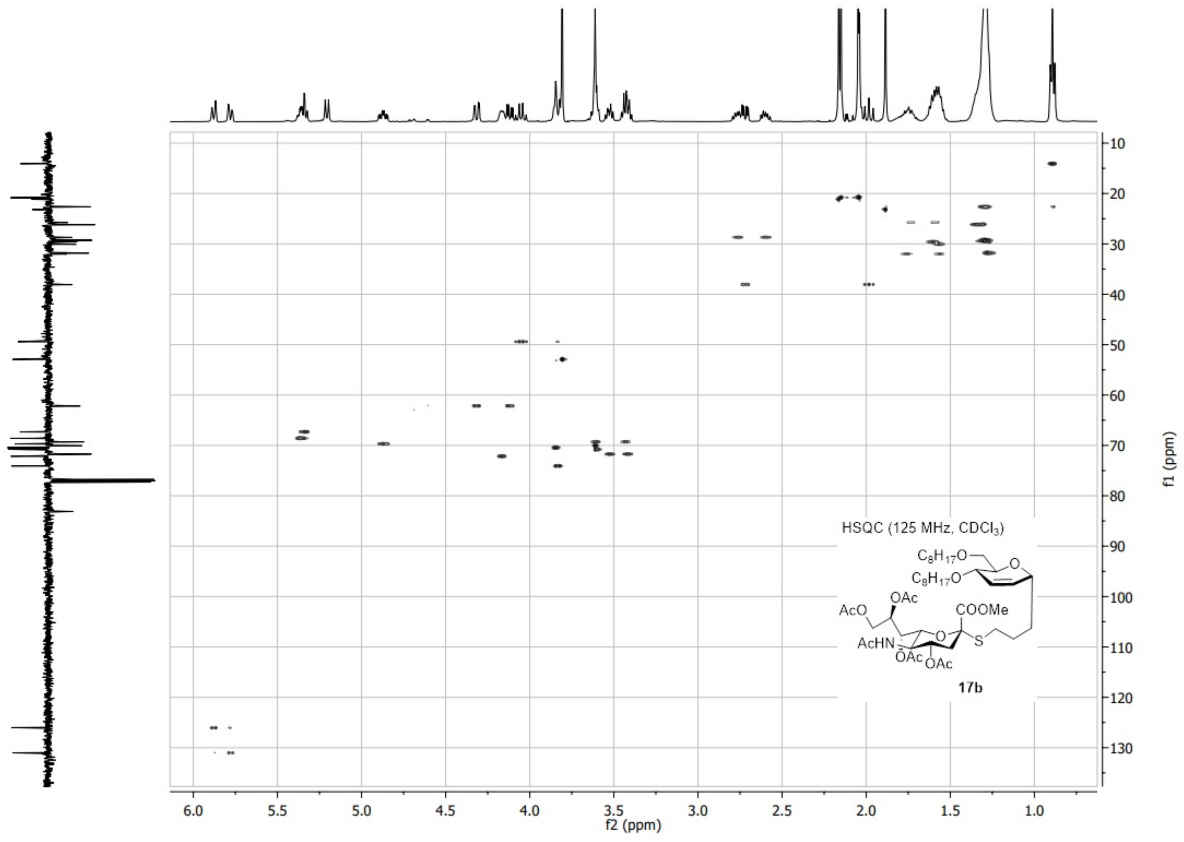
HSQC (125 MHz, CDCl3)
17b
f2 (ppm)
f1 (ppm)

NMR spectra of compound **17c**

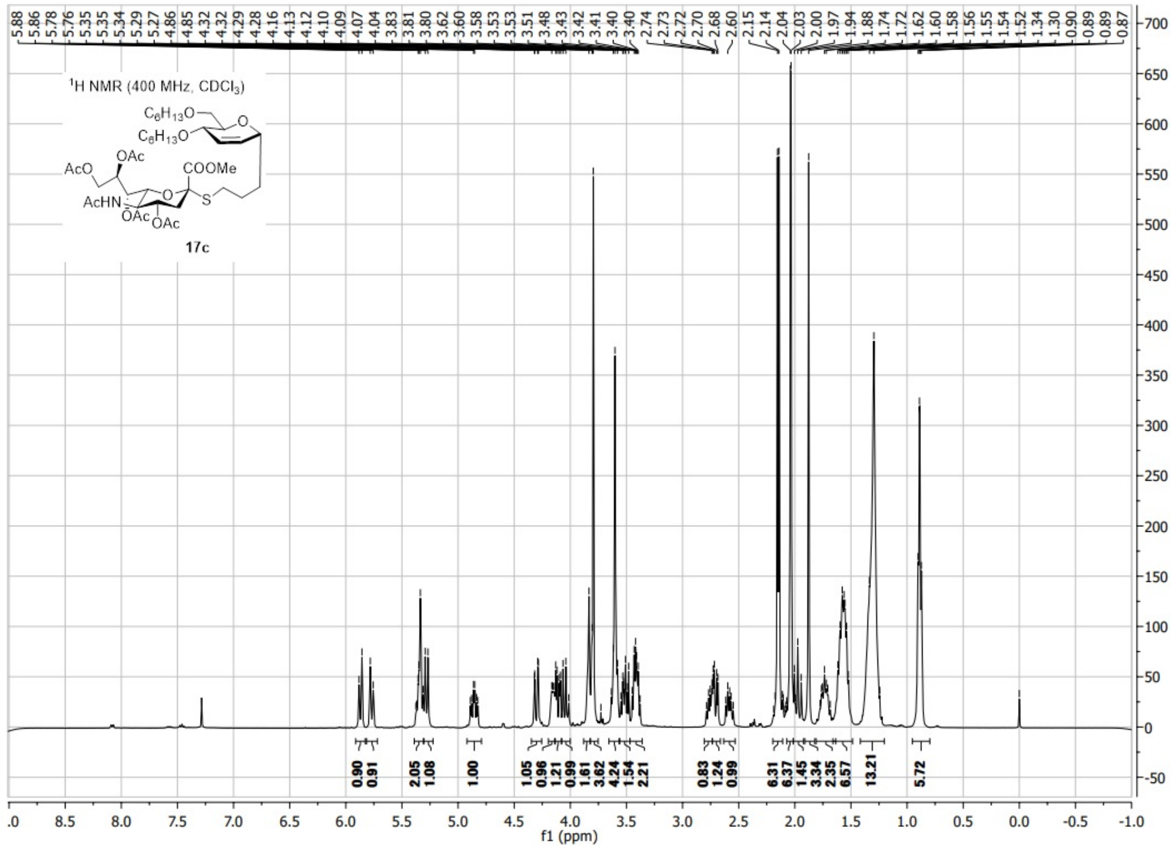

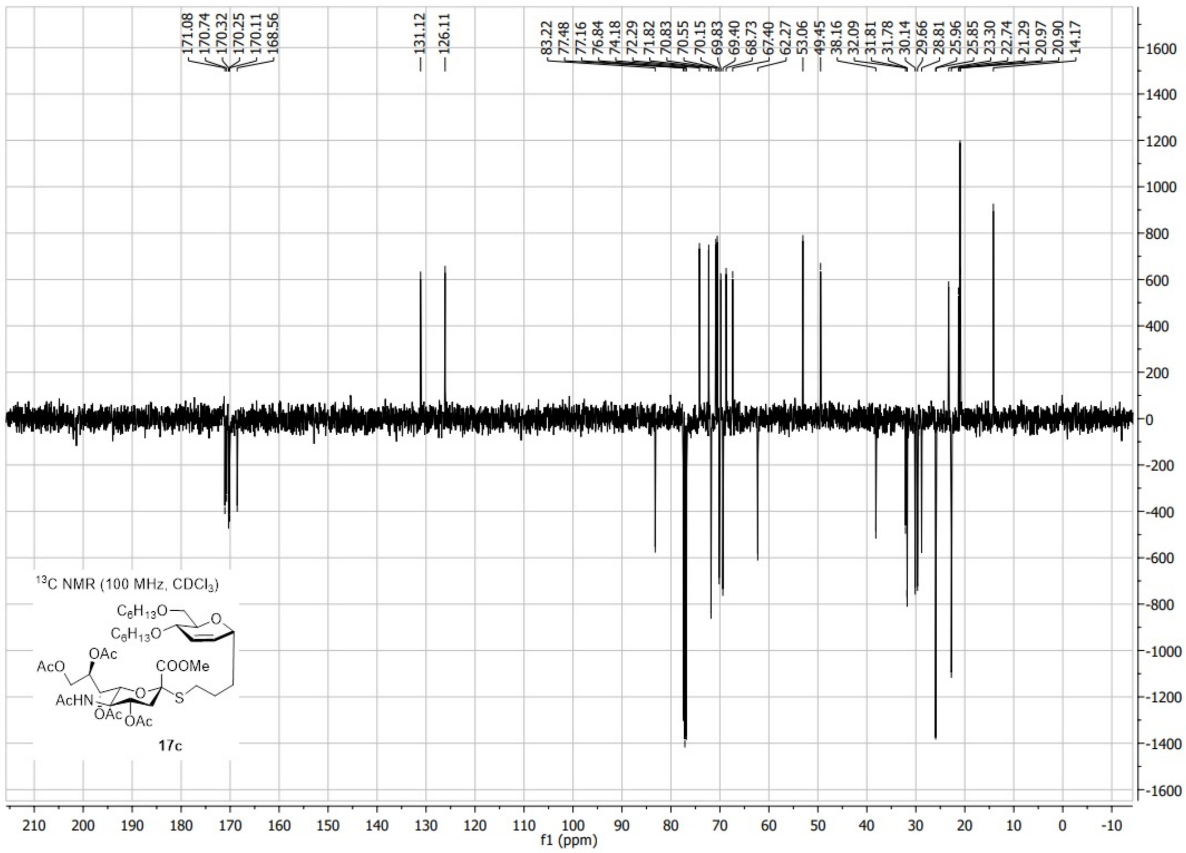

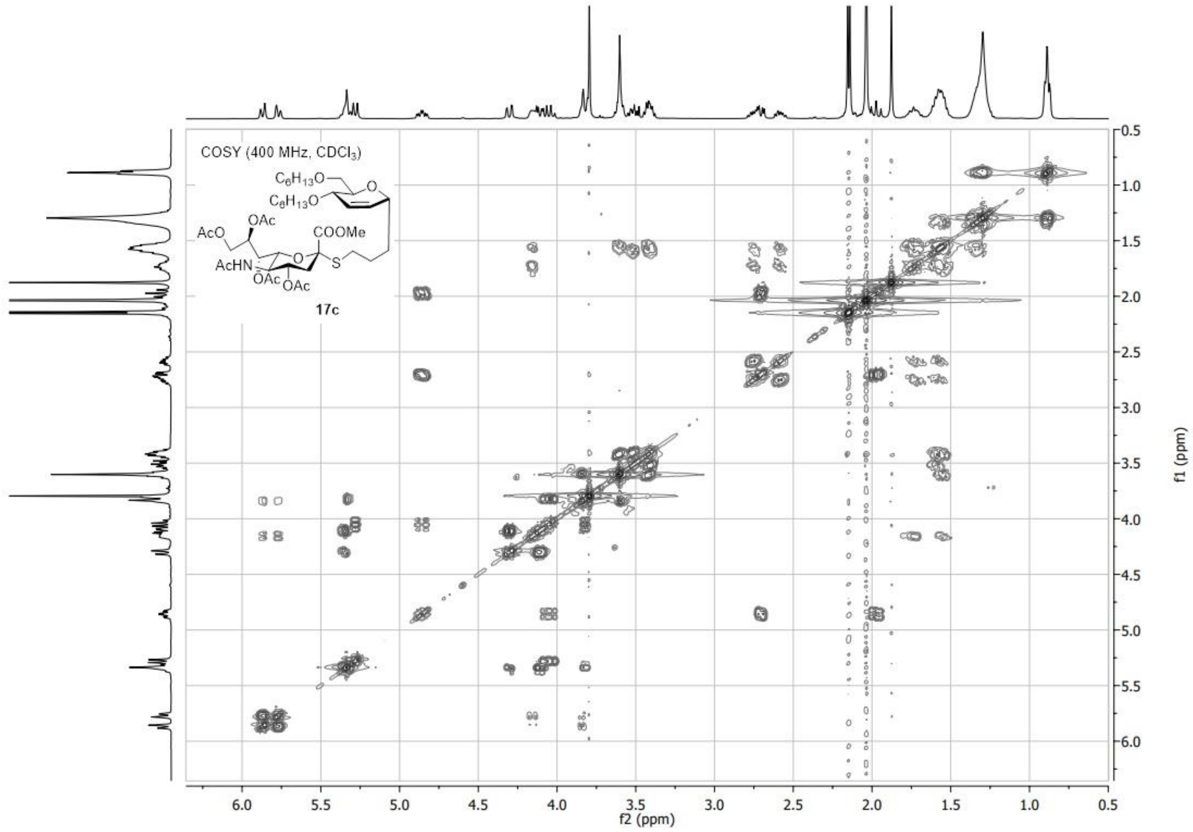
COSY (400 MHz, CDCl$_3$)
$C_6H_{13}O$
$C_6H_{13}O$
AcO
OAc
COOMe
AcHN
OAc
OAc
S
17c
f2 (ppm)
f1 (ppm)

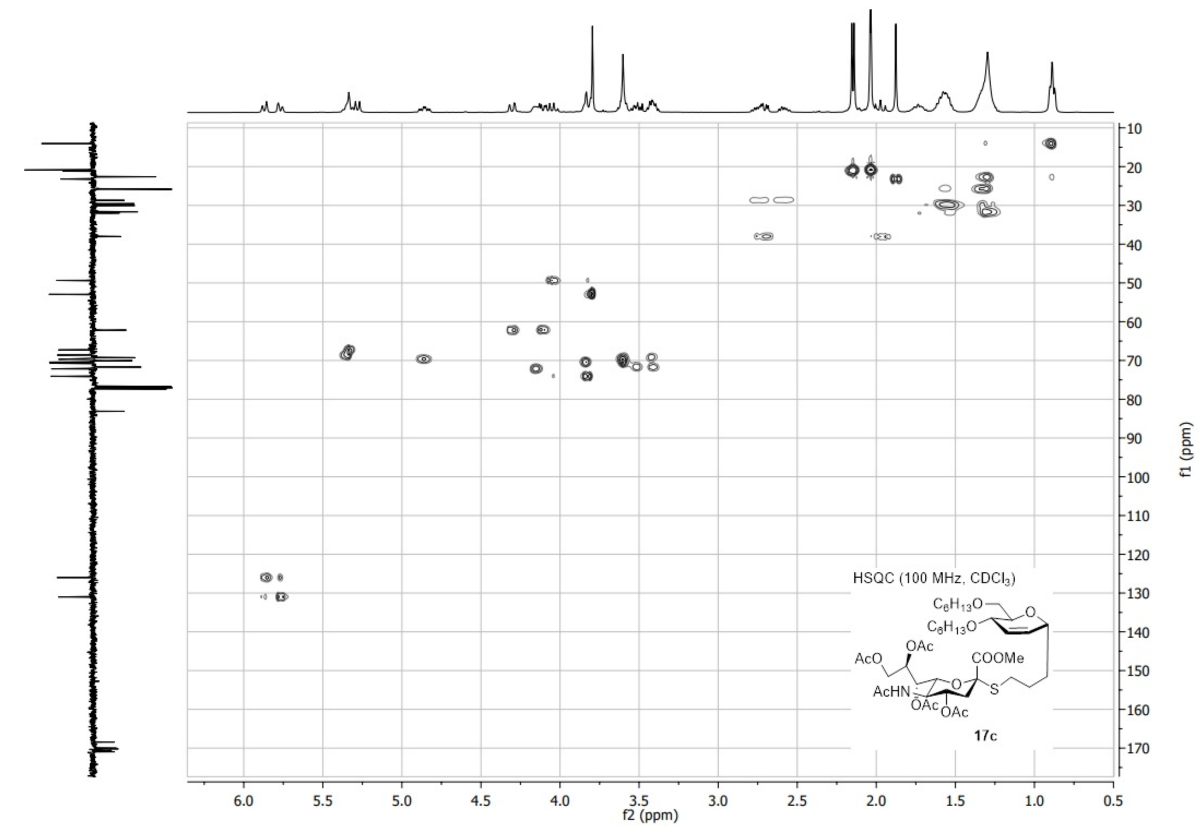
HSQC (100 MHz, CDCl$_3$)
$C_6H_{13}O$
$C_6H_{13}O$
AcO
OAc
COOMe
AcHN
OAc
OAc
S
17c
f2 (ppm)
f1 (ppm)

NMR spectra of compound **18a**

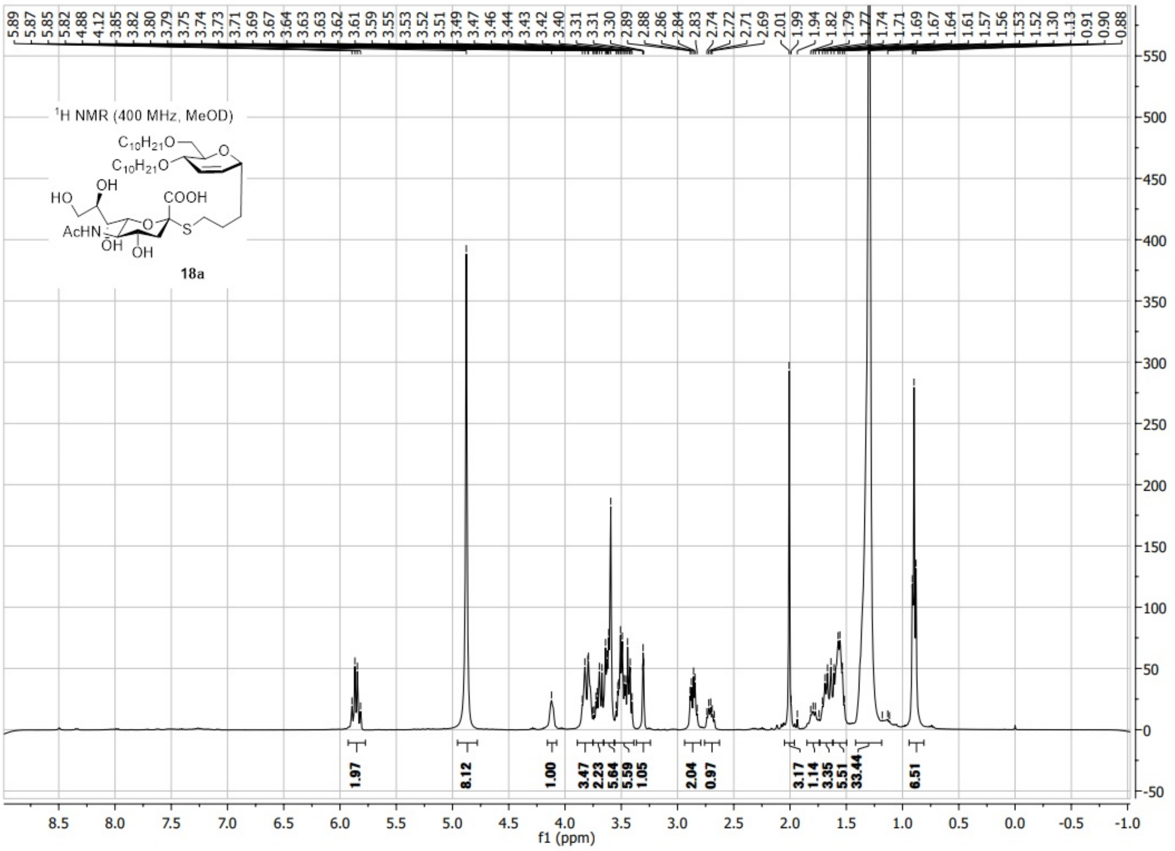

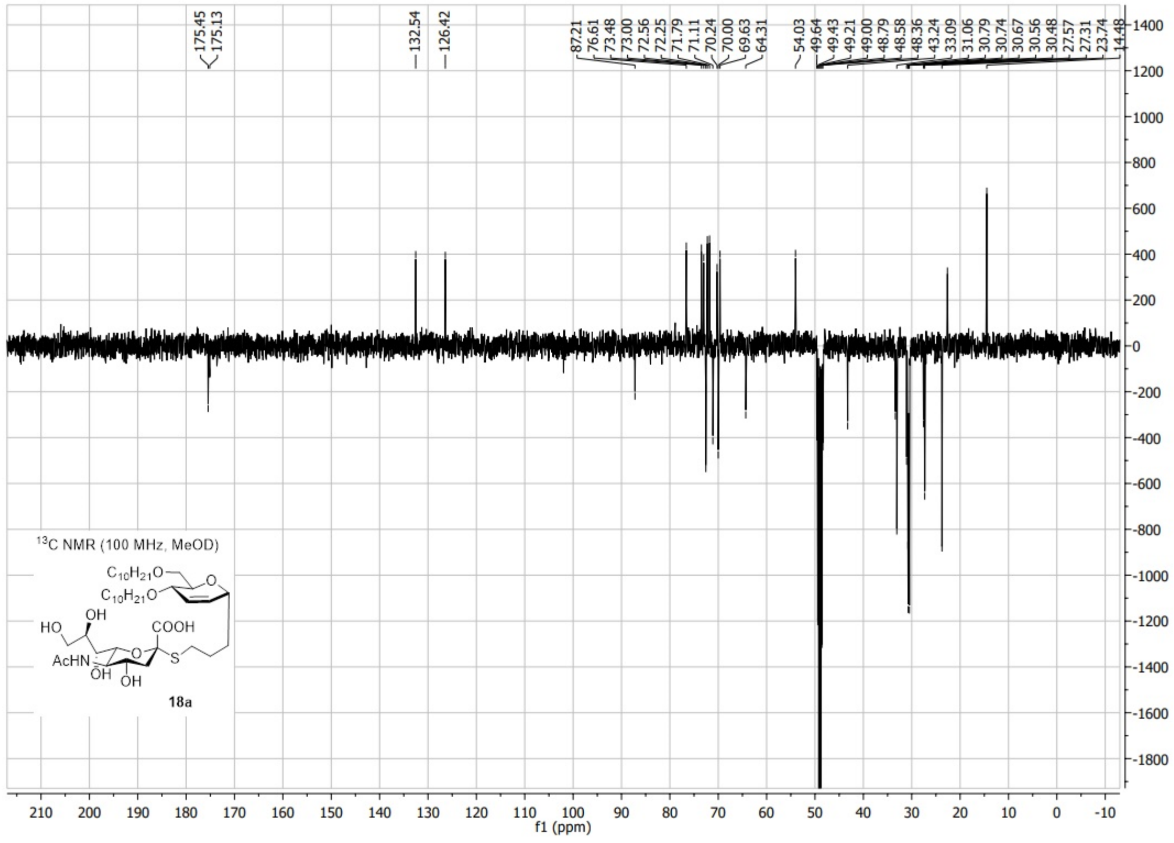

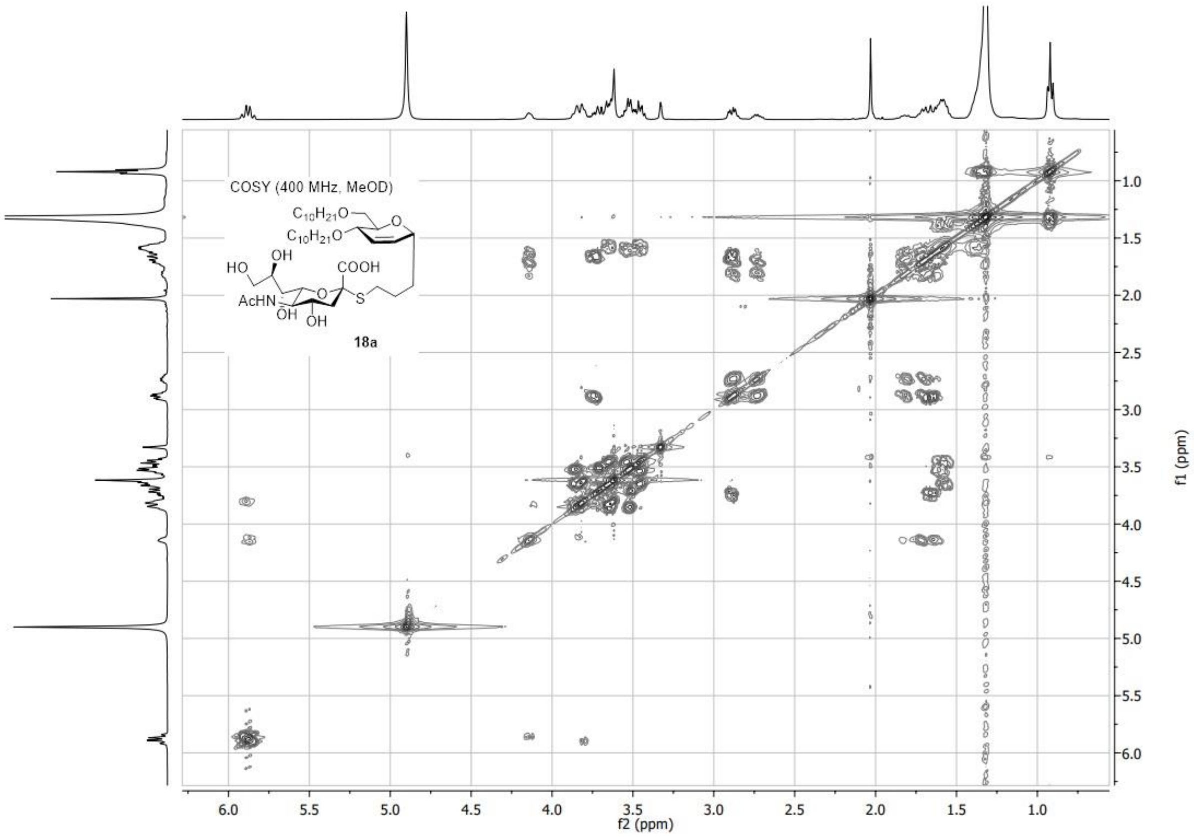
COSY (400 MHz, MeOD)
18a
f2 (ppm)
f1 (ppm)

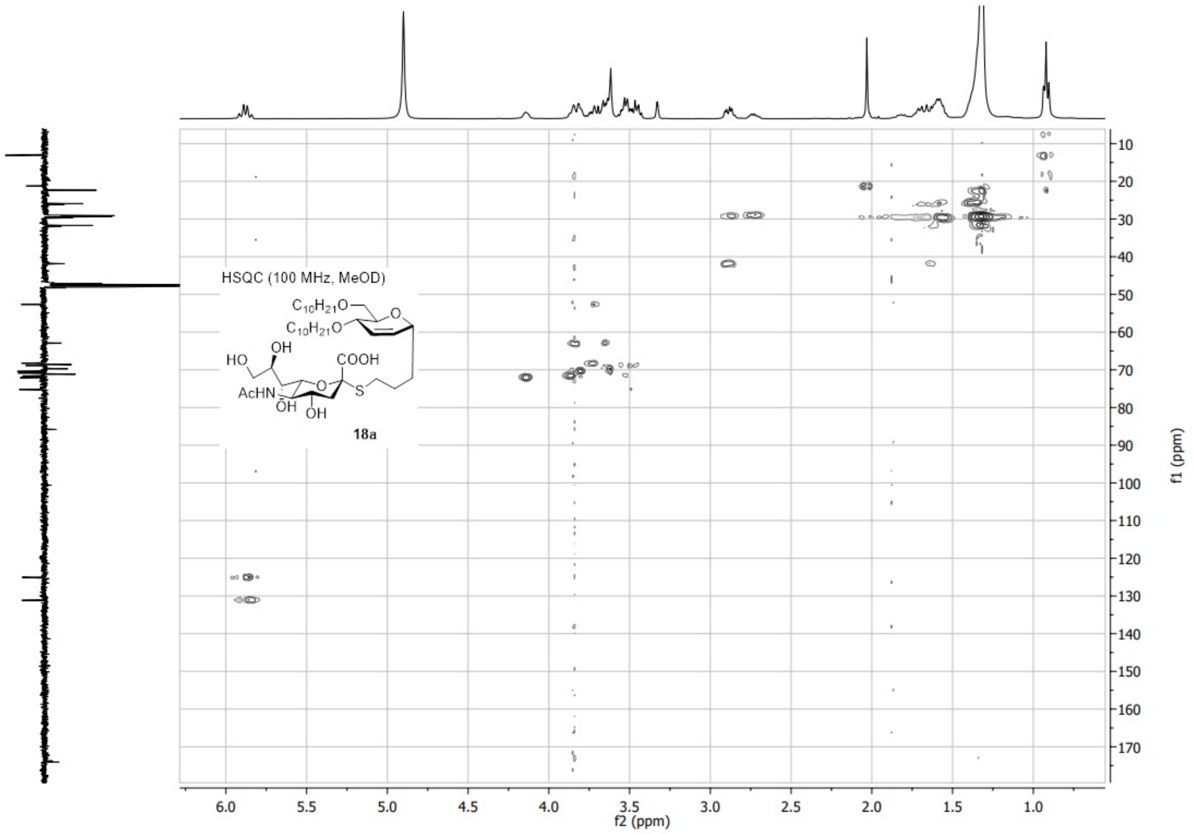
HSQC (100 MHz, MeOD)
18a
f2 (ppm)
f1 (ppm)

NMR spectra of compound **18b**

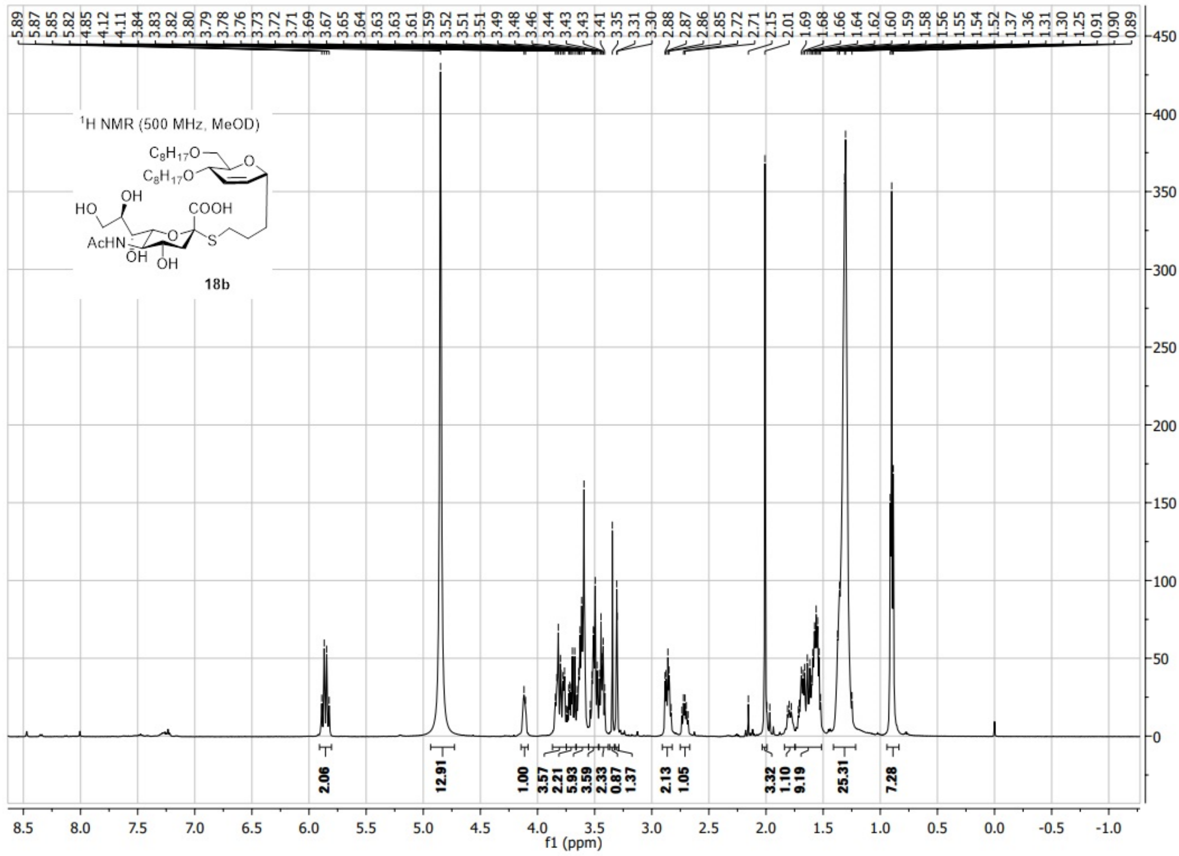

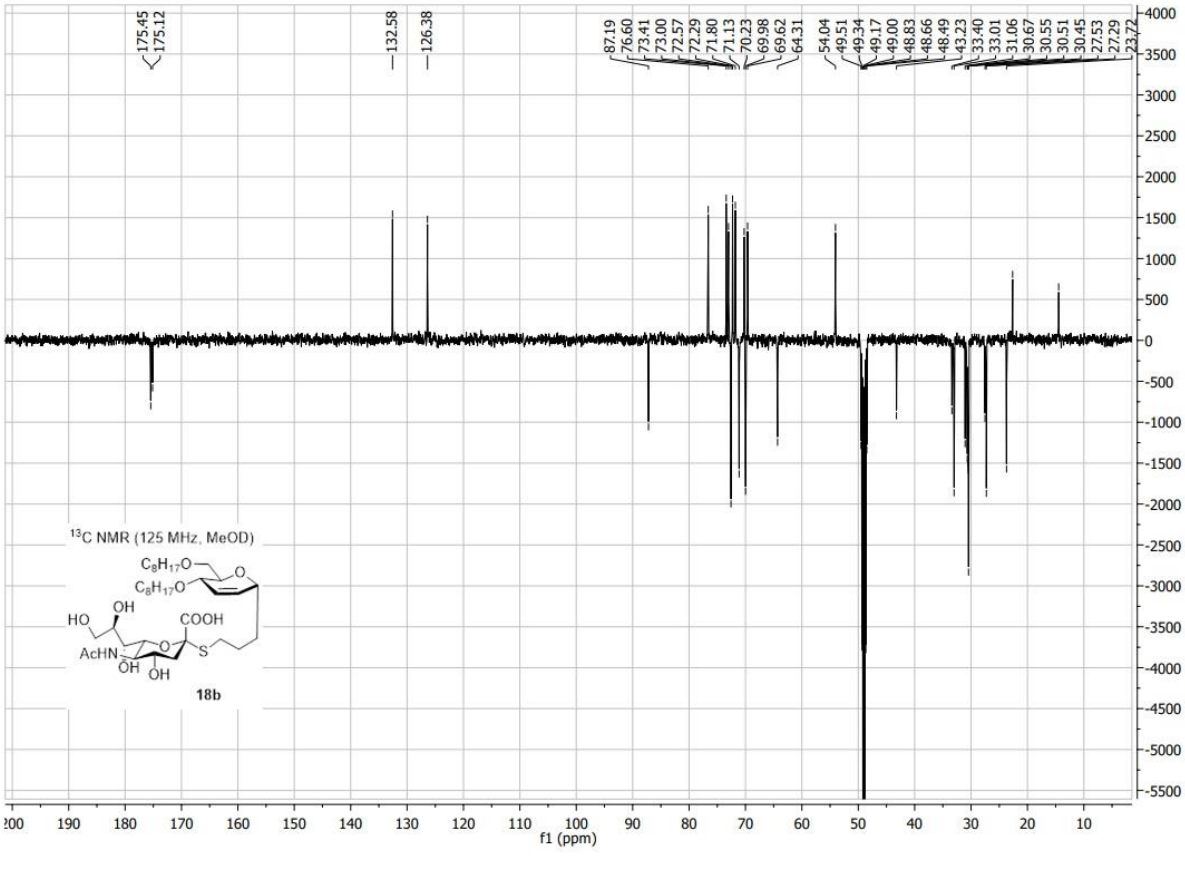

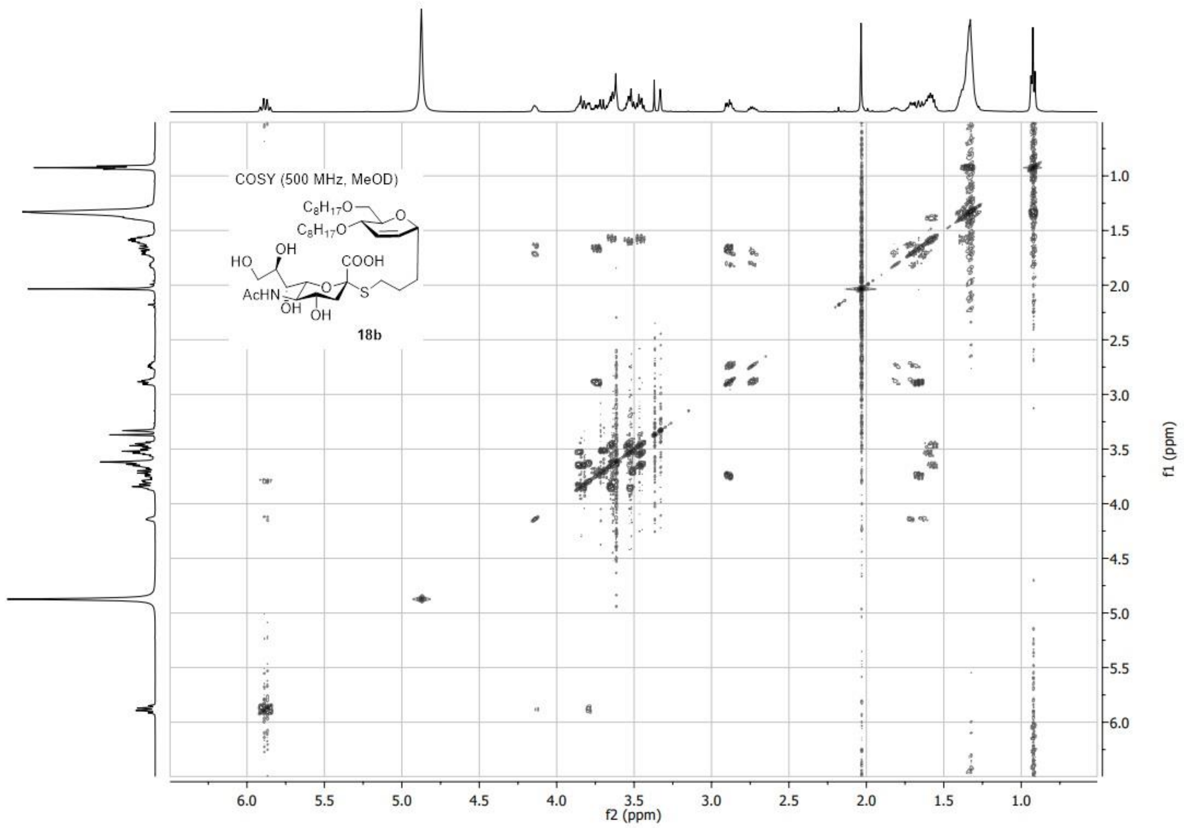
COSY (500 MHz, MeOD)
18b
f2 (ppm)
f1 (ppm)

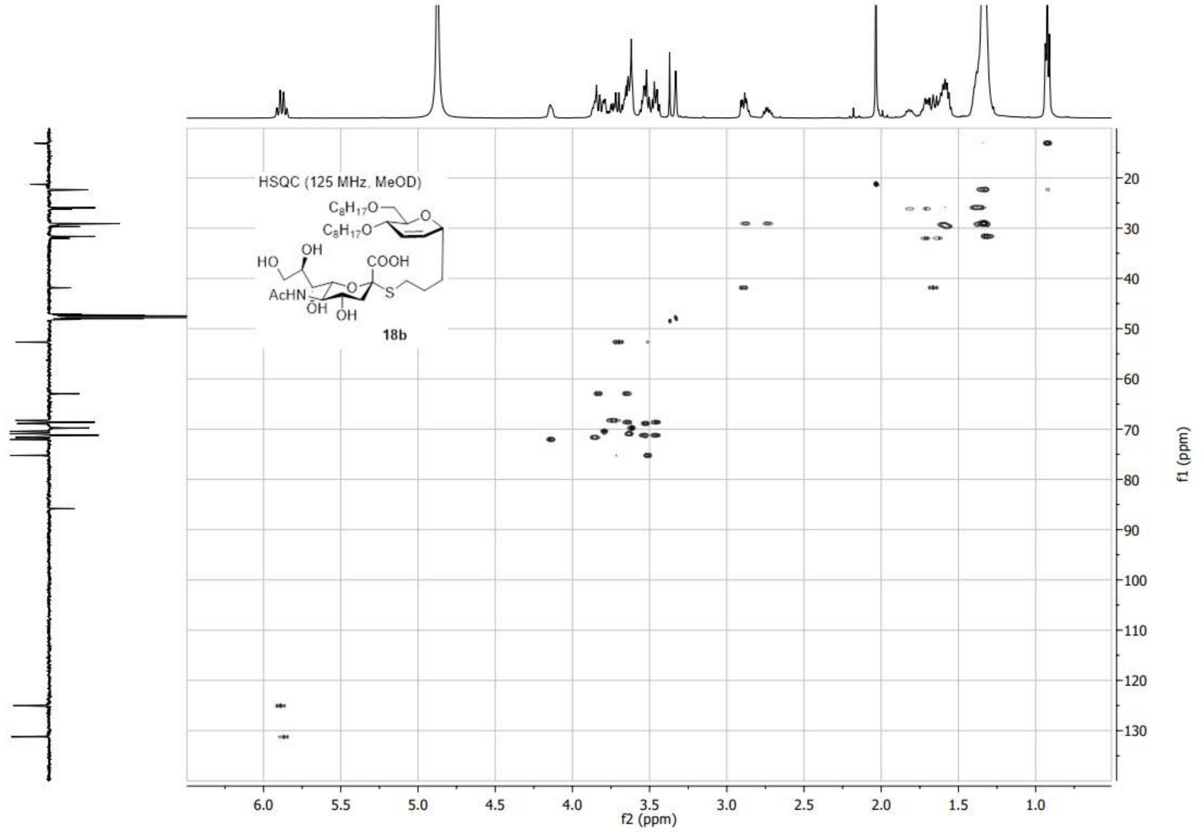
HSQC (125 MHz, MeOD)
18b
f2 (ppm)
f1 (ppm)

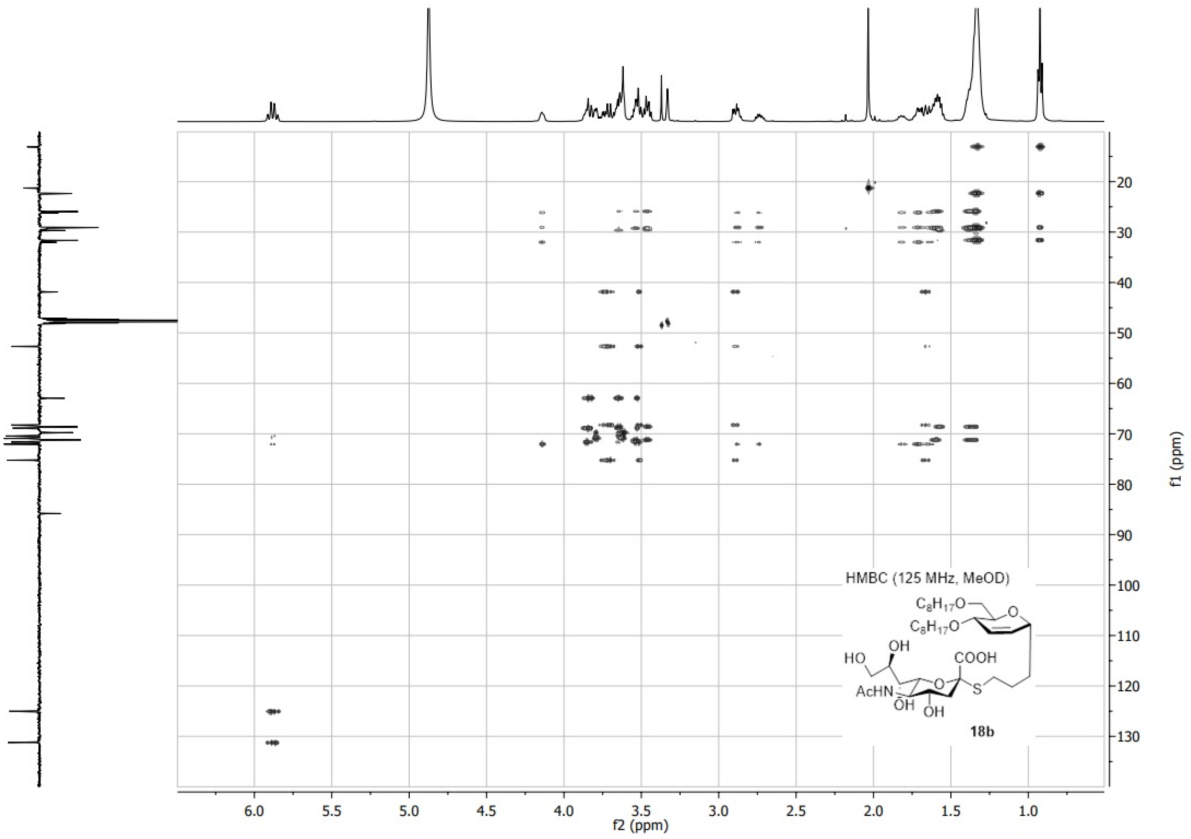
HMBC (125 MHz, MeOD)
18b
f1 (ppm)
f2 (ppm)

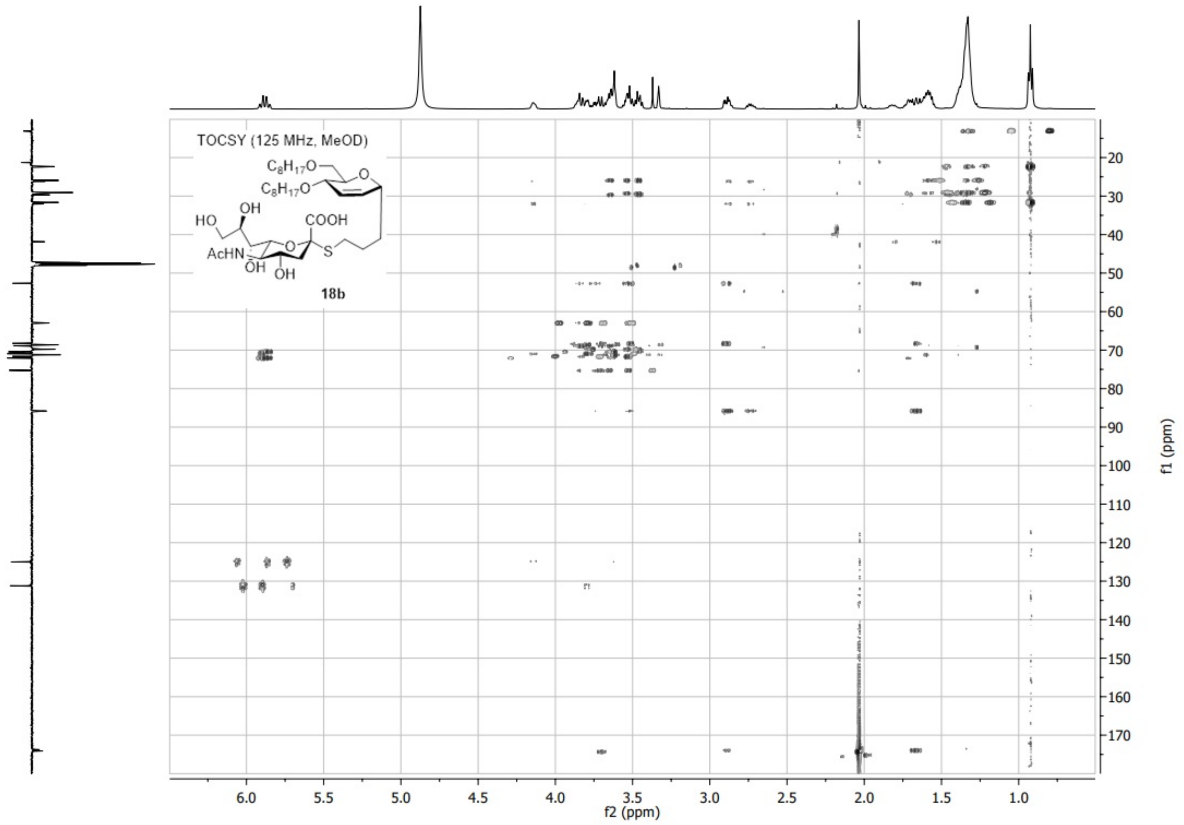
TOCSY (125 MHz, MeOD)
18b
f1 (ppm)
f2 (ppm)

NMR spectra of compound **18c**

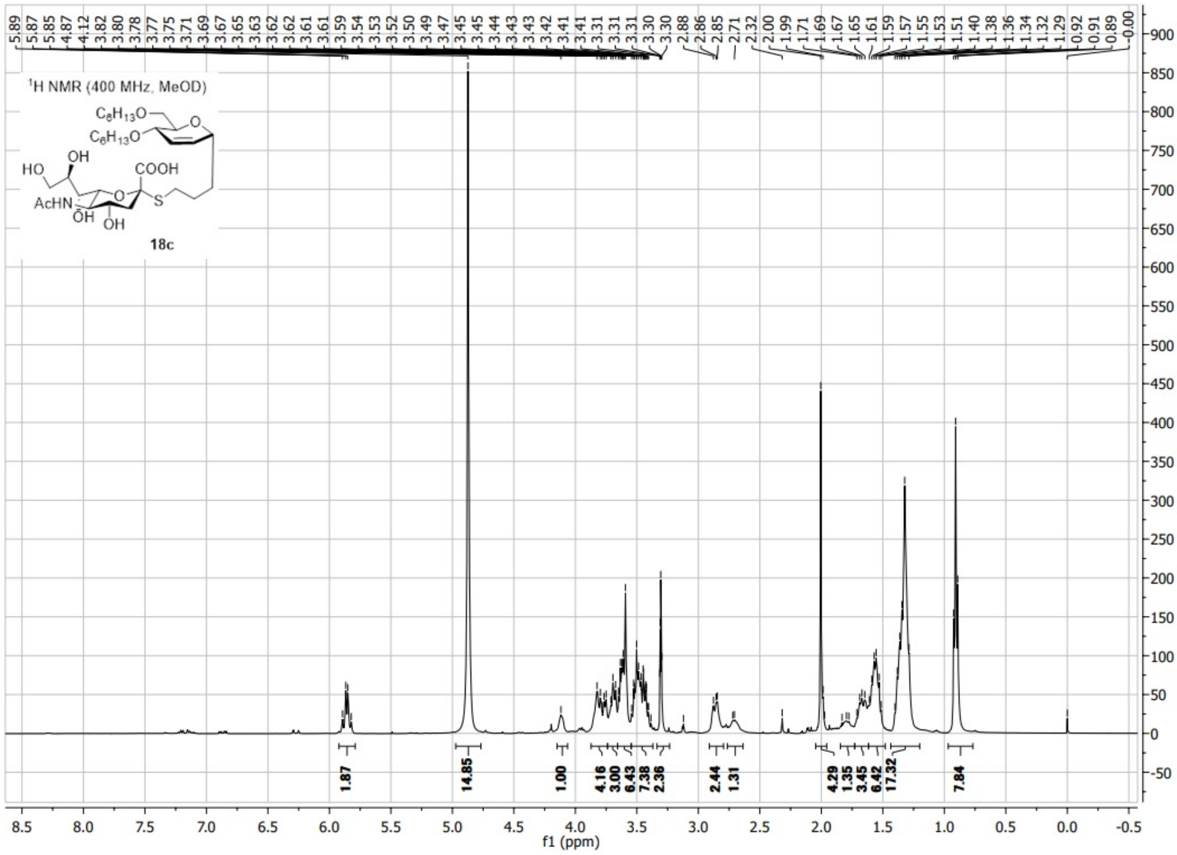

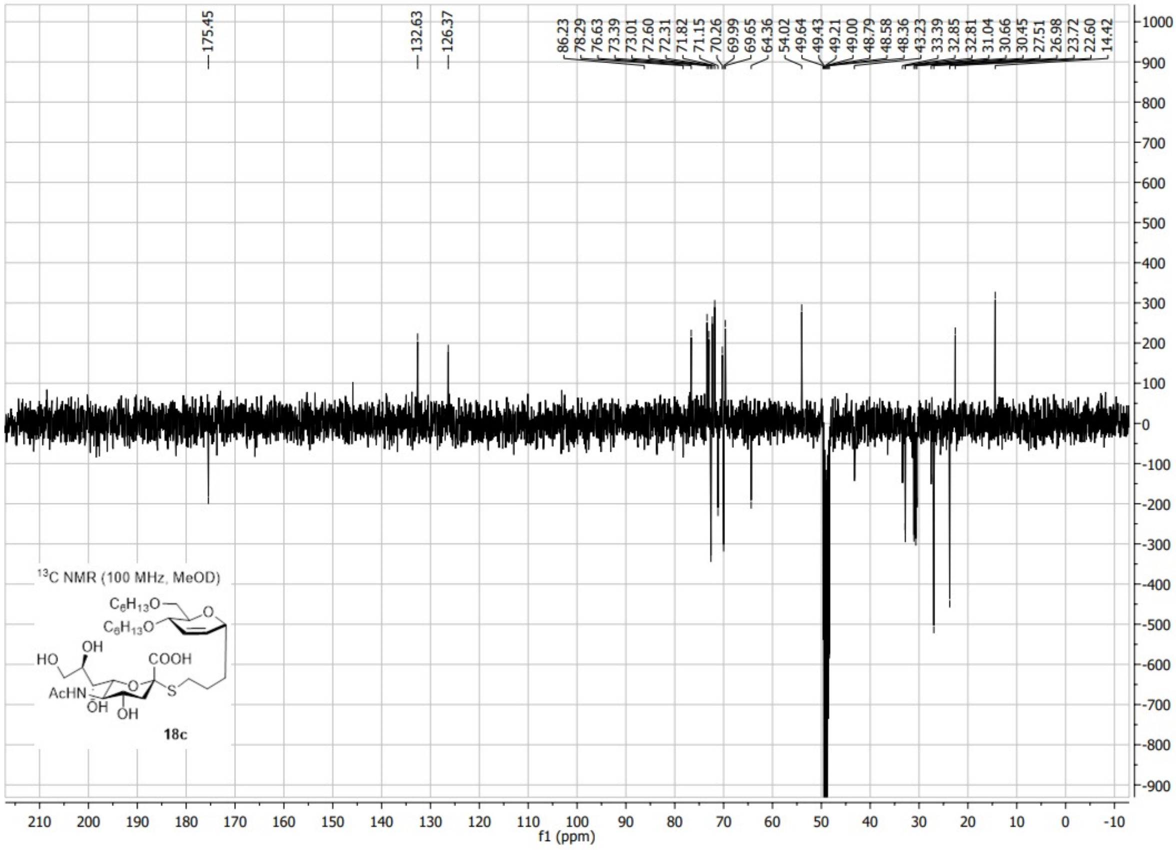

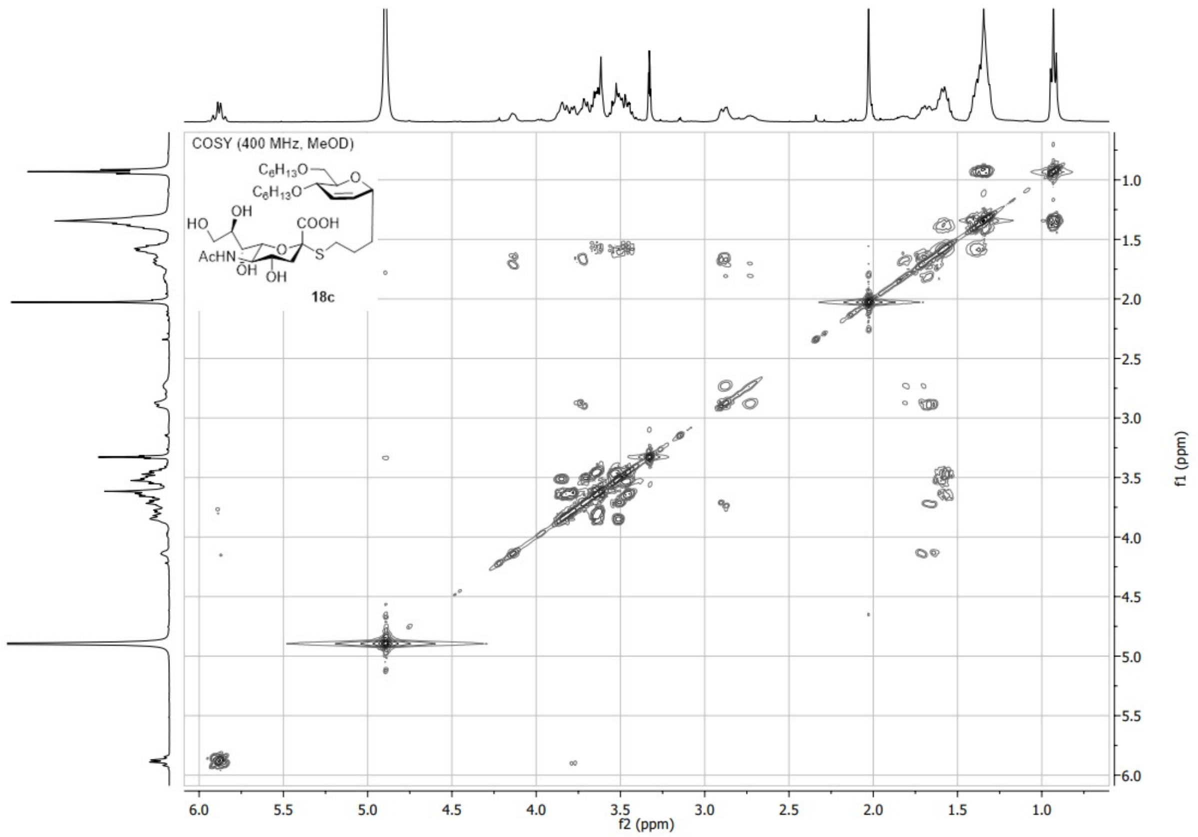
COSY (400 MHz, MeOD)
18c
f2 (ppm)
f1 (ppm)

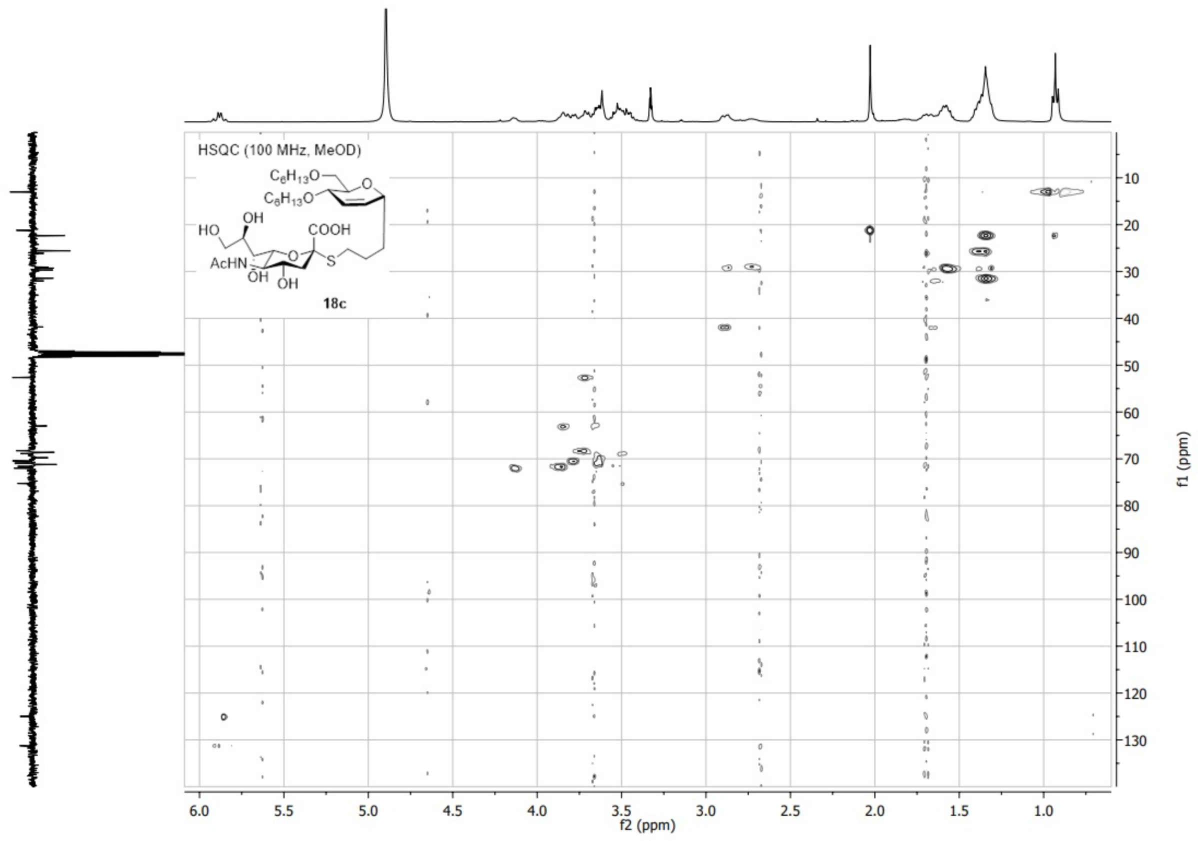
HSQC (100 MHz, MeOD)
18c
f2 (ppm)
f1 (ppm)

NMR spectra of compound **20**

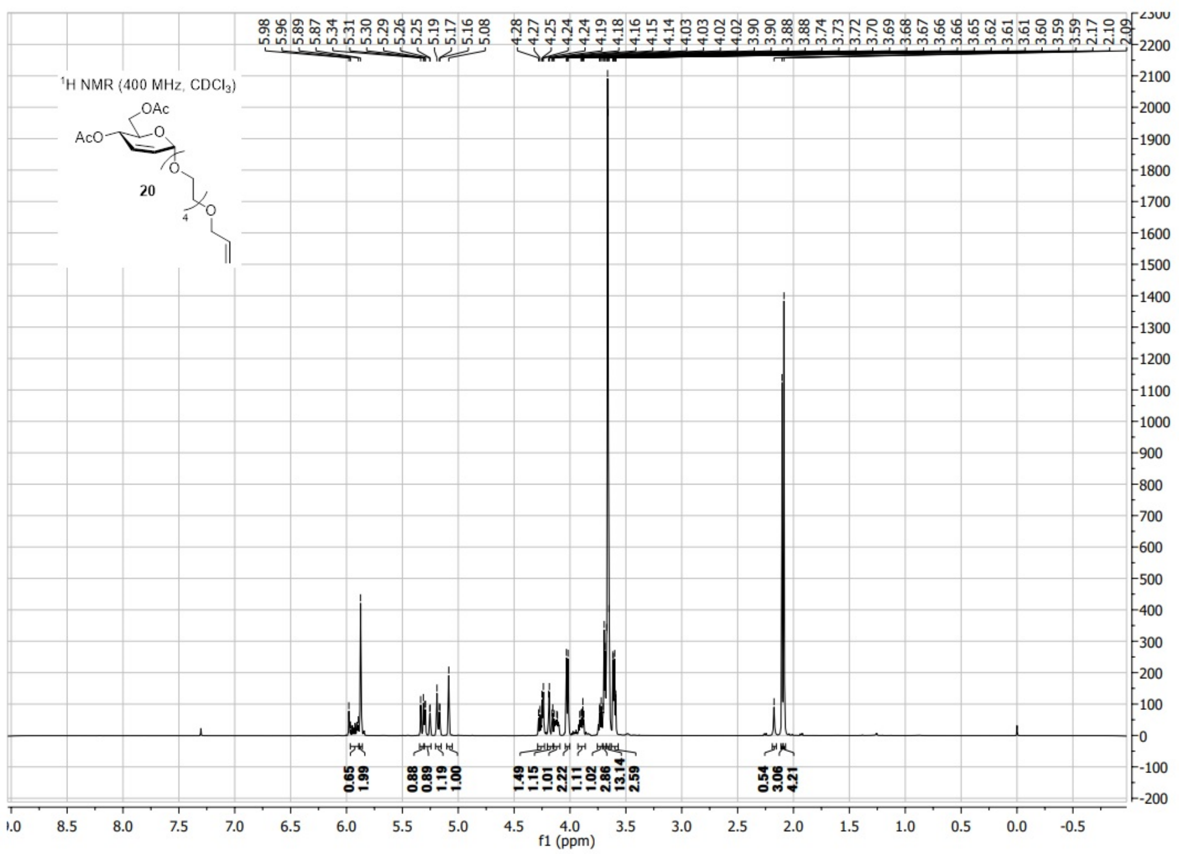

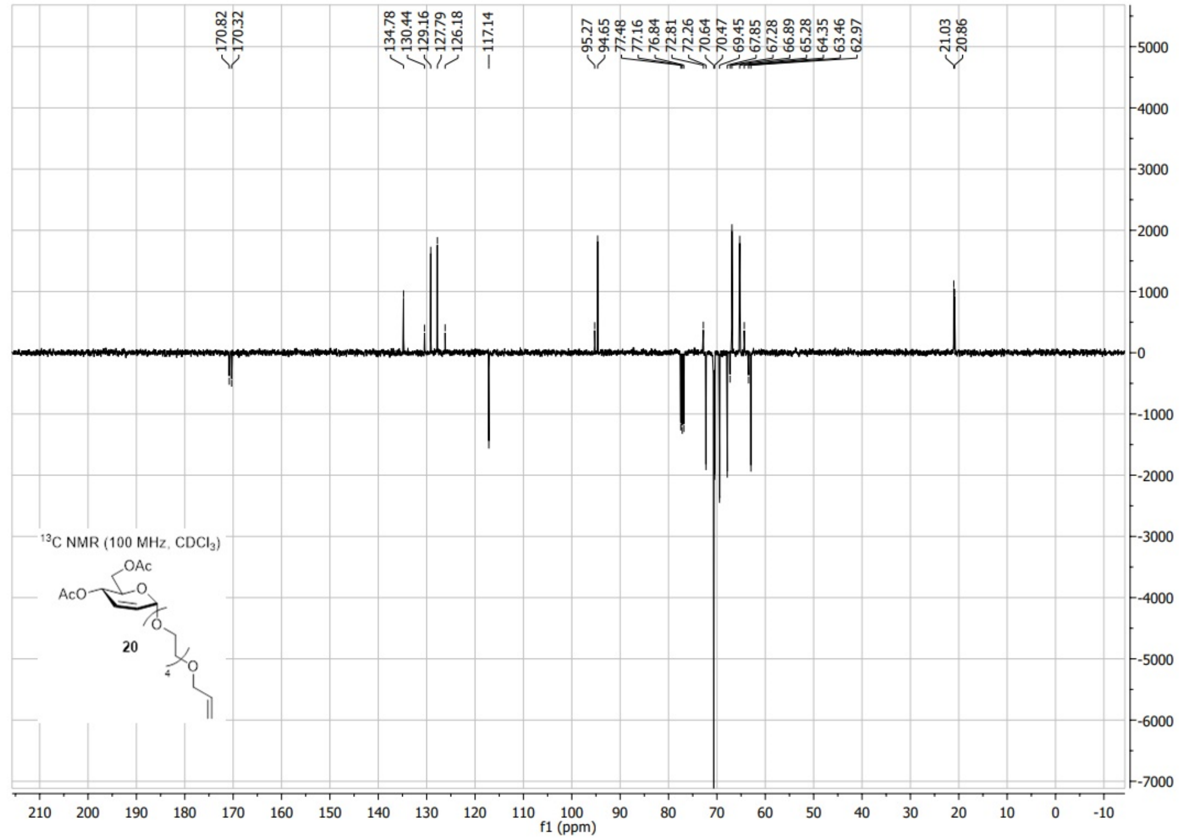

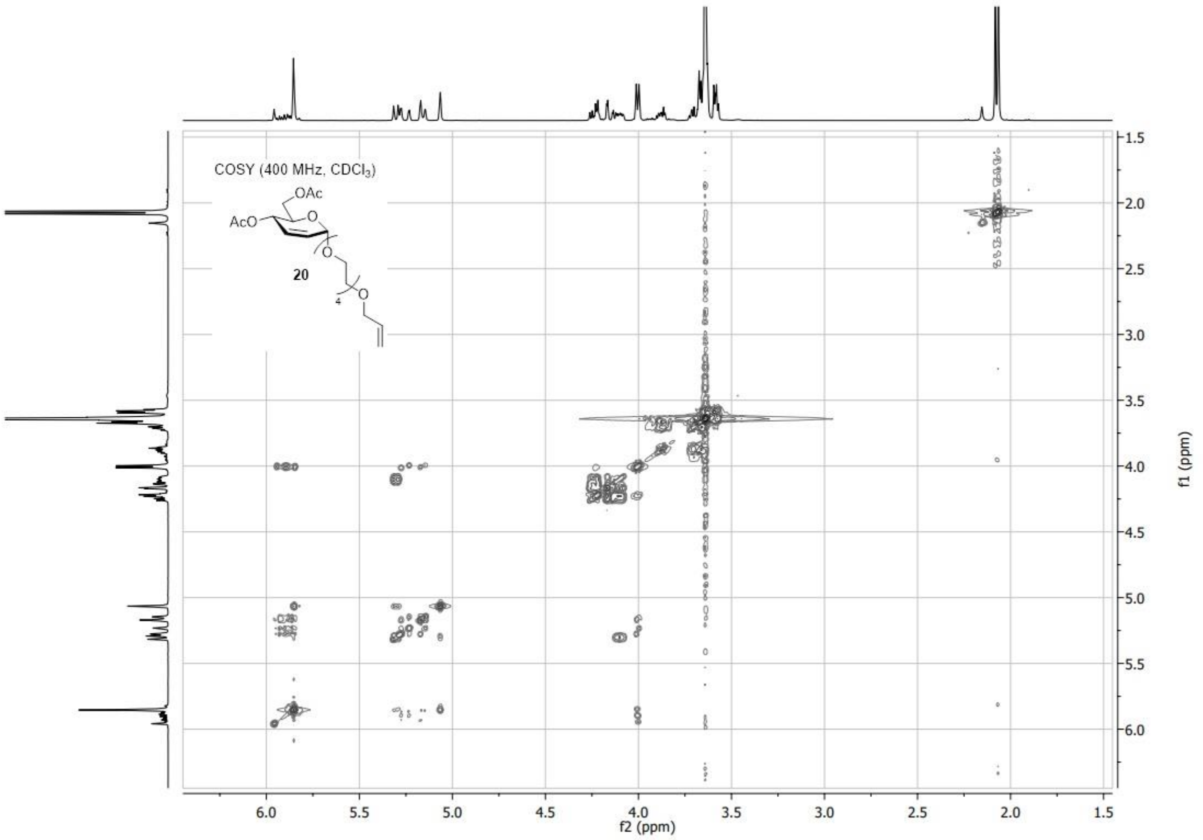
COSY (400 MHz, CDCl3)
OAc
AcO
O
20
O
4
O
f2 (ppm)
f1 (ppm)

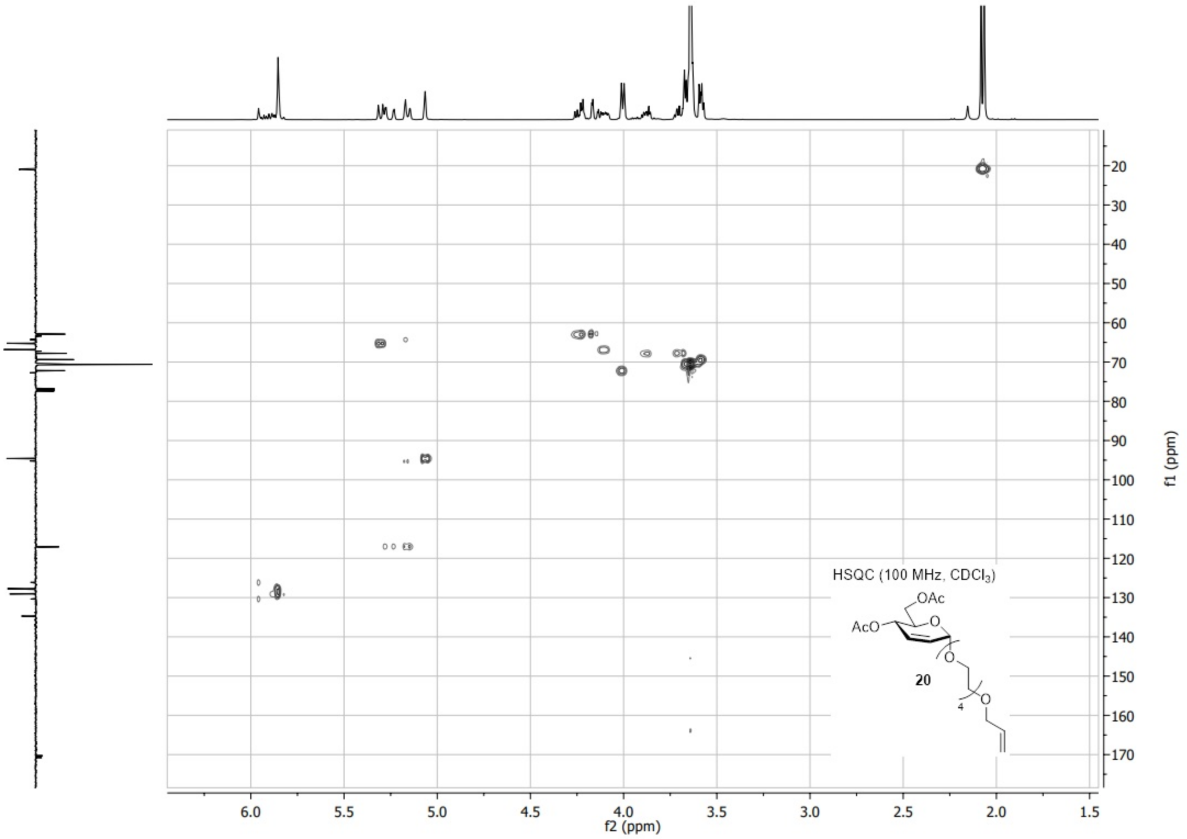
HSQC (100 MHz, CDCl3)
OAc
AcO
O
20
O
4
O
f2 (ppm)
f1 (ppm)

NMR spectra of compound **21a**

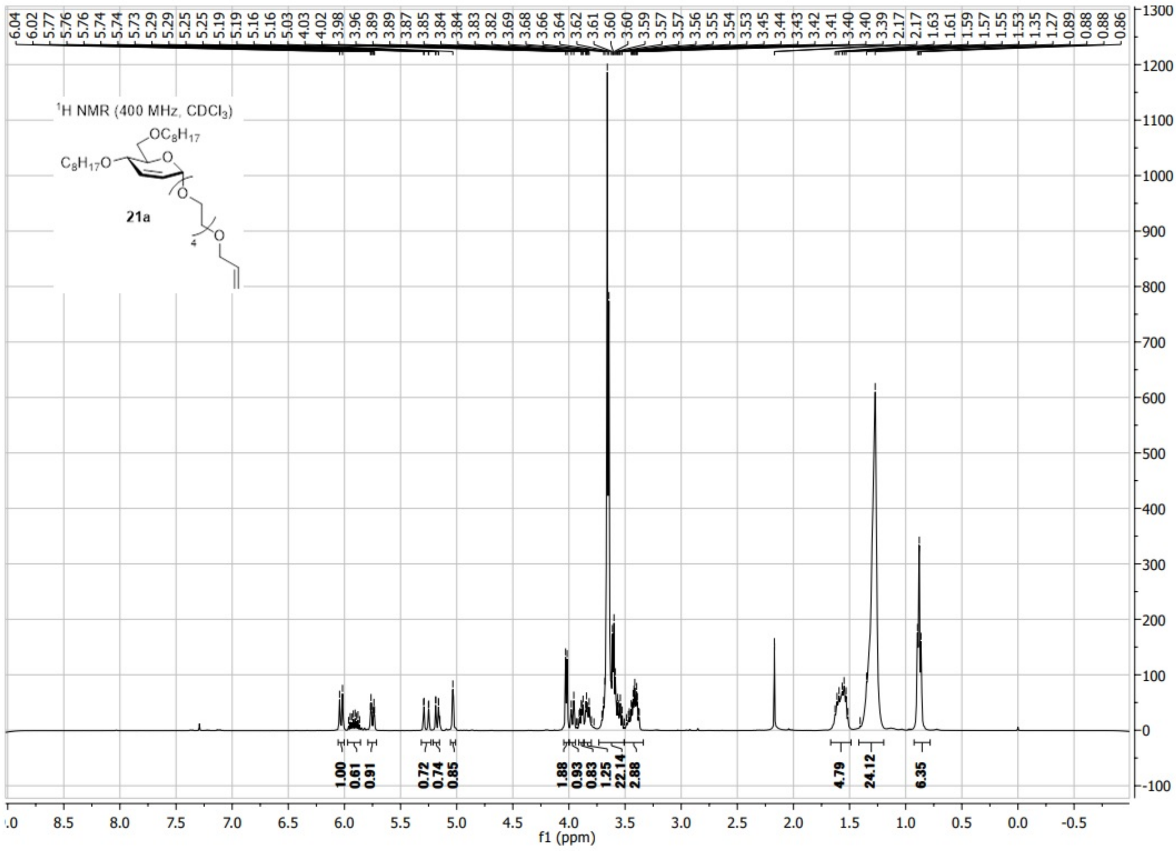

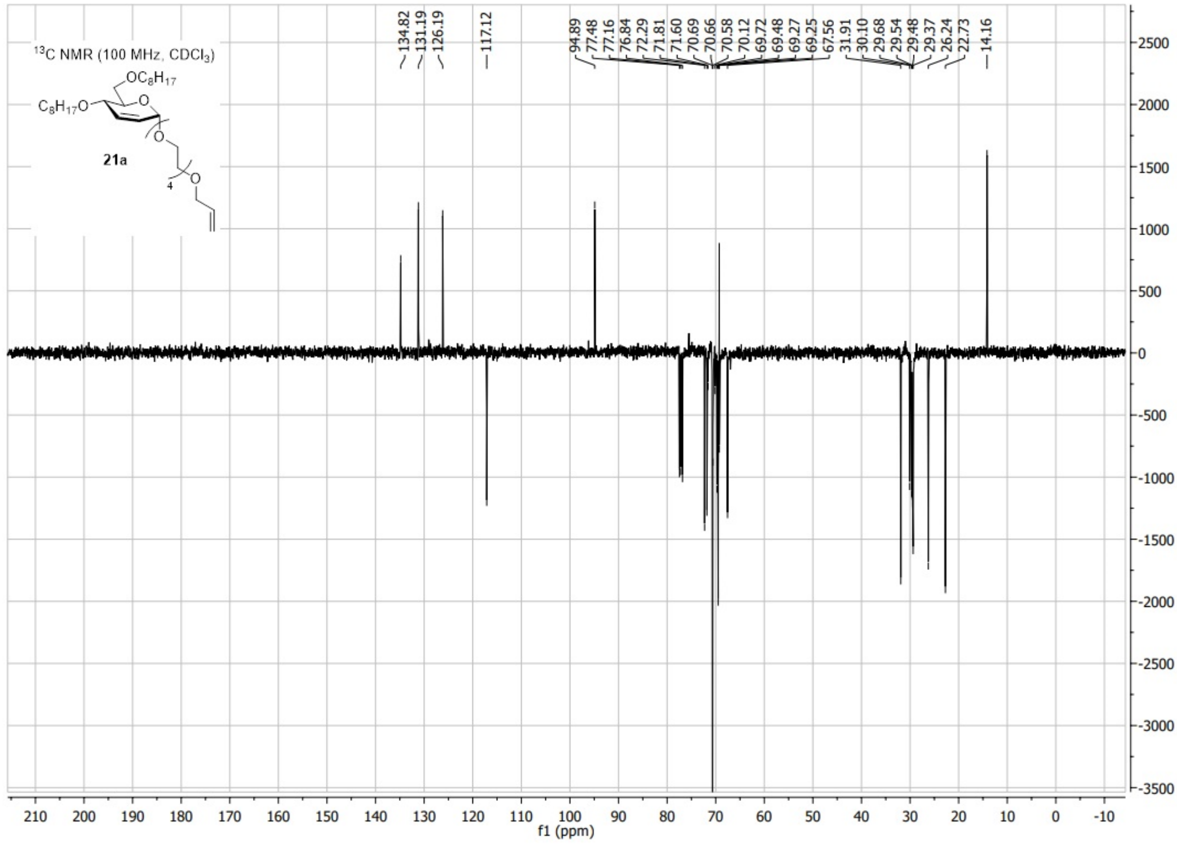

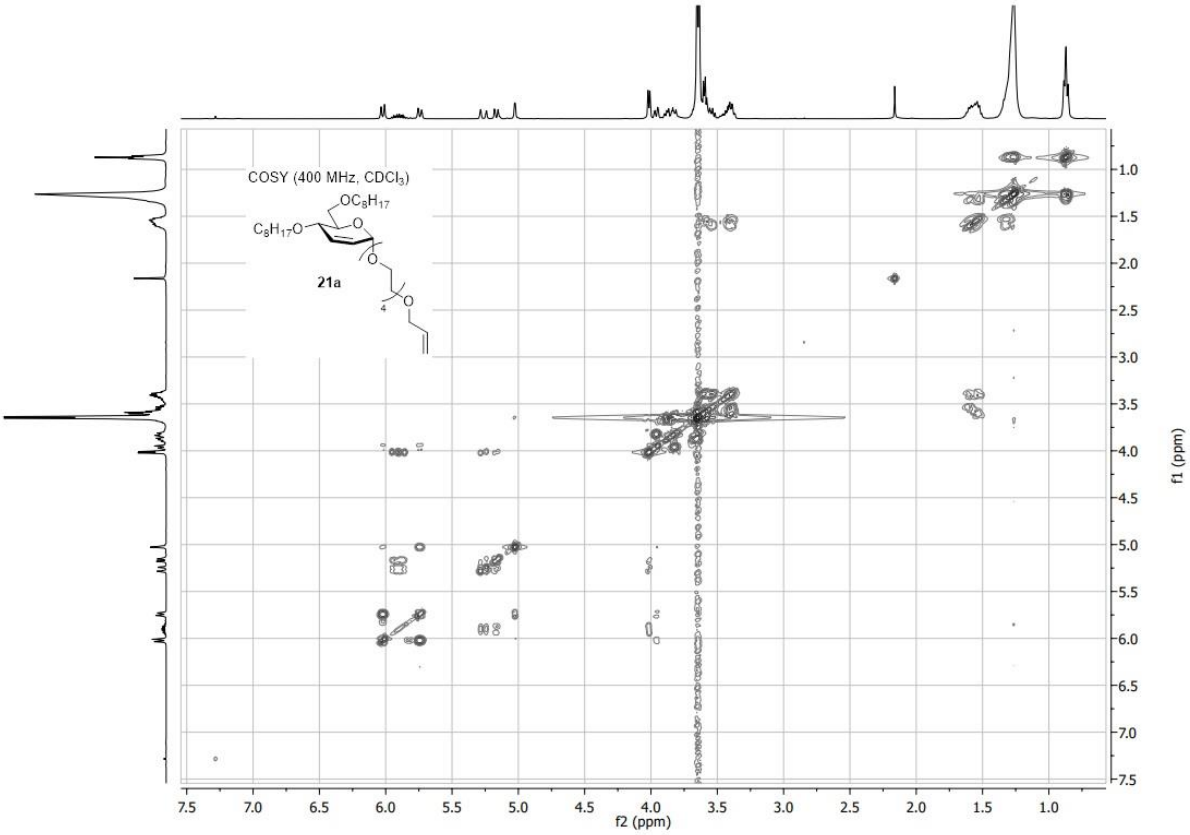
COSY (400 MHz, CDCl3)
OC8H17
C8H17O
O
O
21a
4
O
f1 (ppm)
f2 (ppm)

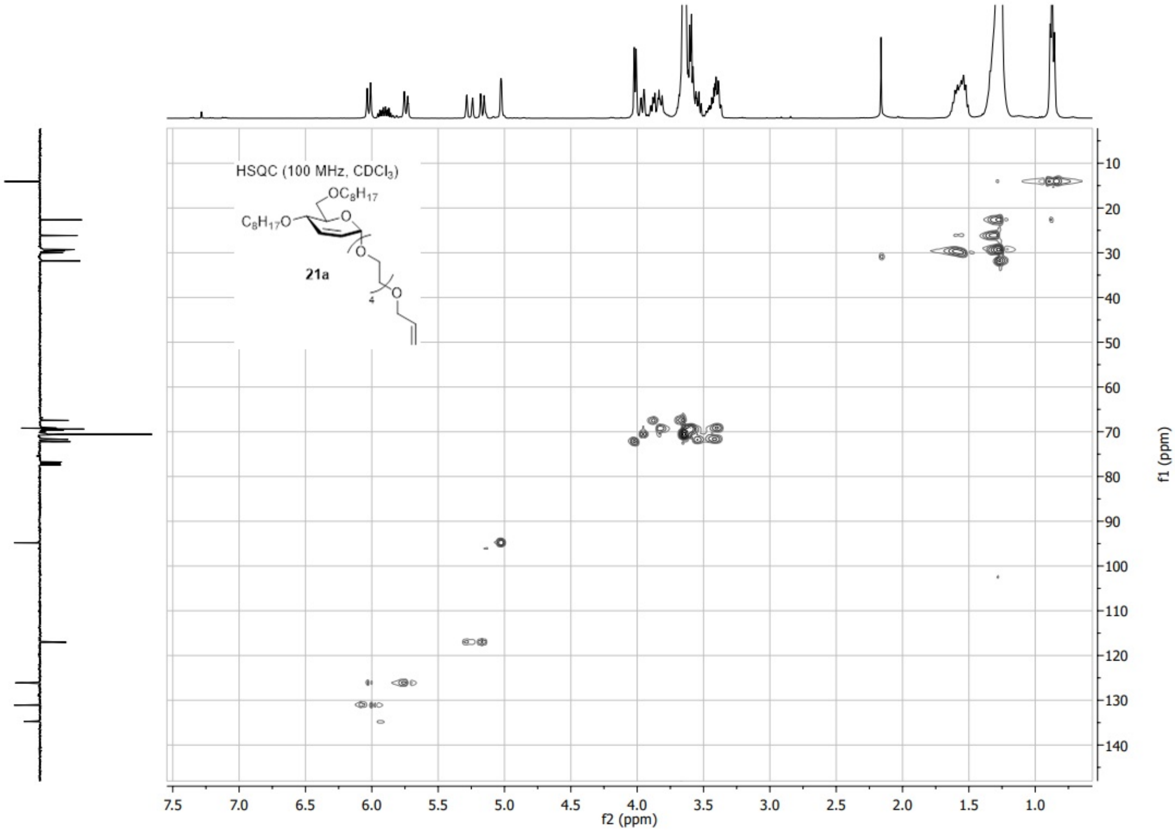
HSQC (100 MHz, CDCl3)
OC8H17
C8H17O
O
O
21a
4
O
f1 (ppm)
f2 (ppm)

NMR spectra of compound **21b**

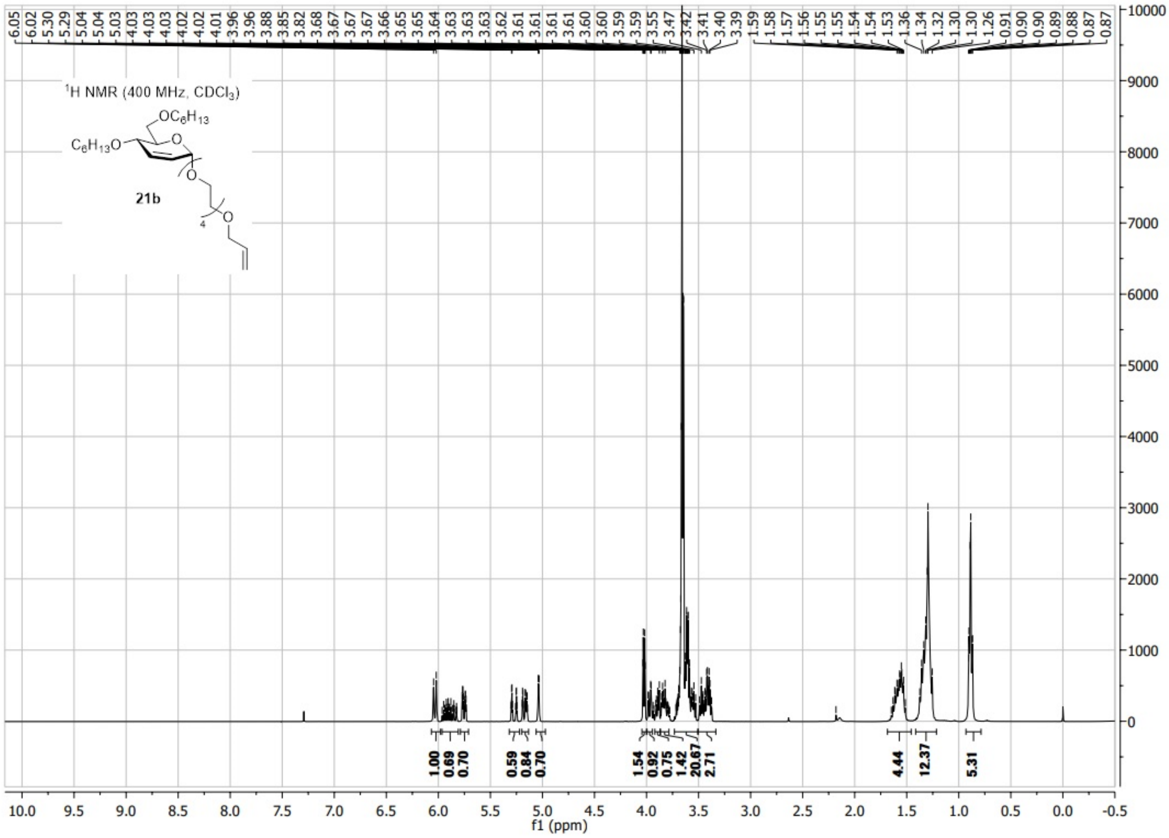

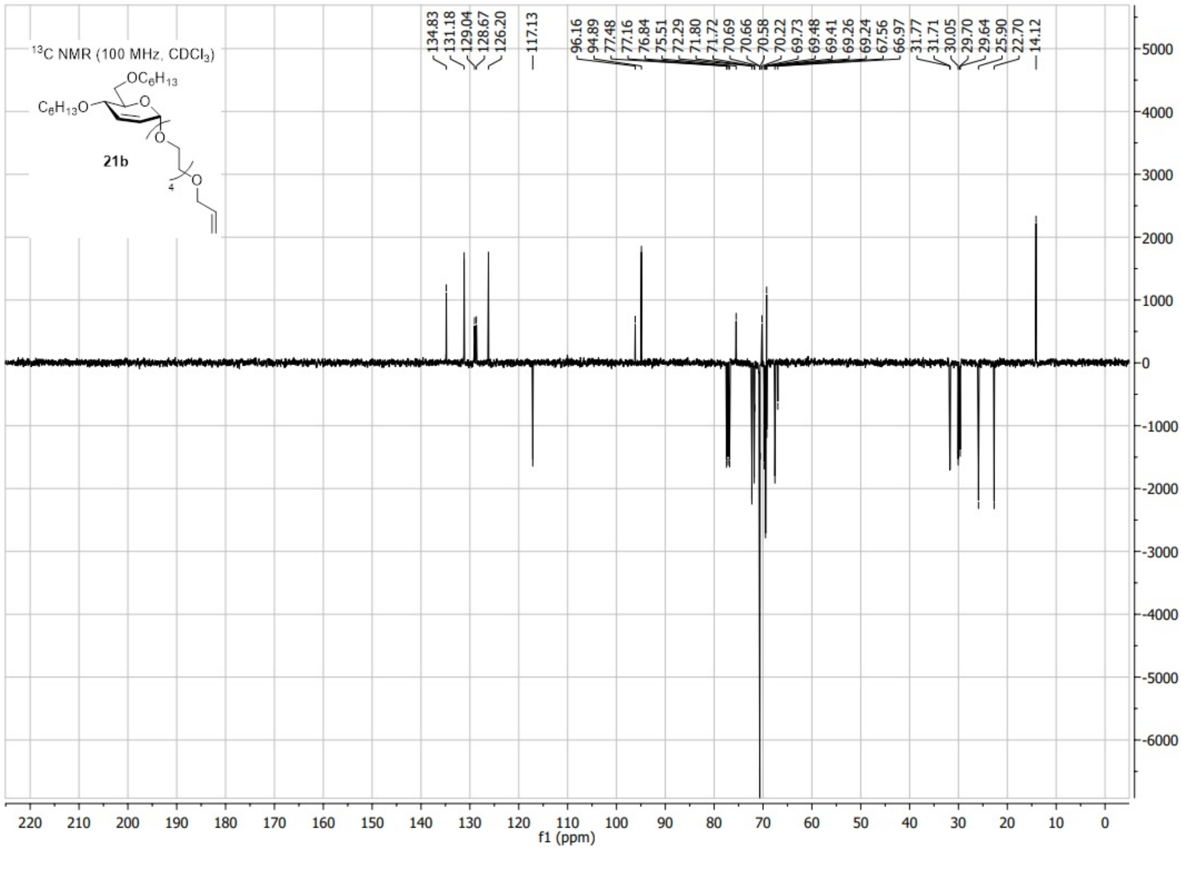

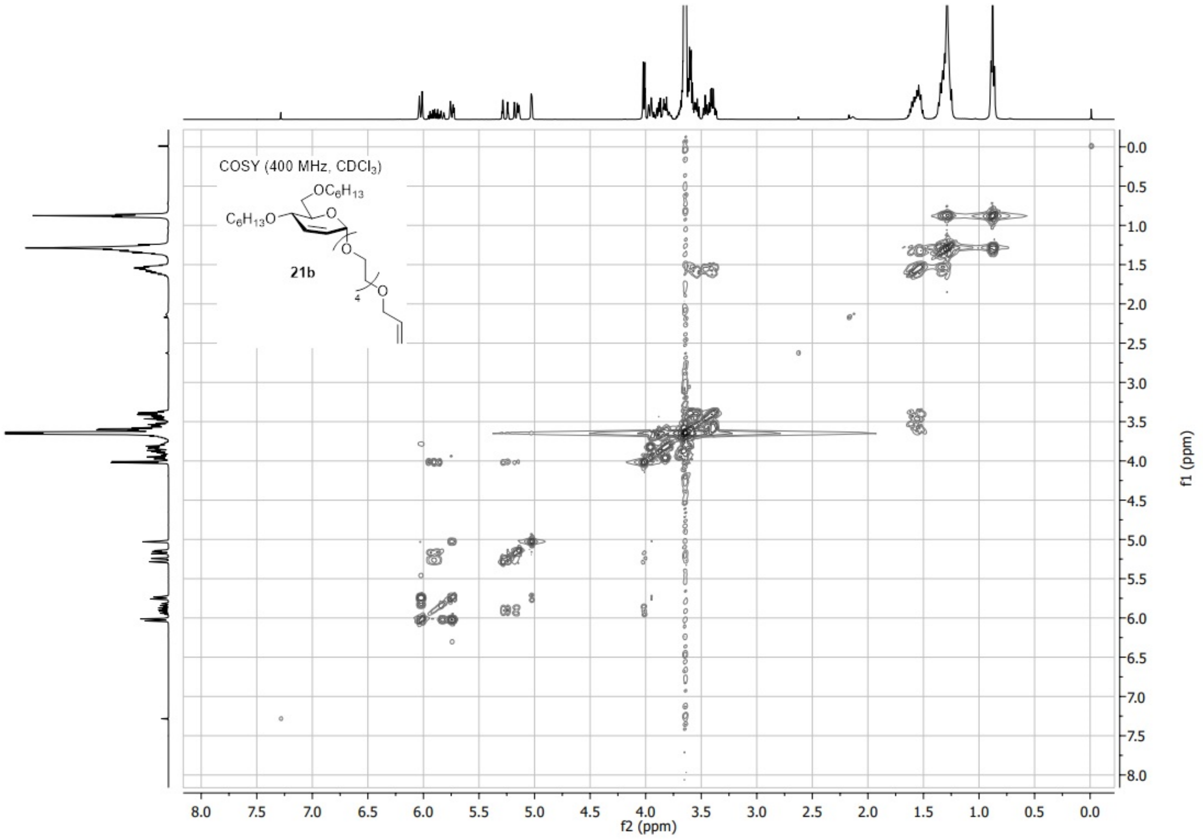
COSY (400 MHz, CDCl$_3$)
OC$_6$H$_{13}$
C$_6$H$_{13}$O
O
O
21b
4
O
f2 (ppm)
f1 (ppm)

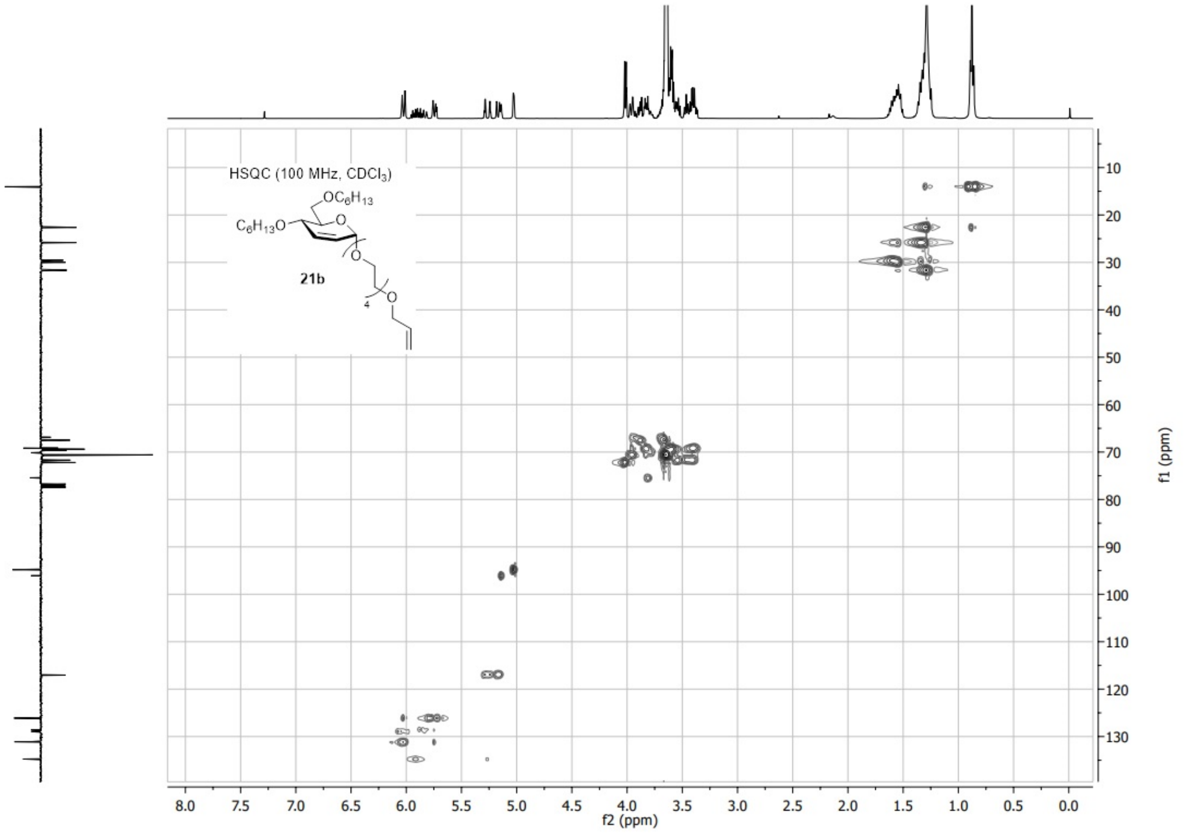
HSQC (100 MHz, CDCl$_3$)
OC$_6$H$_{13}$
C$_6$H$_{13}$O
O
O
21b
4
O
f2 (ppm)
f1 (ppm)

NMR spectra of compound **23a**

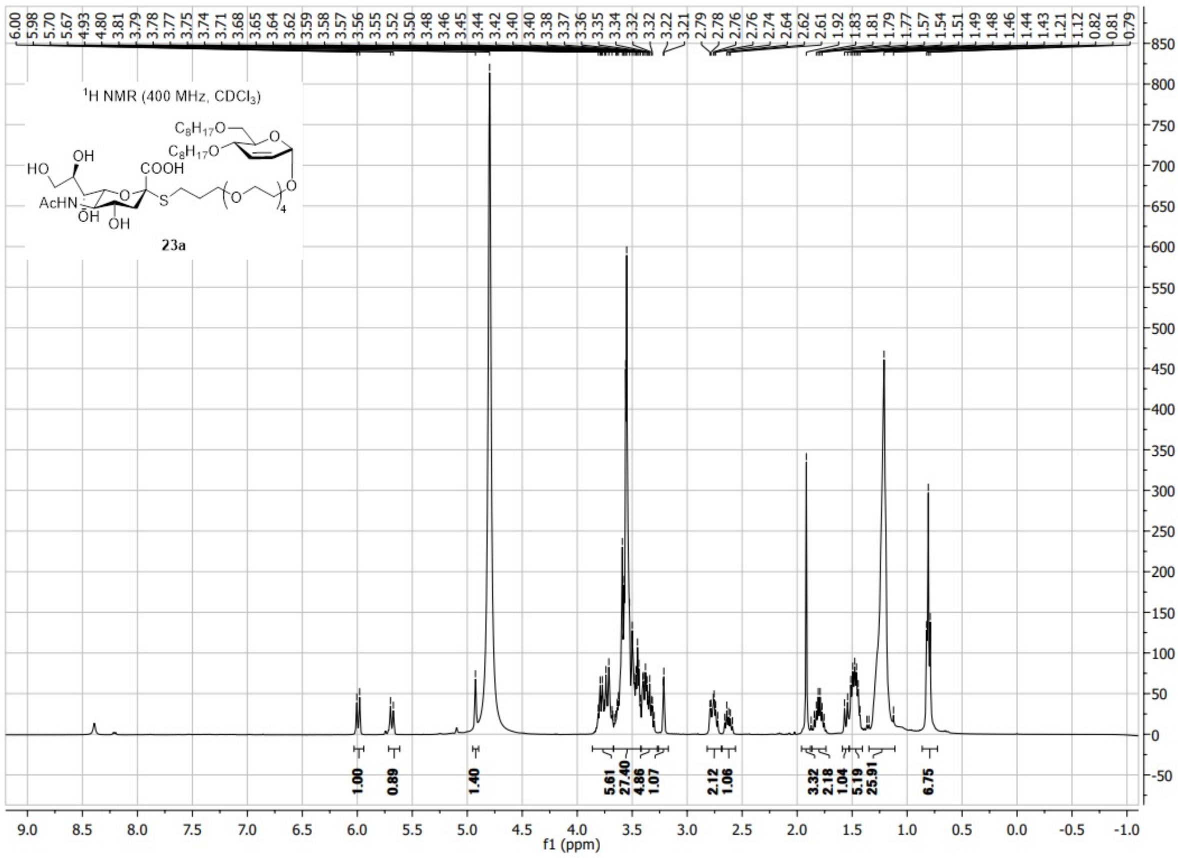

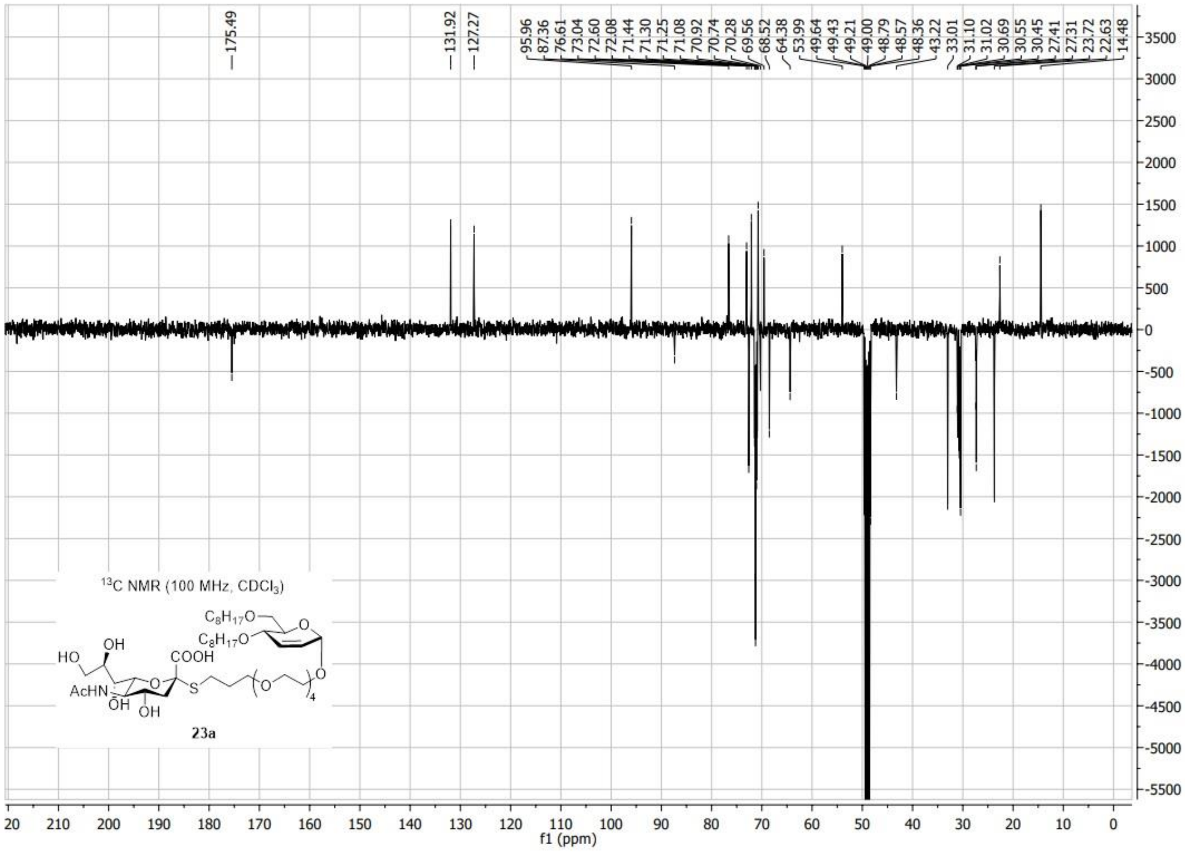

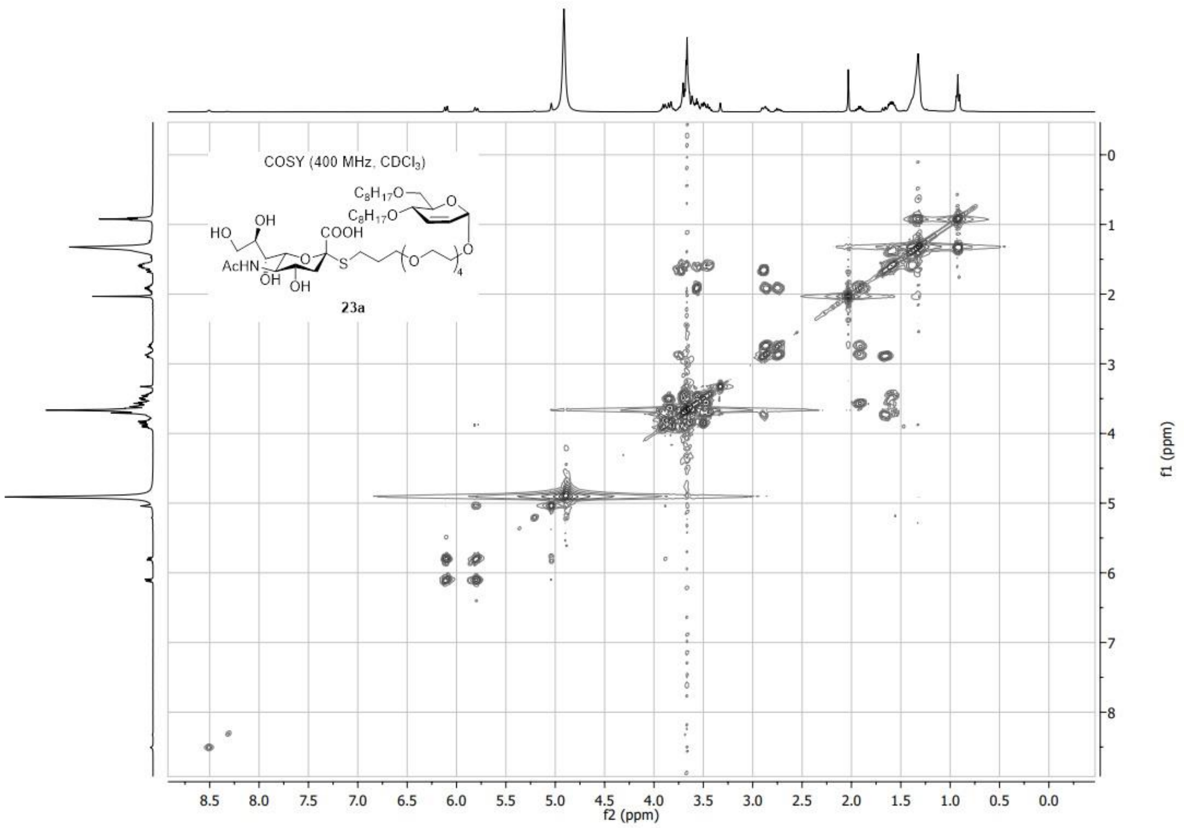
COSY (400 MHz, CDCl3)
23a
f2 (ppm)
f1 (ppm)

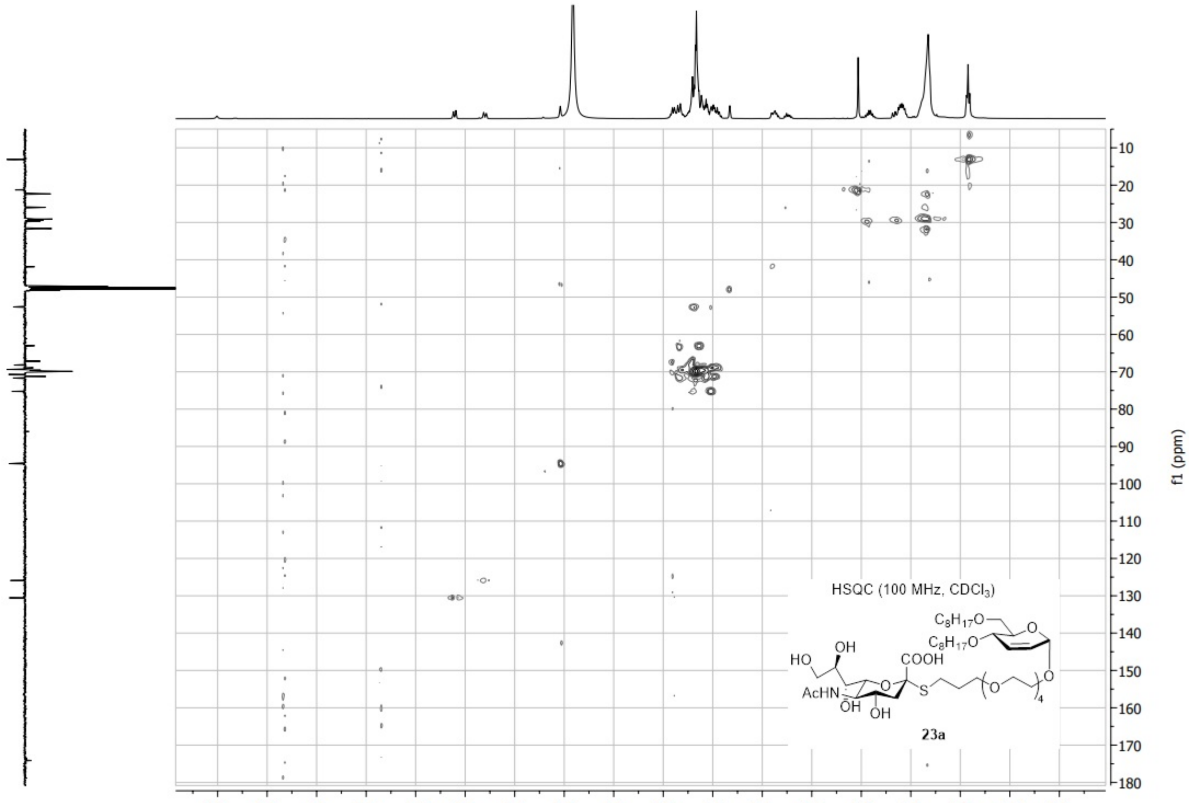
HSQC (100 MHz, CDCl3)
23a
f2 (ppm)
f1 (ppm)

NMR spectra of compound **23b**

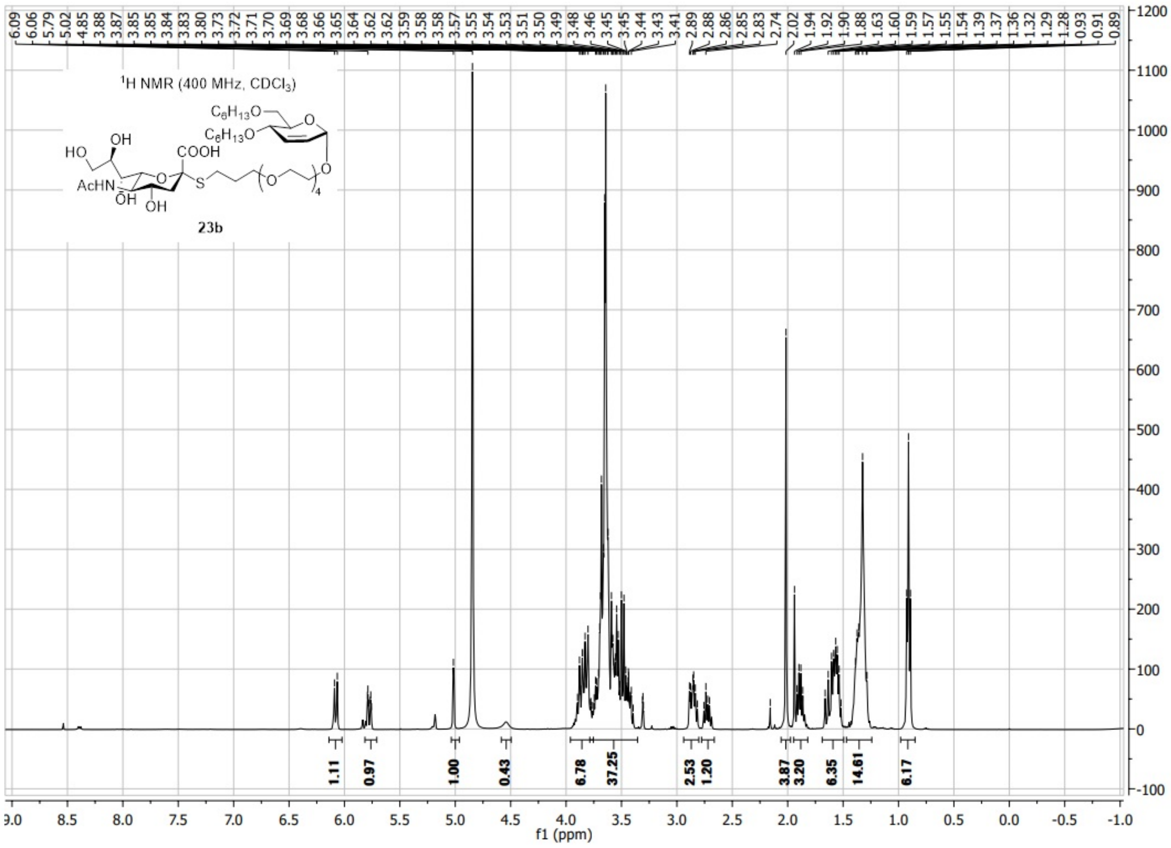

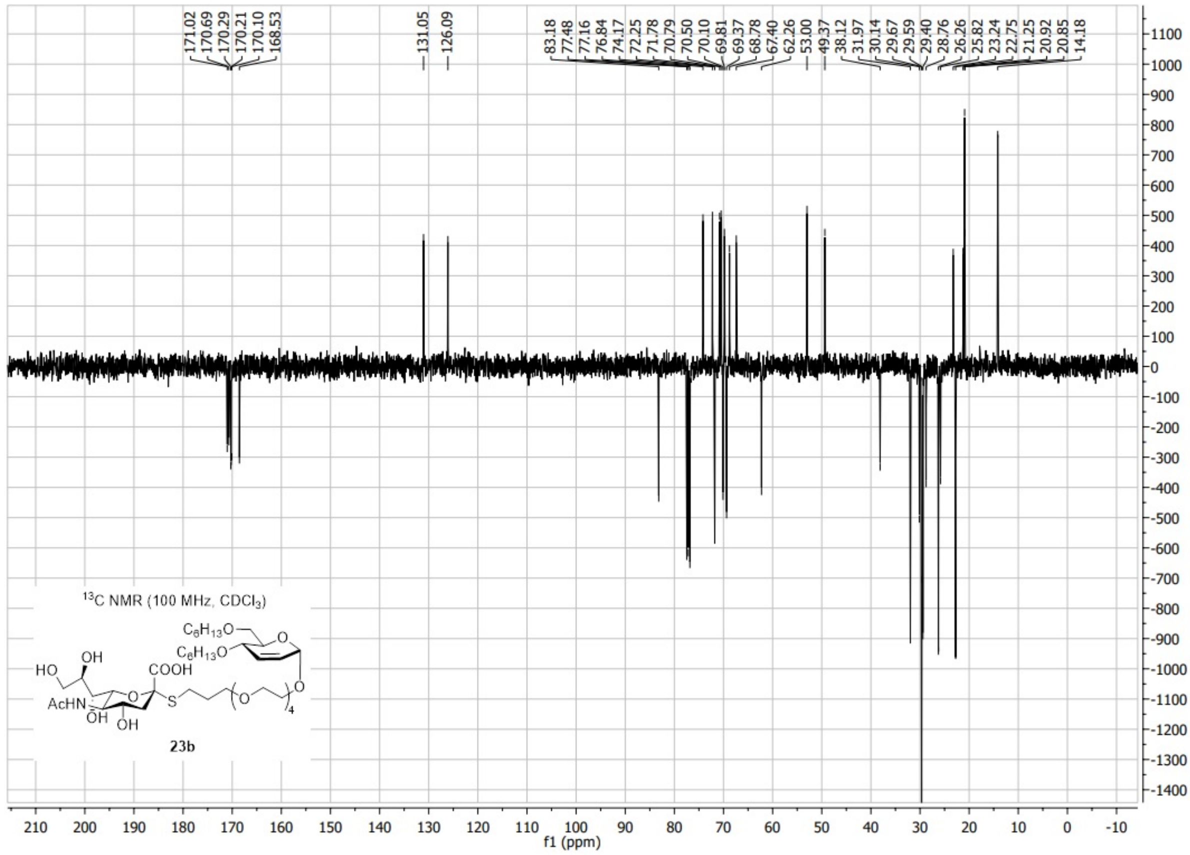

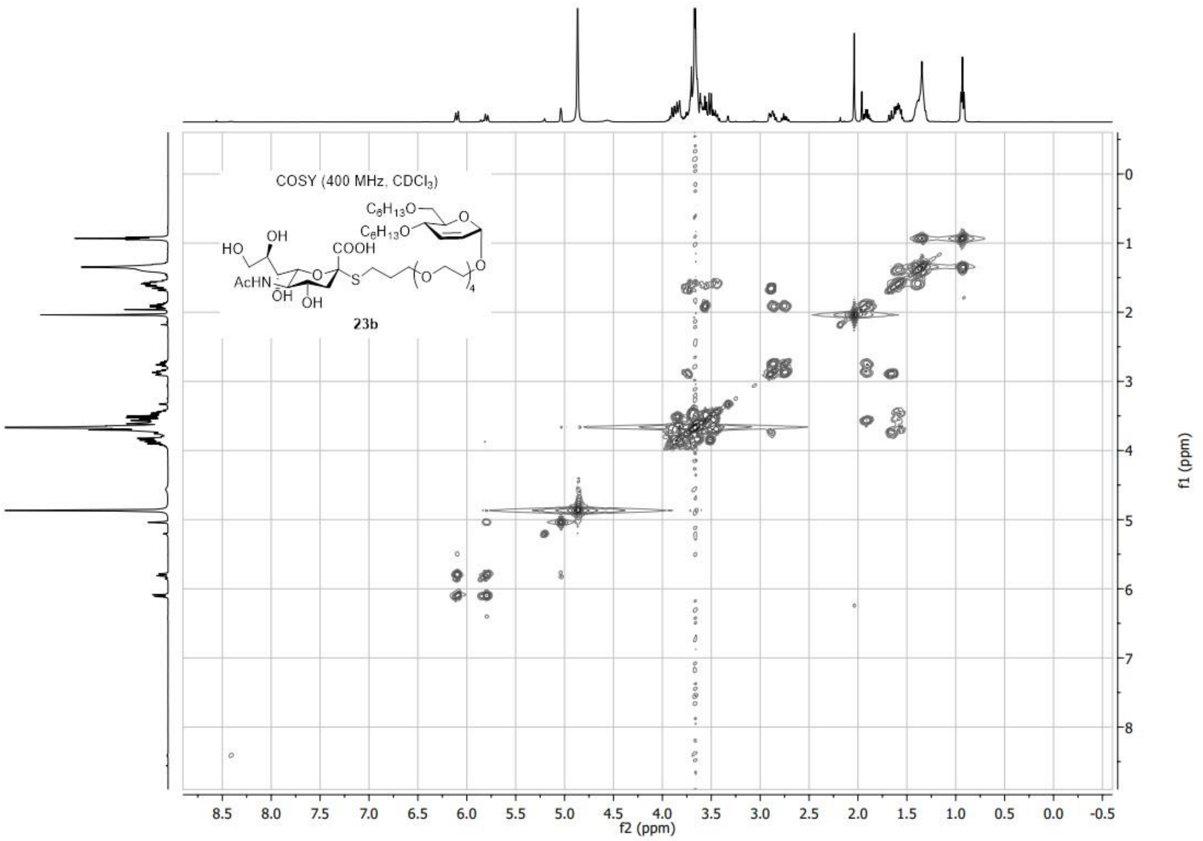
COSY (400 MHz, CDCl3)
23b
f2 (ppm)
f1 (ppm)

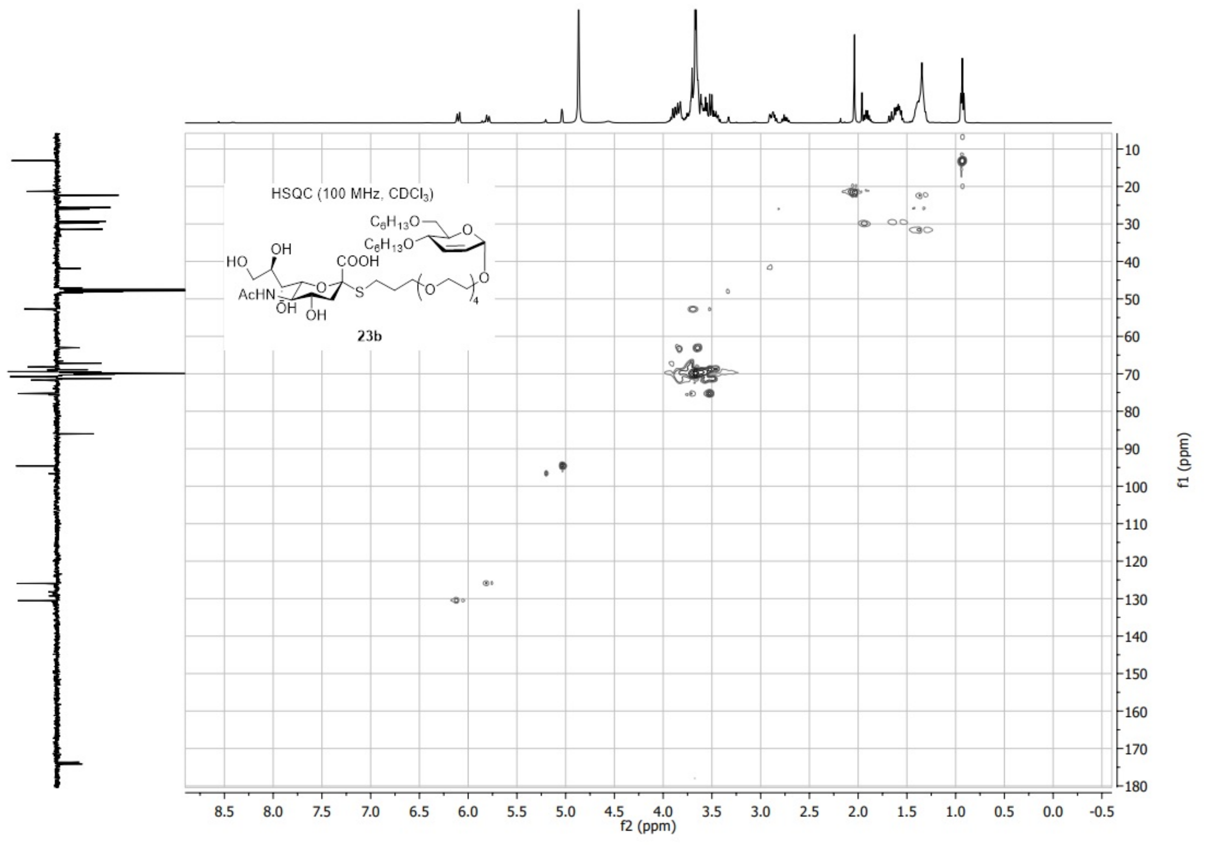
HSQC (100 MHz, CDCl3)
23b
f2 (ppm)
f1 (ppm)
